# Synthesis and Antiviral Activity of a Series of 2′-*C*-Methyl-4′-thionucleoside Monophosphate Prodrugs

**DOI:** 10.3390/molecules25215165

**Published:** 2020-11-06

**Authors:** Zackery W. Dentmon, Thomas M. Kaiser, Dennis C. Liotta

**Affiliations:** 1 Department of Chemistry, Emory University, 1521 Dickey Dr., Atlanta, GA 30322, USA; zack.dentmon@emory.edu; 2 St. Peter’s College, University of Oxford, Oxford OX1 2DL, UK; thomas.kaiser@spc.ox.ac.uk

**Keywords:** prodrugs, nucleosides, nucleotides, antivirals, medicinal chemistry, hepatitis C

## Abstract

The NS5B RNA-dependent RNA polymerase of the hepatitis C virus (HCV) is a validated target for nucleoside antiviral drug therapy. We endeavored to synthesize and test a series of 4′-thionucleosides with a monophosphate prodrug moiety for their antiviral activity against HCV and other related viruses in the Flaviviridae family. Nucleoside analogs were prepared via the stereoselective Vorbrüggen glycosylation of various nucleobases with per-acetylated 2-*C*-methyl-4-thio-d-ribose built in a 10-step synthetic sequence from the corresponding ribonolactone. Conjugation of the thionucleoside to a ProTide phosphoramidate allowed for evaluation of the prodrugs in the cellular HCV replicon assay with anti-HCV activities ranging from single-digit micromolar (μM) to >200 μM. The diminished anti-HCV potency of our best compound compared to its 4′-oxo congener is the subject of ongoing research in our lab and is proposed to stem from changes in sugar geometry imparted by the larger sulfur atom.

## 1. Introduction

**Figure 1:**
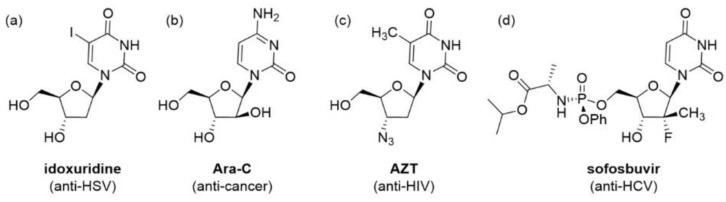
Examples of early modified nucleoside drugs (**a**–**c**) and the aryloxy phosphoramidate monophosphate prodrug sofosbuvir (**d**).

Scientists have long been interested in the medicinal applications of non-canonical nucleosides and nucleotides due to their privileged bioactivity. Beginning in the late 1950s with idoxuridine (5-iodo-2′-deoxyuridine, see Figure 1a, modified nucleosides and nucleotides have found use predominantly as anti-neoplastics and antivirals for their ability to inhibit nucleic acid polymerization and replication by competing with nucleoside triphosphates (NTPs), the natural substrates of nucleic acid polymerases [1,2]. Cytarabine (arabinocytidine, Ara-C) was first approved by the FDA in 1969 and is still considered by the World Health Organization to be an essential medicine for cancers of the blood [3]. Likewise, 3′-azidothymidine (AZT), the first approved treatment against human immunodeficiency virus (HIV), also appears on this list along with a dozen other nucleos(t)ide analogs acting as antivirals alone [2,3,4].

One such compound is sofosbuvir, a monophosphate prodrug of 2′-β-methyl-2′-α-fluorouridine (Figure 1d). Approved in late 2013 for the treatment of chronic hepatitis C virus (HCV) infection, sofosbuvir has altered the landscape of HCV therapy [5,6]. No fewer than 15 nucleos(t)ide analogs have gone into clinical trials for HCV since the early 2000s, and only sofosbuvir has been approved by both the US FDA and EU regulatory agencies. Furthermore, nearly all of the aforementioned analogs share the common 2′-β-methyl sugar scaffold originally disclosed by Merck in 2003 [7]. In addition to disrupting RNA chain elongation, subsequent studies showed the value of the 2′-methyl group in conferring selectivity of this class for the non-structural protein (NS5B) viral RNA-dependent RNA polymerase (RdRp) over host RNA polymerase [8,9,10,11]. Sofosbuvir also bears the hepatic-directing ProTide monophosphate prodrug moiety pioneered by Christopher McGuigan and co-workers at Cardiff University [12,13]. Their lab has utilized ProTide technology to turn inactive parent nucleosides into sub-micromolar inhibitors of HCV replicons [14,15]. Chemical incorporation of the first 5′-*O*-phosphoryl moiety with an easily metabolized phosphoramidate creates a monophosphate pronucleotide, bypassing the particularly discriminating first kinase in the phosphorylation cascade, which is known to be a culprit of poor anabolism and low levels of NTP [16,17,18]. It stands to reason then that these two structural features (2′-β-methyl and 5′-phosphoramidate prodrug moieties) would be key design elements in the interrogation of next-generation NS5B inhibitors.

What structural feature(s) would set the next generation of nucleos(t)ide analogs apart from sofosbuvir? According to Michael Sofia, co-inventor of sofosbuvir, to compete with sofosbuvir a molecule would need to deliver an advantage in either its resistance profile or its pharmacokinetics (PK) [6]. With regards to PK, the issues involve the interaction with intestinal *P*-glycoprotein and catabolism to the major metabolite–the corresponding dephosphorylated uridine nucleoside, which is cleared through renal secretion. These issues pose opportunities for a compound with alternative metabolism leading to lower renal clearance, longer systemic half-life, and decreased treatment duration. Hence, alternative nucleobases and sugar modifications should be explored to this end.

**Figure 2:**
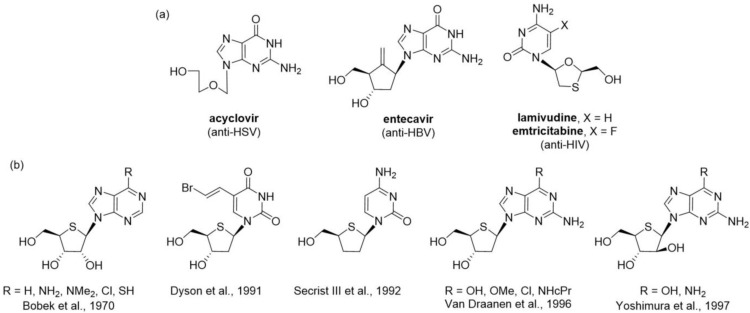
Representative examples of (**a**) essential antiviral nucleosides with non-natural sugar ring compositions, and (**b**) historical 4′-thionucleoside analogs having notable bioactivity.

Nucleosides with non-natural sugar ring composition have been successfully used to treat other viral diseases (Figure 2a). Acyclovir and tenofovir are acyclic nucleoside analogs while abacavir and entecavir are carbocyclic examples, all of which are essential antiviral medicines against herpes simplex virus (HSV), HIV, or HBV. In addition, lamivudine and emtricitabine are also on this list against HIV and they contain a 3′-thia substitution in the ring, the latter of which was discovered in our lab [3,19]. Replacement of the furanose 4′-oxygen with bioisosteric sulfur has also attracted some attention (Figure 2b) [20,21]. Bobek first synthesized 4′-thionucleosides in the early 1970s, which showed interesting antibiotic and anti-cancer activity [22,23]. In the 1990s, Dyson et al. showed antiviral activity of 2′-deoxy-4′-thiopyrimidines against HSV, Varicella Zoster virus (VZV), and cytomegalovirus (CMV) [24], while Secrist III showed anti-HIV activity with 2′,3′-dideoxy-4′-thionucleosides [25]. Van Draanen and co-workers showed promising activity against HBV and CMV with 2′-deoxy-4′-thiopurine analogs [26]. Most of these authors make note of the differential metabolism of the sulfur analog compared to the 4′-oxo congener, in particular the resistance of the 4′-thio analogs to nucleoside phosphorylase. In one of the more provocative examples of the 4′-thio substitution in the literature, Yoshimura and colleagues showed that the swap from oxygen to sulfur in the sugar ring conferred low- to sub-micromolar antiviral activity onto previously inactive arabinopurines against HSV and CMV [27]. More recently in the context of HCV, a 2006 patent application by Merck disclosed 2′-β-methyl-4′-thiopurines for use against NS5B [28]. In 2014 Idenix filed a patent claiming the triphosphates of 4′-thioguanosine, 4′-thiouridine, and 2′-β-methyl-4′-thiouridine, as well as monophosphate prodrugs of the latter two, for the treatment of HCV [29]; they were acquired by Merck that same year, purportedly for their anti-HCV portfolio. It was out of this context that we became interested in supplementing the work of Idenix on this intriguing chemotype.

## 2. Results and Discussion

### 2.1. Synthesis of the 4′-Thionucleosides Phosphoramidate Prodrugs

#### 2.1.1. Synthesis of the Thiosugar Core

**Scheme 1:**
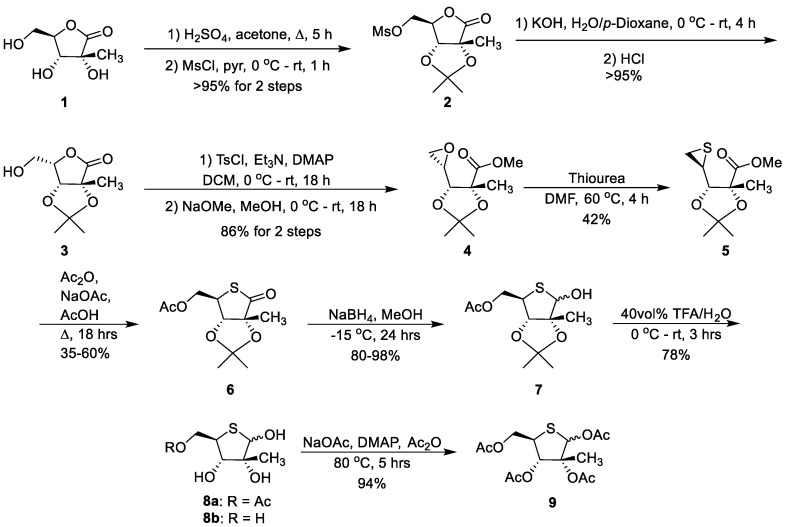
Multistep synthesis of the per-acetylated thiosugar core.

The synthesis of the target nucleosides began with the construction of the thiosugar core. Following the precedent disclosed by Dukhan and co-workers [30], the synthesis started from the chiral building block 2-methyl-d-ribono-1,4-lactone (1, Scheme 1). After protecting the 2,3-diol system as an acetonide, the remaining primary alcohol at C5 was activated for displacement by conversion to the corresponding mesylate. Mesylate 2 presumably undergoes intramolecular attack by the 4-alkoxide liberated by selective saponification of the lactone with aqueous KOH. The resulting intermediate 4,5-epoxide is not isolated but protonated and hydrolyzed in situ upon treatment with aqueous HCl, resulting in subsequent acid-mediated lactonization and inversion of the stereocenter at C4. This inversion to the l-lyxonolactone 3 is necessary for overall retention of stereochemistry after an ensuing inversion to install the sulfur atom (vide infra). The addition of 1,4-dioxane as co-solvent for this reaction improved the yield from 55% as reported by Dukhan et al. [30] to essentially quantitative. Then, the primary alcohol was again activated and displaced upon transesterification of the lactone with NaOMe to form the 4,5-epoxide. Treatment of epoxide 4 with thiourea incorporated the desired sulfur atom at C4 with inversion of stereochemistry, though the yield never improved beyond 50% despite substantial optimization efforts. Opening of the resulting thiirane 5 with acetate under refluxing acidic conditions was complicated by regioselectivity issues, producing a separable mixture of both the five- and six-membered thiolactones, accounting for another poorly yielding step even after extensive optimization. The desired five-membered thiolactone 6 was smoothly reduced to the corresponding thiolactol 7 (as a mixture of anomers) with NaBH_4_ in methanol at reduced temperature. Finally, acid hydrolysis of the acetonide revealed the 2,3-diol (with partial hydrolysis of the 5-*O*-acetyl ester), and the per-acetylated sugar 9 was accessed upon treatment of the mixture of 8a and 8b with acetic anhydride and 4-(dimethylamino)pyridine (DMAP), which was a necessary acyl transfer catalyst to esterify the tertiary alcohol at C2 efficiently.

#### 2.1.2. Thionucleoside Analog Synthesis

With the per-acetylated thiosugar core (**9**) in hand, nucleoside analogs could be made via the stereoselective Vorbrüggen glycosylation of the nucleobases of interest. Vorbrüggen’s methodology generally makes use of a per-silylated heterocyclic nucleobase as glycosyl acceptor and a potent Lewis acid to activate the glycosyl donor for coupling to the nucleobase [31]. In the presence of a Lewis acid (e.g.*,* trimethylsilyl triflate or tin tetrachloride), the (thio)acetal at C1 of the glycosyl donor is thought to form an α-chalcogen-stabilized carbenium ion, which is then intramolecularly trapped by the adjacent ester at C2 to delocalize the positive charge around a newly formed 1,3-dioxolane ring fused to the bottom of the sugar. This anchimeric assistance provides the basis for the stereoselective “top-side” β-addition of the silylated glycosyl accepter nucleobase [32]. For its part, silylation of the nucleobase is reported to have two important effects—increased nucleophilicity and organic solubility of the nucleobase. The observation that trimethylsilyl (TMS) groups increase organic solubility is likely unsurprising given the lipophilicity of organosilicon compounds. However, the key benefit lies in the electron-releasing properties of silicon to impart increased nucleophilicity to the Lewis basic nitrogen atoms [33].

**Scheme 2:**
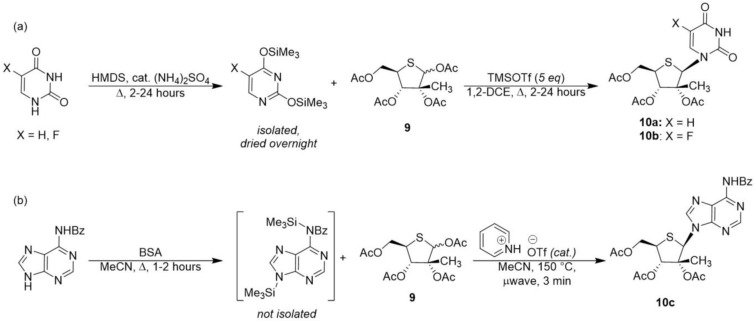
Two-step sequences used for glycosylation of nucleobases; (**a**) classical Vorbrüggen glycosylation sequence [31]; (**b**) modified microwave-assisted glycosylation using catalytic Brønsted acid [34].

Nucleobases were glycosylated using either one of two related yet distinct two-step protocols. The classical protocol (Scheme 2a) relies on pre-silylating the nucleobase by refluxing with neat hexamethyldisilazane (HMDS) in the presence of catalytic ammonium sulfate until the initially heterogeneous reaction mixture clarified [31]. This now persilylated nucleobase must be carefully concentrated to remove all traces of HMDS without exposure to moisture, as it easily hydrolyzes; failure to do so results in diminished yields of the glycosylated product. The concentrated crude persilylated nucleobase was then dissolved in 1,2-dichloroethane (DCE) and treated with **9** and TMS triflate (TMSOTf) under reflux conditions to afford the desired glycosylated product in good to excellent yields and diastereoselectivity. This protocol worked well for the natural and 5-fluorinated pyrimidine bases but struggled to produce the corresponding adenosine analog in an acceptable yield. Hence, an alternative procedure published by Sniady and co-workers [34] was employed, which utilizes *N,O*-bis(trimethylsilyl)acetamide (BSA) as silylating agent and pyridinium triflate instead as a Brønsted acid catalyst under microwave irradiation in acetonitrile solvent (Scheme 2b). Following these conditions, *N*-benzoyl-protected adenine was first refluxed with BSA in acetonitrile solvent for an hour before this crude reaction mixture was used directly to treat a solution of **9** and catalytic pyridinium triflate in acetonitrile. Microwave irradiation of the reaction mixture to 150 °C for only 3 min afforded the desired adenosine analog **10c** in 84% yield. These same microwave-assisted reaction conditions additionally afforded the 6-azauridine analog in 89% yield but did not prove generally reproducible with other pyrimidine nucleobases (e.g., uracil and cytosine).

Interestingly, the two procedures could be successfully hybridized to a sequential one-pot reaction, using BSA as silylating agent in DCE solvent, followed by direct treatment with the glycosyl donor (**9**) and TMSOTf and heating to reflux. The use of BSA as silylating agent represents an improvement in efficiency over HMDS as the presence of excess BSA and silylation byproducts do not appear to impact the glycosylation reaction. Indeed, following an even more streamlined procedure published by Haluszczak et al. [35], uracil could be combined with BSA and per-acetylated thiosugar **9** in 1,2-DCE and refluxed with TMSOTf to afford the glycosylated product in a single step with >90% yield, though diastereoselectivity was somewhat diminished (ca. 10:1 instead of 20:1). Once the glycosylated products were accessed, the free nucleoside analogs **11a–f** were prepared upon ammonolysis of the acetyl esters by heating with ammonia in methanol in a sealed tube. Notably, while guanine may have completed a structure–activity relationship (SAR) study of the natural nucleobases, it was considered strategically undesirable due to synthetic and toxicological concerns.

#### 2.1.3. Synthesis of the Phosphoramidate Monophosphate Prodrugs

Evaluation of the compounds in the cell-based replicon assay precludes the use of charged, highly polar NTPs as they fail to cross the cell membrane. The nucleoside itself could be considered, but antiviral activity then relies on cell permeability as well as all three intracellular kinases to anabolize the nucleoside to the NTP. Hence, the aryloxy phosphoramidate monophosphate prodrug utilized for sofosbuvir seemed like an obvious choice for testing the compounds (vide supra).

**Scheme 3:**
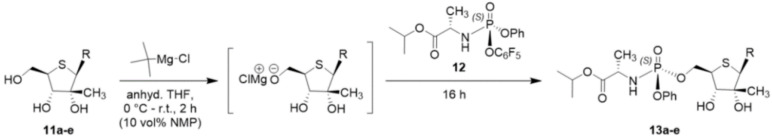
Traditional base-activated reaction utilized to access the desired phosphoramidate monophosphate prodrugs, except where R = cytosine **(11f**).

From the free nucleoside, the 5′-hydroxyl group is desired to react selectively in the presence of the secondary and tertiary alcohols at C3′ and C2′, respectively. Additionally, nucleosides with an exocyclic amine (i.e., cytidine and adenosine analogs) present an additional selectivity challenge. The use of the strong base tert-butylmagnesium chloride is one common approach to achieve this goal as the steric bulk of the tert-butyl group allows for a kinetic preference for the primary alcohol, while the oxophilicity of magnesium offers a thermodynamic bias over the amine moiety [36,37,38]. Accordingly, nucleoside phosphoramidates **13a–e** were prepared as shown in Scheme 3. Briefly, the nucleoside was dissolved in THF (with or without *N-*methylpyrrolidone co-solvent) and treated with the Grignard base to pre-form the magnesium alkoxide, followed by the addition of the chiral phosphorylating electrophile **12**, the synthesis of which is described elsewhere [39].

This procedure allowed access to nucleoside phosphoramidates **13a–e** in a serviceable yield. However, in the case where the nucleobase (R) was cytosine, the nucleoside appeared unreactive under these conditions. Cytidine analogs have been recognized before as problematic in prodrug synthesis, but Mayes and co-workers have made use of phenylboronic acid (PBA) to install a transient cyclic 2′,3′-boronate ester to assist in solubility and regioselectivity [40,41]. Encouragingly, performing the phosphoramidation reaction on the 2′,3′-PBA-protected natural cytidine model substrate afforded the desired cytidine phosphoramidate in 35% yield. While these conditions did not prove completely transferable to our 2′-β-methyl-4′-thiocytidine analog **11f**, we saw approximately 26% crude conversion of the nucleoside to the desired phosphoramidate.

**Scheme 4:**
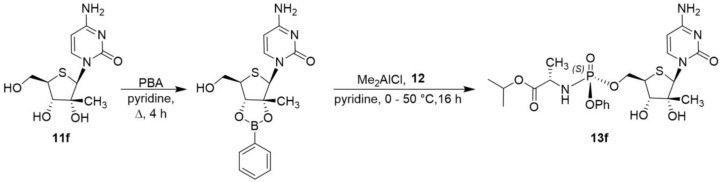
Alternative Lewis acid-activated phosphoramidation utilizing the transient 2′,3′-phenylboronic ester protecting group strategy to access the cytidine analog [41,42].

While this sequence was being explored, we became aware of an alternative solution to the ProTide synthesis problem offered by Simmons and co-workers wherein the authors report inverting the activation of the reagents to improve regioselectivity [42]. Rather than pre-activating the 5′-hydroxy nucleophile with a strong base, Simmons et al. report the use of sub-stoichiometric amounts of Lewis acid to lower the LUMO of the phosphoryl electrophile, allowing the reaction to proceed under general base-mediated conditions. Their optimized conditions utilize 0.5 equivalents of dimethylaluminum chloride with pyridine as base and solvent; in the cases of cytidine analogs, the authors report adding 5 equivalence of *N*,*N’*-dimethylpropyleneurea (DMPU) as co-solvent. Again, using natural cytidine as a model substrate, these reaction conditions were trialed without success. However, when the reaction conditions were applied to 2′,3′-PBA-protected cytidine, the desired phosphoramidate was isolated in nearly 40% yield, which was increased to over 60% with gentle heating. With the combination of the PBA transient protecting group and the aluminum activation of the electrophile providing the highest yield on the cytidine model substrate, this same combination of conditions was applied to the 4′-thiocytidine analog and gratifyingly afforded the desired phosphoramidate **13f** in a 37% yield (Scheme 4).

### 2.2. Antiviral Evaluation of Monophosphate Prodrugs

All synthesized phosphoramidates **13a–f** were evaluated for their anti-HCV activity in cultured Huh7 cells containing the HCV genotype 1b replicon as described elsewhere [43,44,45], and the data are shown in Table 1 compared to approved anti-HCV drug sofosbuvir as the positive control. The incorporation of non-natural pyrimidines (entries 2, 4, and 5) appeared deleterious to potency, while the natural pyrimidines were superior to the purine adenine, inhibiting replication at single-digit micromolar potencies with uracil proving the most potent (entry 1). None of the compounds showed marked cytotoxicity (TC_50_) up to the maximum dose tested (200 μM). Additionally, a single-dose screen of the compounds at 5 μM showed between 0–12% reduction in cytopathic effect (CPE) against Huh7 cells infected with the Dengue virus DENV2_New Guinea_ and up to 20% against Zika_PRVABC59_, whereas sofosbuvir performed well against both (100% CPE reduction at 5 μM against DENV2 and an antiviral efficacy (EC_50_) of 1.63 μM against Zika).

**Figure 3:**
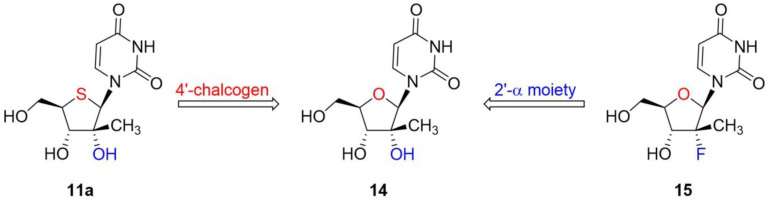
Comparison of the structural features of the most potent nucleoside of this work (**11a**) to sofosbuvir (**15**), with the bridging structure of 2′-β-methyluridine (**14**).

Noting that sofosbuvir utilizes the same monophosphate prodrug moiety as our analogs, a comparison of the conjugated nucleosides can reasonably be made (Figure 3). Even the most potent analog (entry 1) suffered a 42-fold drop in potency compared to sofosbuvir (entry 7) which could only be accounted for by the changes in the nucleoside. Two important structural features should be considered when comparing the nucleoside of our most potent compound (i.e., the 2′-β-methyl-4′-thiouridine, **11a**) to the 2′-deoxy-2′-fluoro-2′-β-methyluridine nucleoside of sofosbuvir (**15**): the exchange of the 4′-chalcogen (red), as well as the 2′-α-hydroxy versus fluorine moiety (blue). Since the nucleobase and 5′-*O*-phosphoramidate are the same, is the observed loss of potency related to the 2′-substituent or the sugar ring heteroatom (or some combination of the two)? The molecule that could bridge the gap would therefore be the same aryloxy phosphoramidate prodrug of 2′-β-methyluridine (**14**), bearing a 2′-α-hydroxy group and the natural tetrahydrofuran sugar ring. Butora and colleagues reported an EC_50_ of 0.03 μM for this prodrug in the same HCV genotype 1b replicon assay, in agreement with what we have observed for the P-racemate of pro-**14** [46,47]. These data indicate a 70-fold drop in HCV potency when the natural tetrahydrofuran ring is replaced with tetrahydrothiophene. Moreover, Bernatchez and co-workers recently published an EC_50_ of 1 μM for the same aryloxy phosphoramidate prodrug of **14** against Zika, >5 fold more potent than our 4′-thio congener; however, their results were in neural stem cells, and anti-Zika potency has been suggested to be cell line-dependent [48]. For example, in that same study Bernatchez et al. recorded an EC_50_ of 35 μM for sofosbuvir in neural stem cells, whereas we observed a 1.63 μM EC_50_ in Huh7 cells. It is likely that this discrepancy can be attributed to differential metabolism of the prodrug in different cells.

It is not immediately obvious why this subtle structural change in the nucleoside would lead to such a pronounced drop in potency. The incorporation of sulfur is sure to have stereoelectronic effects resulting from the differences in polarizability of C–S bonds compared to C–O bonds. Relatedly, carbocyclic nucleosides that eliminate such polarizability are not well demonstrated in the literature, but a few examples exist with similarly poor results. Liu and co-workers synthesized and tested the A and C carbocyclic analogs of 2′-fluoro-2′-*C*-methylnucleosides showing inferior anti-HCV activity compared to the 4′-oxo congener (>50 and 18.2 μM versus 3.7 μM) [49]. Several groups have investigated base-modified analogs of the carbocyclic nucleoside natural product neplanocin A, with the only notable potency coming from the 7-deaza analog (ca. 1.7 μM) [50,51,52]. Note that, in all of these cases, the nucleoside was tested in the cellular assay, leaving one to wonder how a corresponding monophosphate prodrug might perform.

In addition to electronic effects, differences in bond lengths and angles in the sugar ring can be expected to result from the O-to-S substitution, likely affecting the conformational dynamics of the system. Studies are currently underway in our lab to better understand how these conformational changes might affect anti-HCV activity, noting the pioneering work of Victor Marquez’s lab on other viruses [53,54] and Martínez-Montero and colleagues on HCV in particular [55]. Our hope is that a clearer picture of the effect of conformation on antiviral potency would become a general consideration for the design of nucleoside antivirals, leading to more potent molecules to combat diseases caused by other RNA viruses such as Dengue, Zika and SARS-CoV-2 which currently have no antiviral therapy.

## 3. Materials and Methods

### 3.1. Synthetic Chemistry

#### 3.1.1. General Considerations

All commercially available starting materials were purchased and used as provided unless otherwise specified. When anhydrous conditions are indicated, anhydrous solvents were used from commercial suppliers. Automated flash column chromatography was performed using a Teledyne ISCO (Lincoln, NE, USA) CombiFlash NextGen system with silica gel-packed columns (RediSep^®^ R_f_). Analytical thin-layer chromatography (TLC, commercially available from VWR (Radnor, PA, USA)) was carried out on Merck aluminum-supported silica gel plates (thickness: 200 mm) with fluorescent indicator (F-254). Visualization of compounds on TLC plates was accomplished with UV light (254 nm) and/or with phosphomolybdic acid, ninhydrin, ceric ammonium molybdate, or potassium permanganate staining (Sigma-Aldrich, St. Louis, MO, USA). NMR spectra (^1^H, ^13^C, ^19^F and ^31^P) were obtained in the Emory University NMR Research Center, directed by Dr. Shaoxiong Wu and Dr. Bing Wang, using either a Bruker INFINITY II 600 MHz spectrometer with cryogenic probe (funded by National Science Foundation grant CHE-1531620), a Varian INOVA 500 MHz spectrometer, a Varian INOVA 400 MHz spectrometer, or a Varian VNMR 400 MHz spectrometer. NMR samples were prepared and processed in deuterated chloroform (CDCl_3_), deuterated MeOH (CD_3_OD), deuterated dimethyl sulfoxide (*d*_6_-DMSO), or deuterated acetone (*d_6_*-acetone) using the residual solvent peak (CDCl_3_: ^1^H = 7.26 ppm, ^13^C = 77.16 ppm; CD_3_OD: ^1^H = 3.31 ppm, ^13^C = 49.00 ppm; *d_6_*-DMSO: ^1^H = 2.50 ppm, ^13^C = 39.52 ppm; *d_6_*-acetone: ^1^H = 2.05 ppm, ^13^C = 29.84 ppm) as an internal reference. NMR data are reported to include chemical shifts (δ) reported in ppm, multiplicities indicated as s (singlet), d (doublet), dd (doubled doublet), t (triplet), td (tripled doublet), q (quartet), ddd (doubled doubled doublet), m (multiplet), or br s (broad singlet), coupling constants (*J*) reported in Hz, and integration normalized to 1 atom. High-resolution mass spectrometry (HRMS) was performed by the Emory University Mass Spectrometry Center, directed by Dr. Fred Strobel. Liquid chromatography–mass spectrometry (LC–MS) was performed on an Agilent 1200 HPLC equipped with a 6120 Quadrupole mass spectrometer (ESI–API) eluting at a rate of 1.00 mL/min with mixtures of HPLC grade MeOH and water or acetonitrile and water (all spiked with 0.1% formic acid) through an analytical, reverse-phase Agilent C18 XDB eclipse column (50 mm × 4.6 mm, 3.5 μM). LC–MS samples were prepared in a solution of 75:25 MeOH/water or 50:50 acetonitrile/water (spiked with 0.1% formic acid), and ultraviolet activity was monitored at 254 nm. Final compound purity was assessed to be ≥95% pure using ^1^H NMR and LC–MS.

#### 3.1.2. Synthesis of the Per-Acetylated Thiosugar Core

(3a*R*,6*R*,6a*R*)-6-(hydroxymethyl)-2,2,3a-trimethyldihydrofuro [3,4-d][1,3]dioxol-4(3a*H*)-one (**1.1**):

A 500-mL round-bottomed flask equipped with reflux condenser and magnetic stir bar was charged with (3*R*,4*R*,5*R*)-3,4-dihydroxy-5-(hydroxymethyl)-3-methyldihydrofuran-2(3*H*)-one (5 g, 30.8 mmol) and acetone (100 mL, 1363 mmol) to give a tan solution. Sulfuric acid (0.25 mL, 4.69 mmol) was added dropwise, and the solution was heated to reflux with stirring. After 4 h, the reaction was cooled with an ice bath and carefully neutralized with solid NaHCO_3_ before the reaction mixture was filtered through Celite^®^. The crude filtrate was concentrated to afford an off-white solid (5.94 g, 95% yield); NMR spectra were consistent with that previously reported [56], see Supplementary Materials.

((3ar,4*r*,6ar)-2,2,6a-trimethyl-6-oxotetrahydrofuro[3,4-d][1,3]dioxol-4-yl)methyl methanesulfonate (**2**):

A 50-mL round-bottomed flask with magnetic stir bar was charged with acetonide **1.1** (5.74 g, 28.4 mmol) and anhydrous pyridine (4.7 mL) under argon to give a colorless solution. The solution was cooled to 0 °C with an ice bath before methanesulfonyl chloride (3.3 mL, 42.6 mmol) was slowly added dropwise with stirring. The reaction was left to stir at 0 °C for approximately 30 min before the ice bath was removed, and the reaction warmed to ambient temperature for another 30 min. Approximately 15 mL of deionized H_2_O was added, and the product was extracted with dichloromethane (DCM). The organic layer was washed with 1M HCl followed by saturated NaHCO_3_ solution. The organic phase was dried over Na_2_SO_4_, filtered and concentrated to afford a white solid (7.88 g, 99% yield); NMR spectra were consistent with that previously reported [56].

(3a*R*,6*S*,6a*R*)-6-(hydroxymethyl)-2,2,3a-trimethyldihydrofuro[3,4-d][1,3]dioxol-4(3aH)-one (**3**):

A 500-mL round-bottomed flask with magnetic stir bar was charged with mesylate **2** (3.2 g, 11.42 mmol) and 1,4-dioxane (70.5 mL) to give a tan solution. In a separate beaker, solid KOH (1.92 g, 34.2 mmol) was dissolved in deionized water (55.0 mL), and after the heat of dissolution had dissipated the resulting solution was added to the reaction flask with stirring, noting an exotherm. The reaction was allowed to stir at ambient temperature for approximately 4 h. The reaction was acidified with 3 N HCl to pH 1 with stirring, and the crude product was concentrated to dryness. The remaining white solid was triturated with ethyl acetate, and the insoluble material was filtered and further washed with ethyl acetate. The filtrate was concentrated to afford a tan oil (2.29 g, 99% yield); NMR spectra were consistent with those previously reported [56].

((3a*R*,4*S*,6a*R*)-2,2,6a-trimethyl-6-oxotetrahydrofuro[3,4-d][1,3]dioxol-4-yl)methyl 4-methylbenzenesulfonate (**3.1**):

A flame-dried 250-mL round-bottom flask with stir bar was charged with argon, lactone **3** (2 g, 9.89 mmol) and DCM (30 mL) to give a colorless solution. The solution was chilled to 0 °C before *N,N*-dimethylpyridin-4-amine (0.06 g, 0.495 mmol) and *N*-ethyl-*N*-isopropylpropan-2-amine (3.5 mL, 19.78 mmol) were added with stirring to give a slightly yellow solution. Lastly, 4-methylbenzene-1-sulfonyl chloride (2.07 g, 10.88 mmol) was slowly added with stirring. The reaction was stirred for 30 min before the ice bath was removed and was then left to stir overnight while warming to ambient temperature. The next morning, TLC (3:2 hexanes:ethyl acetate) indicated the conversion of the starting material to a new UV-active spot, so the reaction was quenched by the addition of cold 1 N HCl. The product was twice extracted with DCM, and the organic extracts were combined and washed with saturated NaHCO_3_ solution before being dried over sodium sulfate, filtered and concentrated to afford a brown oil (3.4 g, 93% yield); NMR spectra were consistent with that previously reported [57].

(4*R*,5*R*)-methyl 2,2,4-trimethyl-5-((*S*)-oxiran-2-yl)-1,3-dioxolane-4-carboxylate (**4**):

An oven-dried 50-mL round-bottom flask with magnetic stir bar was charged with argon and tosylate **3.1** (3.04 g, 8.53 mmol). The apparatus was chilled with an ice bath before NaOMe (18.8 mL, 9.38 mmol) was added as a solution in methanol dropwise with stirring to give a colorless solution. The reaction was stirred at 0 °C for 1 h before the ice bath was removed, allowing the reaction to warm to ambient temperature while stirring overnight. The reaction was quenched with 10 mL of saturated NH_4_Cl solution and diluted with 50 mL of deionized water before the product was extracted with ethyl acetate. The organic layers were combined, dried over Na_2_SO_4_, filtered and concentrated to afford the crude product, a pale yellow semisolid (1.70 g, 92% yield); ^1^H NMR (400 MHz, CDCl_3_) δ 3.76 (s, 3H), 3.58 (d, *J* = 6.0 Hz, 1H), 3.00 (ddd, *J* = 6.0, 4.2, 2.7 Hz, 1H), 2.85 (dd, *J* = 4.8, 4.2 Hz, 1H), 2.71 (dd, *J* = 4.9, 2.7 Hz, 1H), 1.58 (s, 3H), 1.55 (s, 3H), 1.40 (s, 3H) ppm; ^13^C NMR (101 MHz, CDCl_3_) δ 172.7, 111.1, 85.5, 82.3, 52.5, 49.7, 43.5, 26.6, 23.4 ppm.

(4*R*,5*S*)-methyl 2,2,4-Trimethyl-5-((*S*)-thiiran-2-yl)-1,3-dioxolane-4-carboxylate (**5**):

A 25-mL round-bottom flask with magnetic stir bar was charged with epoxide **4** (0.75 g, 3.47 mmol) and anhydrous DMF (1.88 mL), and the colorless solution was heated to 60 °C. Freshly recrystallized thiourea (0.46 g, 6.00 mmol) was added, and the reaction stirred for 2 h before another half equivalent of thiourea (0.132 g, 1.734 mmol) was added. Monitoring by TLC indicated complete conversion of starting material in 4.5 h. The reaction was quenched by the addition of water and diluted with 25 mL of brine solution before the product was extracted with ethyl acetate. The organic extracts were combined, dried over Na_2_SO_4_, filtered and concentrated to afford 1.06 g of crude product. The product mixture was brought up in DCM and filtered over a plug of silica gel, eluting with a 25% ethyl acetate solution in hexanes. The filtrate fractions of interest were concentrated to afford the desired product as a white powder (0.41 g, 51% yield); ^1^H NMR (400 MHz, CDCl_3_) δ 3.78 (s, 3H), 3.35 (d, *J* = 8.4 Hz, 1H), 2.77 (ddd, *J* = 8.2, 5.9, 4.9 Hz, 1H), 2.53 (dd, *J* = 6.0, 1.5 Hz, 1H), 2.26 (dd, *J* = 5.0, 1.4 Hz, 1H), 1.61 (s, 3H), 1.57 (s, 4H), 1.39 (s, 3H) ppm; ^13^C NMR (101 MHz, CDCl_3_) δ 172.8, 110.7, 88.9, 84.2, 52.5, 30.2, 26.9, 26.8, 23.8, 22.9 ppm; HRMS (NSI) m/z: [M + H]^+^ Calcd for C_10_H_17_O_4_S 233.0842; Found 233.0844.

((3a*S*,4*R*,6a*R*)-2,2,6a-trimethyl-6-oxotetrahydrothieno[3,4-d][1,3]dioxol-4-yl)methyl acetate (**6**):

An oven-dried 250-mL round-bottom flask equipped with stir bar and reflux condenser was charged with thiirane **5** (0.32 g, 1.378 mmol) and sodium acetate (11.75 g, 143 mmol) under argon. Acetic acid (23 mL) was added followed by acetic anhydride (1.3 mL, 13.78 mmol) to give a moist powder. The reaction was heated to reflux, dissolving the mixture, and allowed to stir for 21 h with monitoring by TLC. When the reaction was pulled from heat, the solution solidified. The solid was dissolved by the addition of DCM and careful addition of saturated NaHCO_3_ solution. The product was extracted with DCM, and the organic extracts were combined, dried over Na_2_SO4, filtered and concentrated to afford 0.3 g of a brown oil, which was purified via silica gel flash column chromatography (eluted with a 0–15% gradient of ethyl acetate in hexanes). The fractions of interest were combined to afford the desired five-membered product (0.23 g, 65% yield); ^1^H NMR (400 MHz, CDCl_3_) δ 4.33 (d, *J* = 0.7 Hz, 1H), 4.31 (d, *J* = 6.3 Hz, 1H), 4.31 (d, *J* = 7.0 Hz, 1H), 4.01 (ddd, *J* = 7.0, 6.3, 0.7 Hz, 1H), 2.12 (s, 3H), 1.54 (s, 3H), 1.44 (d, *J* = 0.8 Hz, 3H), 1.40 (d, *J* = 0.8 Hz, 3H) ppm; ^13^C NMR (101 MHz, CDCl_3_) δ 206.1, 170.4, 111.9, 89.9, 82.9, 64.7, 46.2, 27.5, 26.7, 20.9, 20.3 ppm; HRMS (NSI) m/z: [M + Na]^+^ Calcd for C_11_H_16_O_5_SNa 283.0611; Found 283.0615.

((3a*S*,4*R*,6a*R*)-6-hydroxy-2,2,6a-trimethyltetrahydrothieno[3,4-d][1,3]dioxol-4-yl)methyl acetate (**7**):

A 250-mL round-bottom flask with magnetic stir bar was charged with thiolactone **6** (0.606 g, 2.328 mmol) and methanol (36 mL) under argon to give a colorless solution. The solution was chilled to −15 °C before NaBH_4_ (0.176 g, 4.66 mmol) was added under argon with stirring. After one hour, another portion of NaBH_4_ (0.176 g, 4.66 mmol) was added; two additional portions of the reductant (0.176 g, 4.66 mmol each) were added on the hour for a total of 8 equivs, and the reaction was allowed to stir at −15 °C with monitoring by LC–MS. After the starting material was consumed, the reaction was quenched at −15 °C by the addition of 5 wt% aqueous citric acid solution. The product mixture was then extracted with DCM, and the organic extracts were combined, dried over Na_2_SO_4_, filtered and concentrated to afford a lightly colored oil as the crude product, a 7:3 mixture of anomers (0.56 g, 96% yield); ^1^H NMR (400 MHz, CDCl_3_) δ 5.11 (d, *J* = 4.1 Hz, 0.7H, major), 5.10 (d, *J* = 9.3 Hz, 0.3H, minor), 4.53 (s, 0.7H, major), 4.40 (d, *J* = 2.0 Hz, 0.3H, minor), 4.35 (d, *J* = 5.7 Hz, 0.3H, minor), 4.33 (d, *J* = 5.7 Hz, 0.3H, minor), 4.20–4.13 (m, 1.4H), 3.55–3.51 (m, 0.3H, minor), 3.50–3.46 (m, 0.7H, major), 3.25 (d, *J* = 9.3 Hz, 0.3H, minor), 2.88 (d, *J* = 4.3 Hz, 0.7H, major), 2.09 (s, 2.1H, major), 2.08 (s, 0.9H, minor), 1.565 (s, 0.9H, minor), 1.560 (s, 2.1H, major), 1.54 (s, 0.9H, minor), 1.49 (s, 2.1H, major), 1.44 (s, 0.9H, minor), 1.40 (s, 2.1H, major) ppm; ^13^C NMR (101 MHz, CDCl_3_) δ 170.9 (major), 170.6 (minor), 113.1 (minor), 111.7 (major), 96.7 (major), 91.9 (major), 91.3 (minor), 90.3 (minor), 88.8 (major), 86.1 (minor), 66.5 (major), 65.7 (minor), 53.9 (major), 48.8 (minor), 28.1 (2C, major), 27.5 (minor), 27.1 (minor), 24.4 (minor), 22.4 (major), 21.1 (major), 21.0 (minor) ppm; HRMS (NSI) *m*/*z*: [M + Na]^+^ Calcd for C_11_H_18_O_5_SNa 285.0767; Found 285.0770.

((2*R*,3*S*,4*R*)-3,4,5-trihydroxy-4-methyltetrahydrothiophen-2-yl)methyl acetate (**8a**):

A 250-mL round-bottom flask with magnetic stir bar was charged with thiolactol **7** (0.68 g, 2.59 mmol) and 20 mL of an ice-cold 40 vol% aqueous solution of trifluoroacetic acid. The reaction was stirred at ambient temperature for 3 h when TLC (1:4 Hex:EA) showed conversion of starting material to two more polar products. The reaction mixture was concentrated via rotary evaporation followed by azeotropic removal of residual acid via co-evaporation with toluene followed by methanol then ethanol. The resulting oil was purified via silica gel flash column chromatography (eluted with a gradient of 0–15% methanol in ethyl acetate). The fractions of interest were combined for the desired 1,2,3-glycol ([M + Na]^+^ = 245.0) as a 3:2 mixture of anomers (later fractions were also collected for the 5-deacetylated product (**8b**, [M + Na]^+^ = 203.0)) (0.52 g, 90% yield); ^1^H NMR (399 MHz, CD_3_OD) δ 5.20 (s, 0.4H, minor), 4.82 (s, 0.6H, major), 4.61–4.47 (m, 1H, both), 4.12 (dd, *J* = 11.0, 8.2 Hz, 0.6H, major), 4.02 (dd, *J* = 11.2, 7.9 Hz, 0.4H, minor), 3.80–3.70 (m, 1H, both), 3.53–3.43 (m, 1H, both), 2.05 (s, 1.8H, major), 2.04 (s, 1.2H, minor), 1.33 (s, 1.8H, major), 1.28 (s, 1.2H, minor) ppm; ^13^C NMR (100 MHz, CD_3_OD) δ 172.7 (major), 172.6 (minor), 85.6 (major), 85.3 (minor), 82.8 (major), 82.7 (minor), 79.5 (minor), 79.3 (major), 68.3 (major), 67.2 (minor), 49.8 (minor), 48.7 (major), 22.3 (both), 20.9 (major), 20.7 (minor) ppm; HRMS (NSI) *m*/*z*: [M + Na]^+^ Calcd for C_8_H_14_O_5_SNa 245.0454; Found 245.0455.

(3*R*,4*S*,5*R*)-5-(acetoxymethyl)-3-methyltetrahydrothiophene-2,3,4-triyl triacetate (**9**):

A 50-mL round-bottom flask with magnetic stir bar was charged with a mixture of thioribofuranose derivatives **8a** and **8b** (0.874 g, 3.93 mmol) which was dried in vacuo overnight. Solid anhydrous NaOAc (0.419 g, 5.11 mmol) was added under argon followed by acetic anhydride (3.7 mL, 39.3 mmol) to give a colorless slurry; finally *N,N*-dimethylpyridin-4-amine (48 mg, 0.393 mmol) was added. The reaction was heated to 80 °C and allowed to stir approximately 4 h with monitoring by TLC for consumption of starting material. The reaction was quenched by careful addition of saturated aqueous sodium bicarbonate solution. The product was extracted with DCM; the organic layers were combined, dried over Na_2_SO_4_, filtered and concentrated to a thin yellow-brown oil. The oil was brought up in DCM and purified via silica gel flash column chromatography (eluted with a 0–30% gradient of ethyl acetate in hexanes). The fractions of interest were combined and concentrated to afford the desired product as a 2:1 mixture of anomers (1.29 g, 94% yield); ^1^H NMR (400 MHz, CDCl_3_) δ 6.44 (s, 0.7H, major), 6.28 (s, 0.3H, minor), 5.35 (d, *J* = 3.2 Hz, 0.3H, minor), 5.34 (d, *J* = 9.3 Hz, 0.7H, major), 4.36–4.38 (m, 1.4H), 4.17–4.09 (m, 1.4H), 3.80–3.72 (m, 1.4H), 2.18 (s, 2H, major), 2.11 (s, 2H, major), 2.10 (s, 3H, both), 2.083 (s, 1H, minor), 2.078 (s, 1H, minor), 2.04 (s, 2H, major), 2.03 (s, 1H, minor), 1.74 (s, 1H, minor), 1.58 (s, 2H, major) ppm; ^13^C NMR (151 MHz, CDCl_3_) δ 170.5, 170.2, 169.8, 169.3, 88.0, 78.8, 78.0, 65.5, 44.8, 22.1, 21.3, 21.0, 20.8, 17.0 ppm; HRMS (ESI) m/z: [M + Na]^+^ Calcd for C_14_H_20_O_8_SNa 371.0771; Found 371.0768.

#### 3.1.3. Synthesis of the 4′-Thionucleosides

General Procedure for Nucleoside Synthesis Using HMDS and TMSOTf

The nucleobase of interest (5–6 eq) was suspended in anhydrous hexamethyldisilazane (HMDS, 0.5 M in nucleobase) with catalytic ammonium sulfate (7.5 mol% relative to nucleobase) and heated to reflux under argon until the reaction clarified (1–2 h). The reaction mixture was then carefully concentrated to dryness and placed under high vacuum overnight, after which time the silylated intermediate was brought up in 1,2-DCE (1 M in nucleobase) under argon and treated with (3*R*,4*S*,5*R*)-5-(acetoxymethyl)-3-methyltetrahydrothiophene-2,3,4-triyl triacetate **9** (1 eq) and TMSOTf, (5.5 eq). The reaction was heated to 60 °C and left to stir until starting material was consumed by TLC. The reaction was allowed to cool to ambient temperature before being quenched with a saturated aqueous solution of NaHCO_3_, and the product was extracted with DCM. The organic layer was dried over Na_2_SO_4_, filtered, concentrated and purified via silica gel flash column chromatography (eluted with 0–10% methanol gradient in DCM) to afford the glycosylated product, which was subsequently deprotected by stirring with a 7 N solution of ammonia in methanol overnight in a sealed vessel. The next day, the volatiles were removed, and the residue was purified via silica gel flash column chromatography (eluted with 0–15% methanol gradient in DCM) to afford the desired 4′-thionucleoside analog.

1-((2*R*,3*R*,4*S*,5*R*)-3,4-dihydroxy-5-(hydroxymethyl)-3-methyltetrahydrothiophen-2-yl)pyrimidine-2,4(1*H*,3*H*)-dione (**11a**):

Following the general procedure, uracil (0.78 g, 6.95 mmol) was glycosylated with **9** (484 mg, 1.39 mmol) and subsequently deprotected to afford the desired product (245 mg, 64% yield over two steps); ^1^H NMR (399 MHz, CD_3_OD) δ 8.36 (d, *J* = 8.2 Hz, 1H), 5.95 (s, 1H), 5.75 (d, *J* = 8.1 Hz, 1H), 3.99–3.90 (m, 2H), 3.73 (d, *J* = 9.5 Hz, 1H), 3.48 (ddd, *J* = 9.4, 4.7, 2.9 Hz, 1H), 1.17 (s, 3H) ppm; ^13^C NMR (100 MHz, CD_3_OD) δ 165.4, 152.8, 143.8, 102.6, 83.2, 77.3, 68.0, 61.6, 53.6, 21.0 ppm; HRMS (NSI) *m*/*z*: [M + Na]^+^ Calcd for C_10_H_14_O_5_N_2_SNa 297.0516; Found 297.0508.

1-((2*R*,3*R*,4*S*,5*R*)-3,4-dihydroxy-5-(hydroxymethyl)-3-methyltetrahydrothiophen-2-yl)-5-fluoropyrimidine-2,4(1*H*,3*H*)-dione (**11b**):

Following the general procedure, 5-fluorouracil (195 mg, 1.50 mmol) was glycosylated with **9** (80 mg, 0.23 mmol) and subsequently deprotected to afford the desired product (50 mg, 75% yield over two steps); ^1^H NMR (399 MHz, CD_3_OD) δ 8.72 (d, ^3^*J*_H–F_ = 7.4 Hz, 1H), 5.90 (d, ^5^*J*_H–F_ = 1.5 Hz, 1H), 3.98 (dd, *J* = 11.9, 3.6 Hz, 1H), 3.90 (dd, *J* = 11.8, 2.5 Hz, 1H), 3.79 (d, *J* = 9.4 Hz, 1H), 3.44 (ddd, *J* = 9.4, 3.5, 2.5 Hz, 1H), 1.20 (s, 3H) ppm; ^13^C NMR (100 MHz, CD_3_OD) δ 159.3 (d, ^2^*J*_C–F_ = 26.1 Hz), 151.7, 141.2 (d, ^1^*J*_C–F_ = 233.6 Hz), 127.8 (d, ^2^*J*_C–F_ = 35.8 Hz), 83.4, 76.7, 68.9, 60.6, 53.4, 20.7 ppm; ^19^F NMR (376 MHz, CD_3_OD) δ-167.3 (d, ^3^*J*_F–H_ = 6.9 Hz) ppm; HRMS (NSI) *m*/*z*: [M – H]^-^ Calcd for C_10_H_12_O_5_N_2_FS 291.0456; Found 291.0458.

4-amino-1-((2*R*,3*R*,4*S*,5*R*)-3,4-dihydroxy-5-(hydroxymethyl)-3-methyltetrahydrothiophen-2-yl)-5-fluoropyrimidin-2(1*H*)-one (**11e**):

Following the general procedure, *N^4^*-benzoyl-5-fluorocytosine (380 mg, 1.63 mmol) was glycosylated with **9** (97 mg, 0.28 mmol) and subsequently deprotected to afford the desired product (67 mg, 83% yield over two steps); ^1^H NMR (399 MHz, CD_3_OD) δ 8.65 (d, ^3^*J*_H–F_ = 7.3 Hz, 1H), 6.02 (d, ^5^*J*_H–F_ = 1.9 Hz, 1H), 3.98 (dd, *J* = 11.9, 3.6 Hz, 1H), 3.91 (dd, *J* = 11.9, 2.5 Hz, 1H), 3.78 (d, *J* = 9.4 Hz, 1H), 3.45 (ddd, *J* = 9.4, 3.6, 2.6 Hz, 1H), 1.15 (s, 3H) ppm; ^13^C NMR (100 MHz, CD_3_OD) δ 159.2 (d, ^2^*J*_C–F_ = 14.0 Hz), 157.4, 137.9 (d, ^1^*J*_C–F_ = 243.7 Hz), 128.4 (d, ^2^*J*_C–F_ = 33.1 Hz), 83.4, 76.7, 69.3, 60.8, 53.3, 20.7 ppm; ^19^F NMR (376 MHz, CD_3_OD) δ-166.8 (d, ^3^*J*_F–H_ = 7.2 Hz) ppm; HRMS (NSI) *m*/*z*: [M – H]^−^ Calcd for C_10_H_13_O_4_N_3_FS 290.0616; Found 290.0619.

4-amino-1-((2*R*,3*R*,4*S*,5*R*)-3,4-dihydroxy-5-(hydroxymethyl)-3-methyltetrahydrothiophen-2-yl)pyrimidin-2(1*H*)-one (**11f**):

Following the general procedure, *N^4^*-acetylcytosine (0.5 g, 3.27 mmol) was glycosylated with **9** (256 mg, 0.74 mmol) and subsequently deprotected to afford the desired product (104 mg, 52% yield over two steps); ^1^H NMR (400 MHz, CD_3_OD) δ 8.36 (d, *J* = 7.6 Hz, 1H), 6.11 (s, 1H), 5.93 (d, *J* = 7.5 Hz, 1H), 3.97–3.92 (m, 2H), 3.72 (d, *J* = 9.5 Hz, 1H), 3.47 (dt, *J* = 9.5, 3.6 Hz, 2H), 1.12 (s, 3H) ppm; ^13^C NMR (101 MHz, CD_3_OD) δ 167.1, 159.0, 144.4, 96.2, 83.4, 77.2, 68.6, 61.5, 53.3, 20.7 ppm; HRMS (NSI) *m*/*z*: [M + H]^+^ Calcd for C_10_H_16_O_4_N_3_S 274.0856; Found 274.0855.

General procedure for Nucleoside Synthesis via the Microwave-Assisted Glycosylation of Nucleobases with BSA and Pyridinium Triflate.

The nucleobase of interest (1.1 eq) was suspended in anhydrous acetonitrile (0.2 M in nucleobase) under argon and treated with *N,O*-bis(trimethylsilyl)acetamide (2.2 eq relative to nucleobase), and the reaction was heated to 75 °C for 1–2 h. Meanwhile, an oven-dried microwave vial was charged with argon, catalytic pyridinium triflate (5 mol% relative to glycosyl donor), and (3*R*,4*S*,5*R*)-5-(acetoxymethyl)-3-methyltetrahydrothiophene-2,3,4-triyl triacetate **9** (1 eq) as a 0.4 M solution in anhydrous acetonitrile. After cooling to ambient temperature, the crude silylated intermediate was delivered to the microwave vial under argon. The suspension stirred briefly before being irradiated to 150 °C until starting material was consumed by TLC. The reaction mixture was concentrated to dryness and purified via silica gel flash column chromatography (eluted with 0–10% methanol gradient in DCM) to afford the glycosylated product, which was subsequently deprotected by stirring with a 7 N solution of ammonia in methanol overnight in a sealed vessel. The next day, the volatiles were removed, and the residue was purified via silica gel flash column chromatography (eluted with 0–15% methanol in DCM) to afford the desired 4′-thionucleoside analog.

(2*R*,3*R*,4*S*,5*R*)-2-(6-amino-9*H*-purin-9-yl)-5-(hydroxymethyl)-3-methyltetrahydrothiophene-3,4-diol (**11c**):

Following the microwave-assisted general procedure, *N*^6^-benzoyladenine (52 mg, 0.22 mmol) was glycosylated with **9** (70 mg, 0.2 mmol) and subsequently deprotected to afford the desired product (42 mg, 71% yield over two steps); ^1^H NMR (400 MHz, CD_3_OD) δ 8.68 (s, 1H), 8.22 (s, 1H), 5.83 (s, 1H), 4.10 (d, *J* = 9.4 Hz, 1H), 4.05–3.97 (m, 2H), 3.61 (ddd, *J* = 9.4, 4.2, 3.2 Hz, 1H), 0.92 (s, 3H) ppm; ^13^C NMR (101 MHz, CD_3_OD) δ 157.4, 153.9, 151.1, 142.2, 83.4, 77.1, 66.4, 61.8, 53.5, 21.0 ppm; HRMS (NSI) *m*/*z*: [M + H]^+^ Calcd for C_11_H_16_O_3_N_5_S 298.0968; Found 298.0969.

2-((2*R*,3*R*,4*S*,5*R*)-3,4-dihydroxy-5-(hydroxymethyl)-3-methyltetrahydrothiophen-2-yl)-1,2,4-triazine-3,5(2*H*,4*H*)-dione (**11d**):

Following the microwave-assisted general procedure, 6-azauracil (18 mg, 0.158 mmol) was glycosylated with **9** (42 mg, 0.12 mmol) and subsequently deprotected to afford the desired product (20 mg, 65% yield over two steps); ^1^H NMR (400 MHz, CD_3_OD) δ 7.52 (s, 1H), 6.01 (s, 1H), 4.07 (dd, *J* = 10.9, 3.7 Hz, 1H), 3.98 (d, *J* = 8.9 Hz, 1H), 3.74 (dd, *J* = 10.9, 8.6 Hz, 1H), 3.51 (td, *J* = 8.7, 3.7 Hz, 1H), 1.19 (s, 3H) ppm; ^13^C NMR (126 MHz, CD_3_OD) δ 158.3, 150.3, 137.3, 83.4, 79.4, 69.8, 65.8, 53.9, 20.7 ppm; HRMS (NSI) *m*/*z*: [M – H]^–^ Calcd for C_9_H_12_O_5_N_3_S 274.0492; Found 274.0505.

#### 3.1.4. Synthesis of the Phosphoramidate Monophosphate Prodrugs

General Procedure for the Phosphoramidate Coupling Reaction

The 4′-thionucleoside of interest (1 eq) was dried in vacuo overnight and then dissolved in anhydrous THF (0.2 M) with or without 10 vol% *N*-methylpyrrolidone co-solvent, depending on solubility. The solution was cooled to 0 °C before being dropwise treated with tert-butylmagnesium chloride (1.2–2 eq) as a 1 M solution in THF under argon, resulting in a thick white slurry. The ice bath was removed, and the deprotonation proceeded while slowly warming to ambient temperature over 2–3 h. The intermediate magnesium alkoxide was then dropwise treated with (*S*)-isopropyl 2-(((*S*)-(perfluorophenoxy)(phenoxy)phosphoryl)amino)propanoate **12** [39] (1–2 eq) as a 0.4 M solution in anhydrous THF, and the resulting slurry stirred at ambient temperature overnight. The next day, the reaction was quenched by the addition of saturated aqueous NH_4_Cl solution, and the product was extracted with DCM. The organic layers were combined, dried over Na_2_SO_4_, filtered, concentrated and purified via silica gel flash column chromatography (eluting with 0–15% methanol in DCM) to afford the desired nucleoside phosphoramidate.

(2*S*)-isopropyl 2-(((((2*R*,3*S*,4*R*,5*R*)-5-(2,4-dioxo-3,4-dihydropyrimidin-1(2*H*)-yl)-3,4-dihydroxy-4-methyltetrahydrothiophen-2-yl)methoxy)(phenoxy)phosphoryl)amino)propanoate (**13a**):

Following the general phosphoramidation procedure, thionucleoside **11a** (51 mg, 0.19 mmol) was treated with **12** (84 mg, 0.19 mmol) to afford the desired product (45 mg, 45% yield); ^1^H NMR (600 MHz, CD_3_OD) δ 8.08 (d, *J* = 8.2 Hz, 1H), 7.37 (t, *J* = 7.9 Hz, 2H), 7.29–7.26 (m, 2H), 7.23–7.17 (m, 1H), 5.99 (s, 1H), 5.68 (d, *J* = 8.1 Hz, 1H), 4.97 (app hept, *J* = 6.3 Hz, 1H), 4.56–4.53 (m, 1H), 4.49–4.46 (m, 1H), 3.93 (dq, *J* = 9.7, 7.1 Hz, 1H), 3.70 (d, *J* = 9.6 Hz, 1H), 3.68–3.65 (m, 1H), 1.36 (d, *J* = 7.1 Hz, 3H), 1.22 (d, *J* = 6.3 Hz, 6H), 1.17 (s, 3H) ppm; ^13^C NMR (151 MHz, CD_3_OD) δ 174.4 (d, ^3^*J_C–P_* = 5.4 Hz), 165.7, 152.9, 152.1 (d, ^2^*J_C–P_* = 6.7 Hz), 143.6, 130.9, 126.2, 121.3 (d, ^3^*J_C–P_* = 4.9 Hz), 102.9, 82.8, 77.6, 70.2, 68.2, 67.6 (d, ^2^*J_C–P_* = 5.1 Hz), 51.7, 51.1 (d, ^2^*J_C–P_* = 8.4 Hz), 22.0, 21.9, 20.7, 20.6 ppm; ^31^P NMR (121 MHz, CD_3_OD) δ 3.6 ppm; HRMS (ESI) *m*/*z*: [M + H]^+^ Calcd for C_22_H_31_N_3_O_9_PS 544.1513; Found 544.1511.

(*S*)-isopropyl 2-(((*S*)-(((2*R*,3*S*,4*R*,5*R*)-5-(5-fluoro-2,4-dioxo-3,4-dihydropyrimidin-1(2*H*)-yl)-3,4-dihydroxy-4-methyltetrahydrothiophen-2-yl)methoxy)(phenoxy)phosphoryl)amino)propanoate (**13b**):

Following the general phosphoramidation procedure, thionucleoside **11b** (38 mg, 0.13 mmol) was treated with **12** (59 mg, 0.13 mmol) to afford the desired product (16 mg, 22% yield); ^1^H NMR (500 MHz, CD_3_OD) δ 8.21 (d, ^3^*J*_H–F_ = 6.8 Hz, 1H), 7.37–7.324(m, 2H), 7.27–7.25 (m, 2H), 7.20–7.17 (m, 1H), 5.96 (d, ^5^*J*_H–F_ = 1.7 Hz, 1H), 4.97 (app hept, *J* = 6.2 Hz, 1H), 4.55–4.52 (m, 1H), 4.50–4.46 (m, 1H), 3.92 (dq, *J* = 10.0, 7.1 Hz, 1H), 3.71 (d, *J* = 9.6 Hz, 1H), 3.67–3.63 (m, 1H), 1.35 (d, *J* = 7.1 Hz, 3H), 1.22 (d, J = 6.3 Hz, 6H), 1.20 (s, 3H) ppm; ^13^C NMR (151 MHz, MeOD) δ 174.3 (d, ^3^*J_C–P_* = 5.4 Hz), 159.0 (d, ^2^*J_C–F_* = 26.4 Hz), 152.0 (d, ^2^*J_C–P_* = 6.5 Hz), 151.5, 141.2 (d, ^1^*J_C–F_* = 235.4 Hz), 130.7, 127.1 (d, ^2^*J_C–F_* = 35.0 Hz), 126.1, 121.3 (d, ^3^*J_C–P_* = 4.8 Hz), 82.7, 77.4, 70.1, 68.6, 67.3 (d, ^2^*J_C–P_* = 4.8 Hz), 51.6, 51.0 (d, ^2^*J_C–P_* = 8.6 Hz), 22.0, 21.9, 20.62, 20.59 (d, ^3^*J_C–P_* = 2.1 Hz) ppm; ^19^F NMR (282 MHz, CD_3_OD) δ-165.6 (d, ^3^*J*_F–H_ = 6.3 Hz); ^31^P NMR (121 MHz, CD_3_OD) δ 3.7 ppm; HRMS (NSI) *m*/*z*: [M + H]^+^ Calcd for C_22_H_30_N_3_O_9_FPS 562.1419; Found 562.1411.

(*S*)-isopropyl 2-(((*S*)-(((2*R*,3*S*,4*R*,5*R*)-5-(6-amino-9*H*-purin-9-yl)-3,4-dihydroxy-4-methyltetrahydrothiophen-2-yl)methoxy)(phenoxy)phosphoryl)amino)propanoate (**13c**):

Following the general phosphoramidation procedure, thionucleoside **11c** (86 mg, 0.29 mmol) was treated with **12** (131 mg, 0.29 mmol) to afford the desired product after two rounds of purification (40 mg, 25% yield); ^1^H NMR (300 MHz, CD_3_OD) δ 8.44 (s, 1H), 8.22 (s, 1H), 7.43–7.31 (m, 2H), 7.35–7.24 (m, 2H), 7.25–7.13 (m, 1H), 5.87 (s, 1H), 5.05–4.90 (m, 1H), 4.66–4.52 (m, 2H), 4.11 (d, *J* = 9.5 Hz, 1H), 3.94 (dq, *J* = 9.9, 7.1 Hz, 1H), 3.86–3.73 (m, 1H), 1.37 (dd, *J* = 7.1, 0.8 Hz, 3H), 1.20 (d, *J* = 6.2 Hz, 3H), 1.15 (d, *J* = 6.3 Hz, 3H), 0.93 (s, 3H) ppm; ^13^C NMR (151 MHz, CD_3_OD) δ 174.4 (d, ^3^*J_C–P_* = 5.5 Hz), 157.5, 154.0, 152.2 (d, ^2^*J_C–P_* = 6.7 Hz), 151.2, 141.9, 130.8, 126.2, 121.4 (d, ^3^*J_C–P_* = 4.8 Hz), 120.2, 83.0, 77.7, 70.1, 68.4 (d, ^2^*J_C–P_* = 5.0 Hz), 66.4, 51.7, 51.2 (d, ^2^*J_C–P_* = 8.5 Hz), 22.0, 21.9, 20.9, 20.7 (d, ^3^*J_C–P_* = 6.3 Hz) ppm; ^31^P NMR (121 MHz, CD_3_OD) δ 3.7 ppm; HRMS (ESI) *m*/*z*: [M + H]^+^ Calcd for C_23_H_32_N_6_O_7_PS 567.1785; Found 567.1778.

(2*S*)-isopropyl 2-(((((2*R*,3*S*,4*R*,5*R*)-5-(3,5-dioxo-4,5-dihydro-1,2,4-triazin-2(3*H*)-yl)-3,4-dihydroxy-4-methyltetrahydrothiophen-2-yl)methoxy)(phenoxy)phosphoryl)amino)propanoate (**13d**):

Following the general phosphoramidation procedure, thionucleoside **11d** (60 mg, 0.22 mmol) was treated with **12** (100 mg, 0.22 mmol) to afford the desired product (27 mg, 23% yield); ^1^H NMR (600 MHz, CD_3_OD) δ 7.49 (s, 1H), 7.35 (t, *J* = 7.9 Hz, 2H), 7.27–7.21 (m, 2H), 7.21–7.15 (m, 1H), 6.03 (s, 1H), 4.95 (dq, *J* = 12.5, 6.3 Hz, 1H), 4.59 (ddd, *J* = 10.0, 6.0, 3.9 Hz, 1H), 4.28–4.21 (m, 1H), 4.04 (d, *J* = 8.7 Hz, 1H), 3.90 (dq, *J* = 9.7, 7.1 Hz, 1H), 3.64 (td, *J* = 8.9, 3.9 Hz, 1H), 1.34 (d, *J* = 7.1 Hz, 3H), 1.22 (d, *J* = 6.3 Hz, 6H), 1.20 (s, 3H) ppm; ^13^C NMR (151 MHz, CD_3_OD) δ 174.4 (d, ^3^*J_C–P_* = 5.9 Hz), 158.3, 152.2 (d, ^2^*J_C–P_* = 6.9 Hz), 150.2, 137.5, 130.7, 126.1, 121.4 (d, ^3^*J_C–P_* = 4.8 Hz), 83.3, 79.3, 70.9 (d, ^2^*J_C–P_* = 5.3 Hz), 70.1, 51.6, 51.5 (d, ^2^*J_C–P_* = 8.0 Hz), 22.0, 21.9, 20.7, 20.6 (d, ^3^*J_C–P_* = 6.1 Hz) ppm; ^31^P NMR (121 MHz, CD_3_OD) δ 3.3 ppm; HRMS (ESI-) *m*/*z*: [M + Cl]^−^ Calcd for C_21_H_29_N_4_O_9_ClPS 579.1087; Found 579.1090.

Isopropyl (2*S*)-2-[[[(2*R*,3*S*,4*R*,5*R*)-5-(4-amino-5-fluoro-2-oxo-pyrimidin-1-yl)-3,4-dihydroxy-4-methyl-tetrahydrothiophen-2-yl]methoxy-phenoxy-phosphoryl]amino]propanoate (**13e**):

Following the general phosphoramidation procedure, thionucleoside **11e** (72 mg, 0.25 mmol) was treated with **12** (224 mg, 0.5 mmol) to afford the desired product after three rounds of purification (12 mg, 8% yield); ^1^H NMR (600 MHz, MeOD) δ 8.19 (d, ^3^*J*_H–F_ = 6.8 Hz, 1H), 7.39–7.33 (m, 2H), 7.29–7.26 (m, 2H), 7.20–7.18 (m, 1H), 6.09 (d, ^5^*J*_H–F_ = 1.8 Hz, 1H), 4.97 (app hept, *J* = 6.3 Hz, 1H), 4.57–4.52 (m, 1H), 4.50–4.46 (m, 1H), 3.93 (dq, *J* = 10.1, 7.1 Hz, 1H), 3.69 (d, *J* = 9.7 Hz, 1H), 3.67–3.63 (m, 1H), 1.35 (d, *J* = 7.1 Hz, 3H), 1.22 (d, *J* = 6.2 Hz, 3H), 1.21 (d, *J* = 6.3 Hz, 3H), 1.15 (s, 3H) ppm; ^13^C NMR (151 MHz, CD_3_OD) δ 174.4 (d, ^3^*J_C–P_* = 5.4 Hz), 159.3 (d, ^2^*J_C–F_* = 14.0 Hz), 157.4, 152.2 (d, ^2^*J_C–P_* = 6.6 Hz), 138.0 (d, ^1^*J_C–F_* = 244.9 Hz), 130.8, 127.9 (d, ^2^*J_C–F_* = 32.8 Hz), 126.1, 121.4 (d, ^3^*J_C–P_* = 4.9 Hz), 82.9, 77.4, 70.2, 69.3, 67.6 (d, ^2^*J_C–P_* = 4.8 Hz), 51.7, 51.0 (d, ^2^*J_C–P_* = 8.6 Hz), 22.0, 21.9, 20.63, 20.59 ppm; ^19^F NMR (282 MHz, CD_3_OD) δ-165.7 (d, ^3^*J*_F–H_ = 5.6 Hz) ppm; ^31^P NMR (121 MHz, CD_3_OD) δ 3.7 ppm; HRMS (NSI) *m*/*z*: [M + H]^+^ Calcd for C_22_H_31_N_4_O_8_FPS 561.1579; Found 561.1578.

Isopropyl (2*S*)-2-[[[(2*R*,3*S*,4*R*,5*R*)-5-(4-amino-2-oxo-pyrimidin-1-yl)-3,4-dihydroxy-4-methyl-tetrahydrothiophen-2-yl]methoxy-phenoxy-phosphoryl]amino]propanoate (**13f**):

An oven-dried 50-mL round-bottom flask with stir bar and reflux condenser was charged with thionucleoside **11f** (80.5 mg, 0.29 mmol), and the apparatus and solid were dried overnight in vacuo. The flask was then charged with argon, phenylboronic acid (38 mg, 0.31 mmol), and anhydrous pyridine (0.75 mL) to give a yellow solution. The reaction was heated to 100 °C for approximately 4 h before the volatiles were removed. The crude yellow semi-solid was dried briefly in vacuo before the flask was again charged with argon followed by **12** (160 mg, 0.35 mmol) and anhydrous pyridine (1 mL) to give a yellow solution. The solution was chilled to 0 °C before Me_2_AlCl (0.15 mL, 0.15 mmol) was dropwise added as a 1 M solution in hexanes, and the reaction was warmed to 50 °C with stirring overnight. The next morning, the reaction was cooled to ambient temperature before being quenched with 0.4 mL of 30 wt% aqueous L-tartaric acid solution. The reaction mixture was then diluted with ethyl acetate and aqueous brine solution before being extracted with ethyl acetate. The organic extracts were combined, dried over Na_2_SO_4_, filtered, concentrated and purified via two rounds of silica gel flash column chromatography (eluted with a gradient of 0–15% methanol in DCM) to afford the desired product (59 mg, 37% yield); ^1^H NMR (600 MHz, CD_3_OD) δ 8.09 (d, *J* = 7.5 Hz, 1H), 7.41–7.35 (m, 2H), 7.31–7.26 (m, 2H), 7.23–7.17 (m, 1H), 6.16 (s, 1H), 5.90 (d, *J* = 7.4 Hz, 1H), 4.97 (app hept, *J* = 6.3 Hz, 1H), 4.57–4.51 (m, 1H), 4.50–4.43 (m, 1H), 3.93 (dq, *J* = 9.9, 7.1 Hz, 1H), 3.67 (br, 1H), 1.36 (d, *J* = 7.1 Hz, 3H), 1.22 (d, *J* = 6.2 Hz, 3H), 1.21 (d, *J* = 6.3 Hz, 3H), 1.12 (s, 3H) ppm; ^13^C NMR (151 MHz, CD_3_OD) δ 174.4 (d, ^3^*J_C–P_* = 5.4 Hz), 167.1, 159.0, 152.2 (d, ^2^*J_C–P_* = 6.7 Hz), 144.1, 130.9, 126.2, 121.3 (d, ^3^*J_C–P_* = 4.8 Hz), 96.5, 82.9, 77.5, 70.2, 68.7, 67.7 (d, ^2^*J_C–P_* = 5.0 Hz), 51.7, 50.9 (d, ^2^*J_C–P_* = 8.6 Hz), 22.0, 21.9, 20.7, 20.6 ppm; ^31^P NMR (121 MHz, CD_3_OD) δ 3.5 ppm; HRMS (NSI) *m*/*z*: [M + H]^+^ Calcd for C_22_H_32_N_4_O_8_PS 543.1673; Found 543.1676.

### 3.2. Pharmacology

#### Anti-HCV and Cytotoxicity Evaluation

For in vitro evaluation of the antiviral and cytotoxic effects, the compounds were submitted to ImQuest Biosciences, Inc. (Frederick, Maryland 21704) as a contracted fee-for-service body of work. According to their standard operating procedure, Huh-7 luc/neo ET cells bearing a dicistronic HCV genotype 1b luciferase reporter replicon were plated at 7.5 × 10^3^ cells/mL in duplicate 96-well plates for the parallel determination of antiviral efficacy (EC_50_) and cytotoxicity (TC_50_). These plates were cultured for 24 h prior to the addition of compounds. Six serial half-log dilutions of the test articles (high test of 1 μg/mL) and sofosbuvir (high test 1.0 μM) were prepared in cell culture medium and added to the cultured cells in triplicate wells for each dilution. Six wells in the test plates received medium alone as an untreated control. Following 72 h of culture in the presence of the compound, one of the plates was used for the determination of cytotoxicity by staining with XTT (2,3-bis-(2-methoxy-4-nitro-5-sulfophenyl)-2H-tetrazolium-5-carboxanilide) and the other for antiviral efficacy by determination of luciferase reporter activity. Cytotoxicity and efficacy data were collected and imported into a customized Microsoft Excel™ workbook for the determination of TC_50_ and EC_50_ values.

## Figures and Tables

**Table 1:** Antiviral dose-response data for 2′-*C*-methyl-4′-thionucleoside phosphoramidates^.1^.

Entry	Compound No.	Nucleobase (R)	EC_50_ (μM)	TC_50_ (μM)	TI^2^
1	**13a**	uracil	2.10	>200	>95.2
2	**13b**	5-fluorouracil	113	>200	>1.77
3	**13c**	adenine	36.2	>200	>5.52
4	**13d**	6-azauracil	>200	>200	ND
5	**13e**	5-fluorocytosine	124	>200	>1.61
6	**13f**	cytosine	9.82	>200	>20.4
7	–	control^3^	0.05	>2	>42.5

^1^ Values reported are calculated from triplicate dose-response data from a single experiment; ^2^ therapeutic index (TI) = antiviral efficacy (EC_50_)/cytotoxicity (TC_50_); ^3^ Sofosbuvir was used as the positive control.

## Supplementary Materials

The following are available online. ^1^H, ^13^C, ^19^F and ^31^P NMR spectra of disclosed compounds.
