# Supplementary material for: Synthesis and Antiviral Activity of a Series of 2′-C-Methyl-4′-thionucleoside Monophosphate Prodrugs
Source: Molecules. 2020 Nov 6;25(21):5165. doi: 10.3390/molecules25215165 (PMC7664256; doi:10.3390/molecules25215165)
Supplement: Supplementary file 1 [file molecules-25-05165-s001.pdf]

Supplementary Material

Synthesis and Antiviral Activity of a Series of 2′-*C*-Methyl-4′-thionucleoside Monophosphate Prodrugs

Zackery W. Dentmon, Thomas M. Kaiser, and Dennis C. Liotta*

Department of Chemistry, Emory University

1521 Dickey Dr., Atlanta, GA 30322, USA

Correspondence: dliotta@emory.edu

Table of Contents

^{1}H and ^{13}C NMR Spectra for Intermediates **1.1 – 9** S2-S17

^{1}H, ^{13}C, and ^{19}F NMR Spectra for 4′-Thionucleosides **11a – 11f** S18-S31

^{1}H, ^{13}C, ^{19}F, and ^{31}P NMR Spectra for Phosphoramidates **13a – 13f** S32-S51

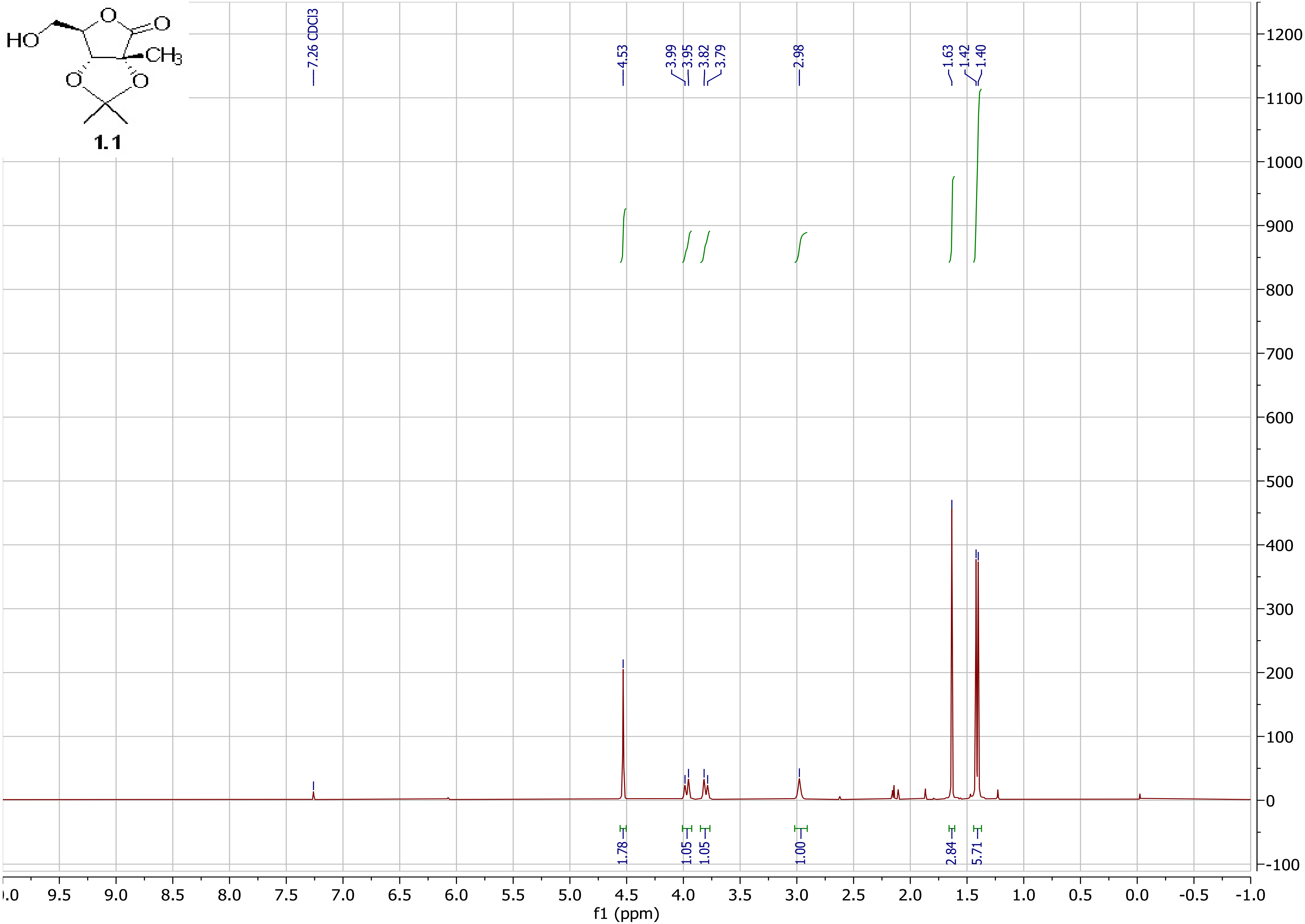
HO
O
O
CH3
O
O
1.1
7.26 CDCl3
4.53
3.99
3.95
3.82
3.79
2.98
1.63
1.42
1.40
1.78
1.05
1.05
1.00
2.84
5.71
f1 (ppm)

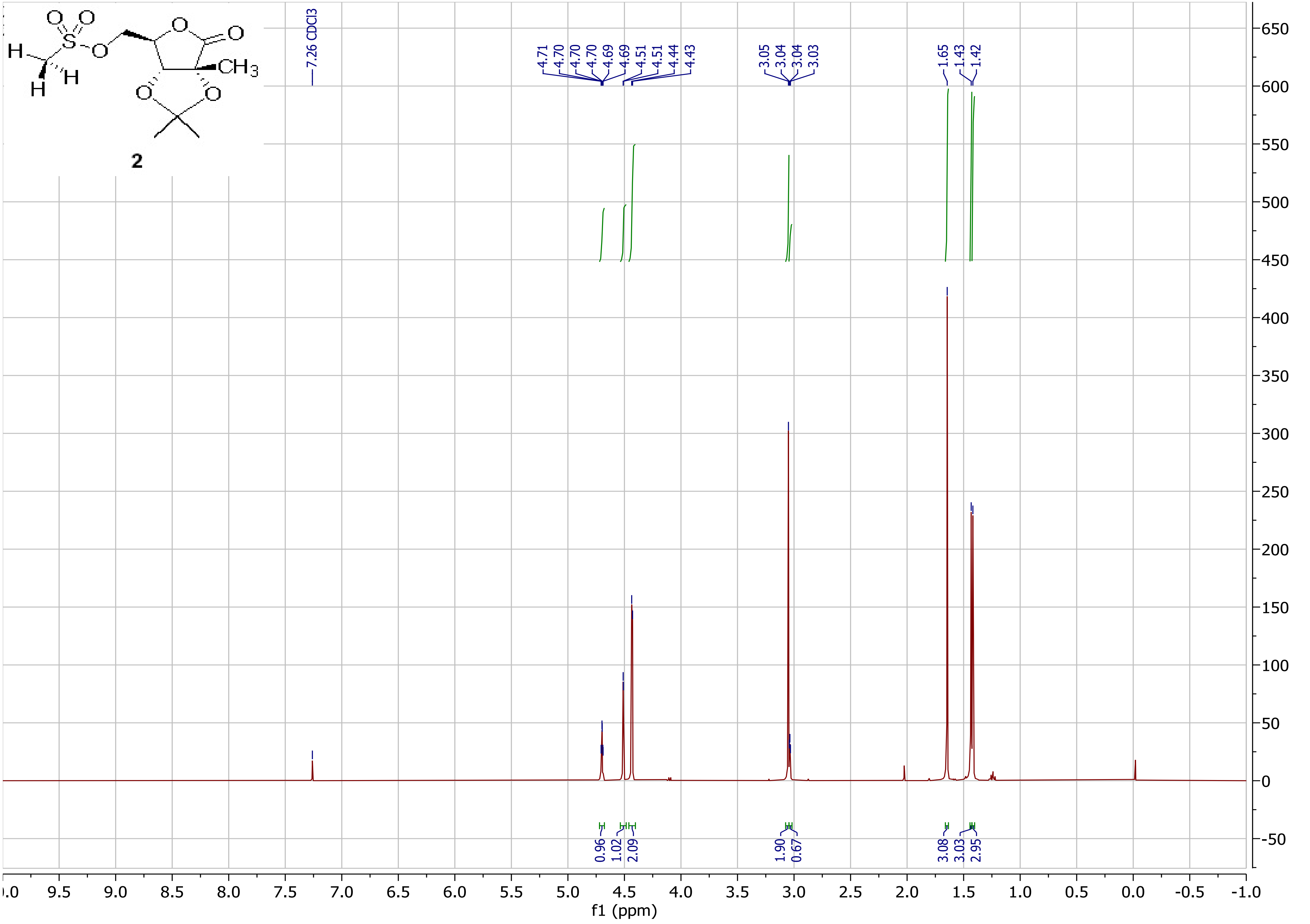
2
CH3
7.26 CDCl3
4.71 4.70 4.70 4.70 4.69 4.69 4.51 4.51 4.44 4.43
3.05 3.04 3.04 3.03
1.65 1.43 1.42
0.96 1.02 2.09
1.90 0.67
3.08 3.03 2.95
f1 (ppm)

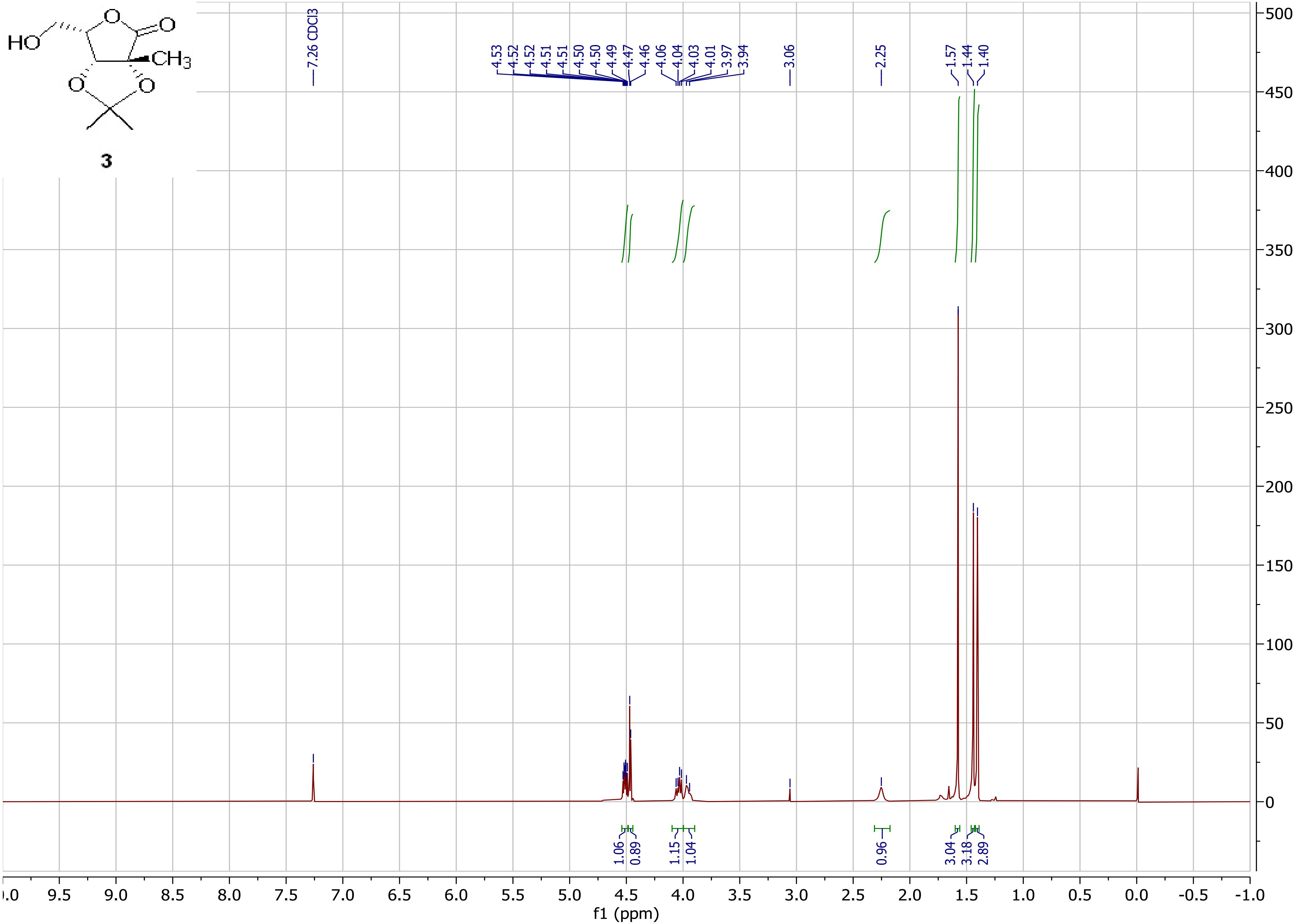
HO
O
O
CH3
O
O
3
7.26 CDCl3
4.53
4.52
4.52
4.51
4.51
4.50
4.50
4.49
4.47
4.46
4.06
4.04
4.03
4.01
3.97
3.94
3.06
2.25
1.57
1.44
1.40
1.06
0.89
1.15
1.04
0.96
3.04
3.18
2.89
f1 (ppm)

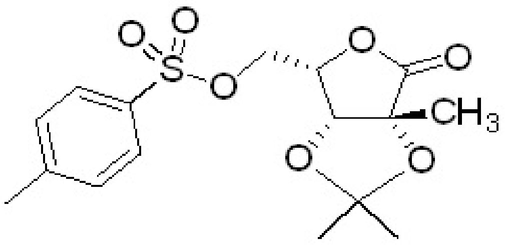

3.1

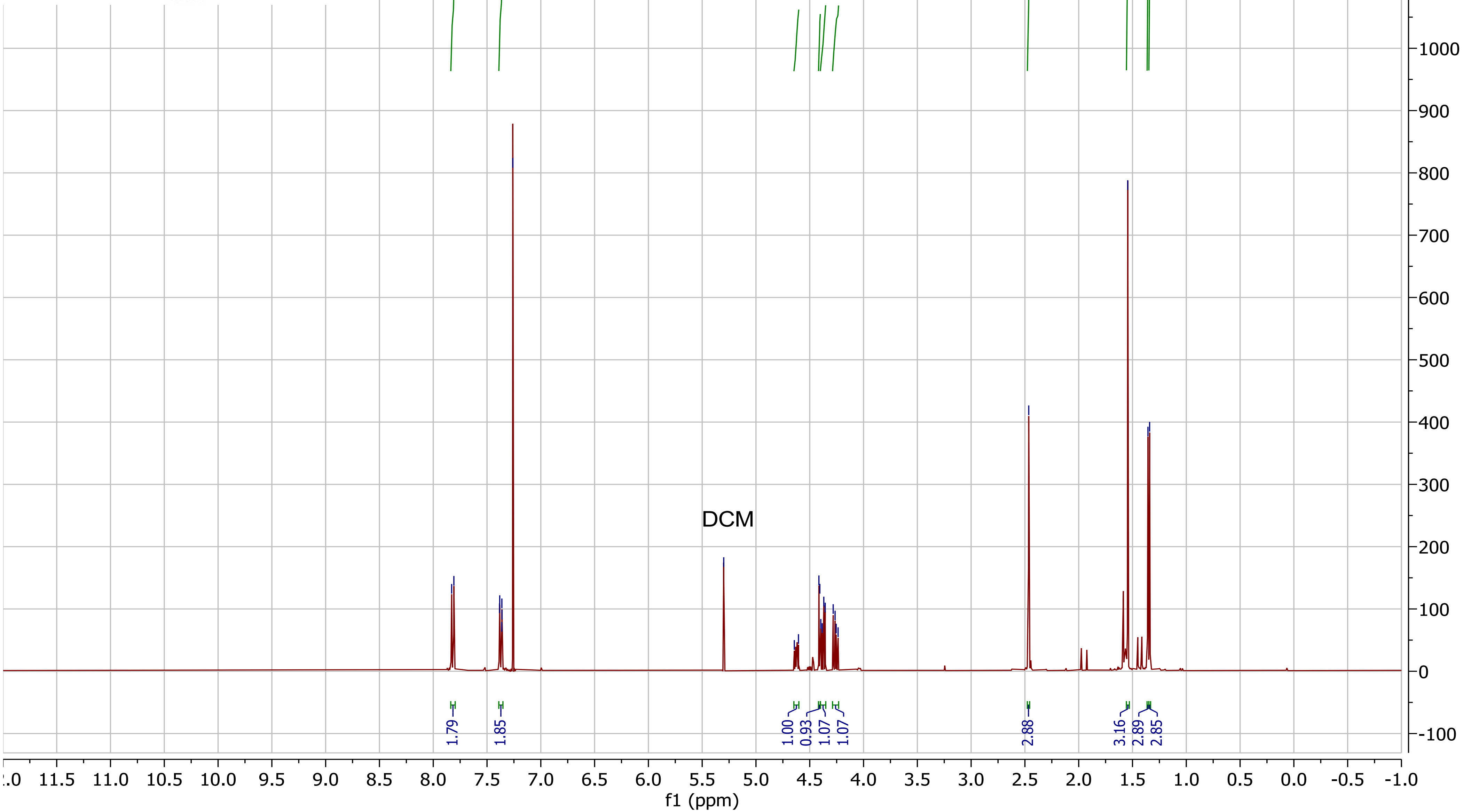
7.83
7.81
7.38
7.38
7.36
7.36
7.36
7.26 CDCl3
5.30
4.64
4.60
4.41
4.41
4.40
4.38
4.37
4.36
4.28
4.26
4.25
4.24
2.46
1.54
1.36
1.34
DCM
1.79
1.85
1.00
0.93
1.07
1.07
2.88
3.16
2.89
2.85
f1 (ppm)

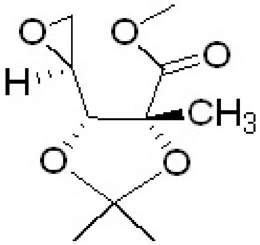

4

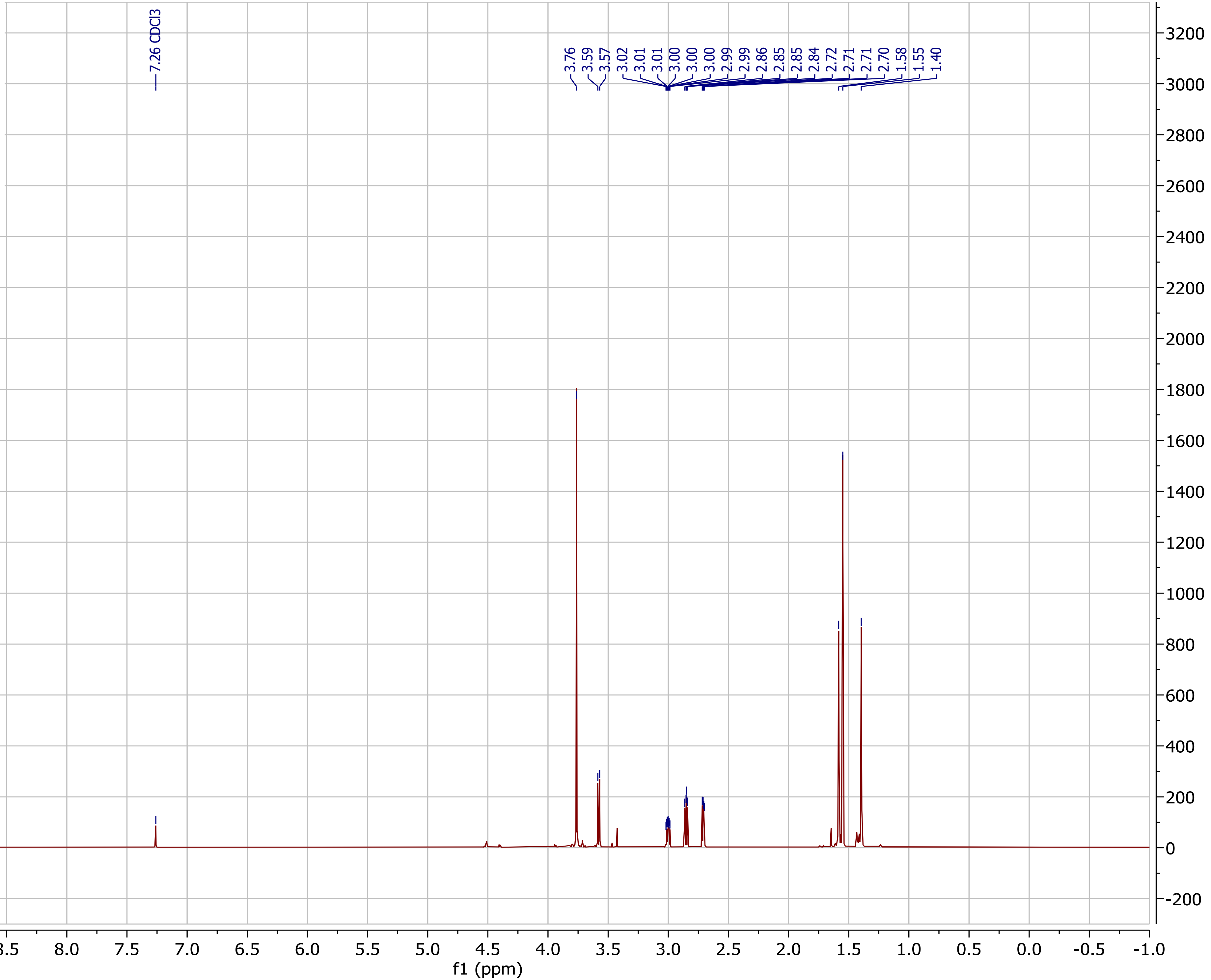
7.26 CDCl3
3.76
3.59
3.57
3.02
3.01
3.01
3.00
3.00
3.00
2.99
2.99
2.86
2.85
2.85
2.84
2.72
2.71
2.71
2.70
1.58
1.55
1.40
f1 (ppm)

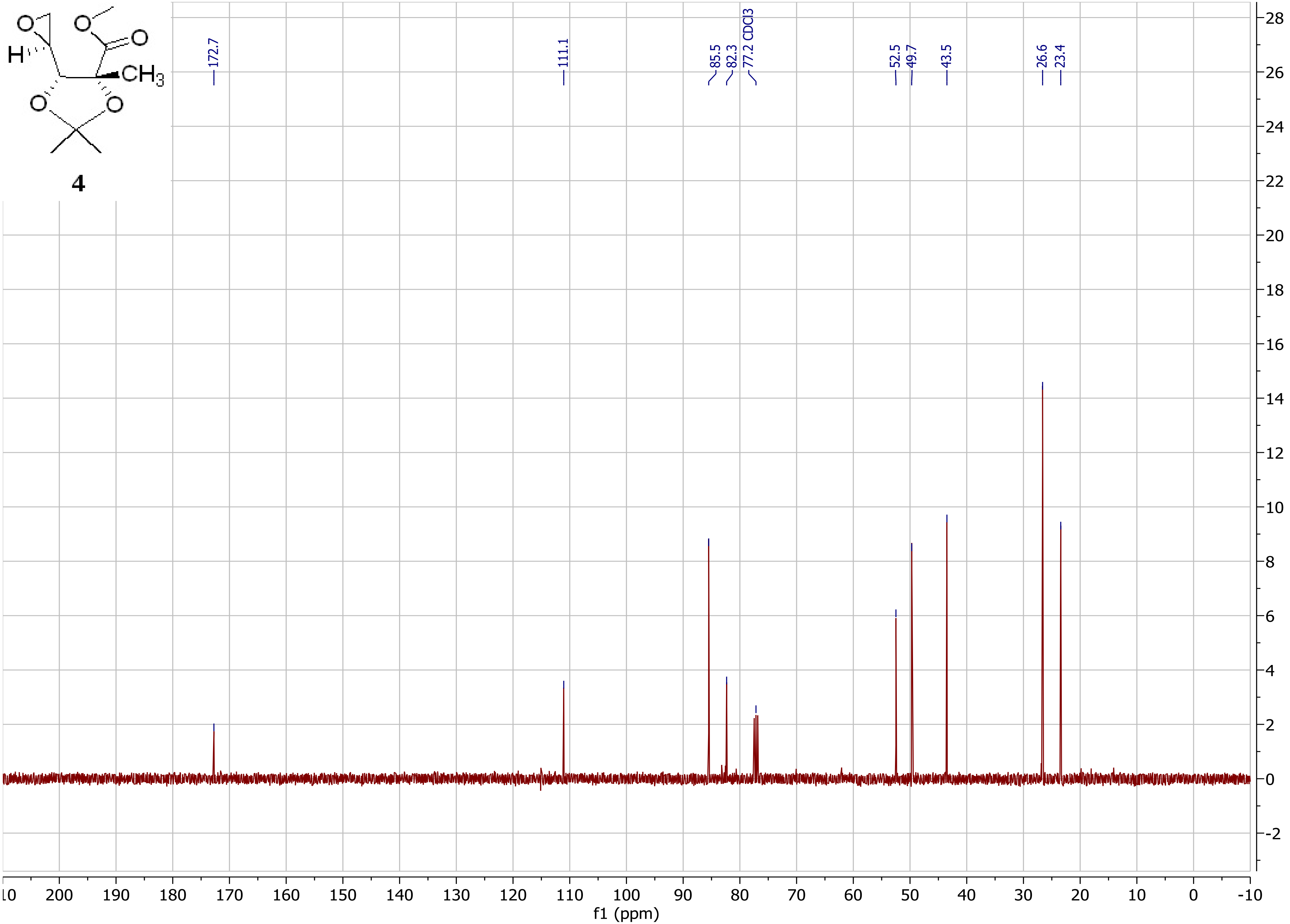

4

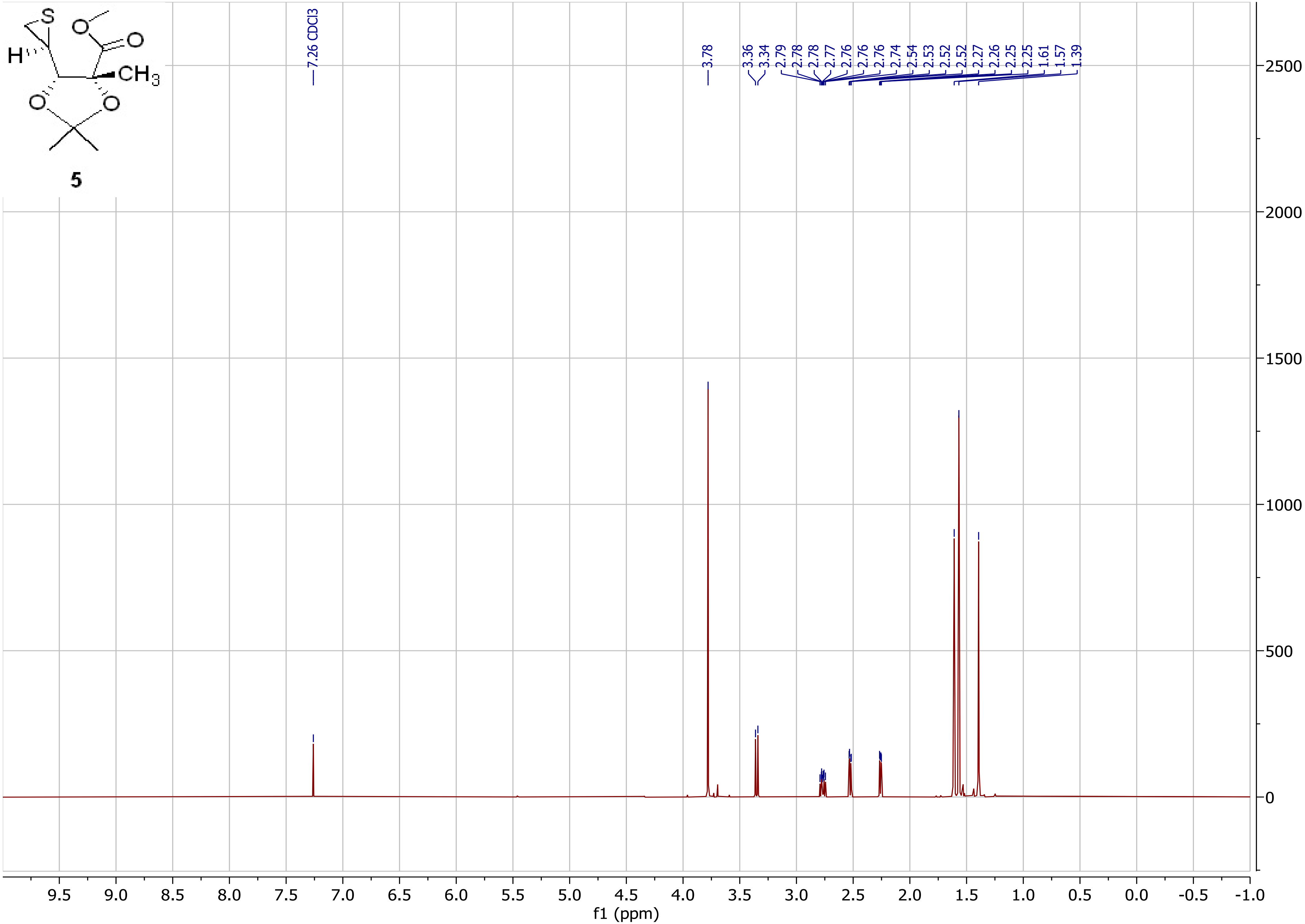

7.26 CDCl3
3.78
3.36
3.34
2.79
2.78
2.78
2.77
2.76
2.76
2.76
2.74
2.54
2.53
2.52
2.52
2.27
2.26
2.25
2.25
1.61
1.57
1.39
H
S
O
O
CH3
O
O
5
2500
2000
1500
1000
500
0
9.5 9.0 8.5 8.0 7.5 7.0 6.5 6.0 5.5 5.0 4.5 4.0 3.5 3.0 2.5 2.0 1.5 1.0 0.5 0.0 -0.5 -1.0
f1 (ppm)

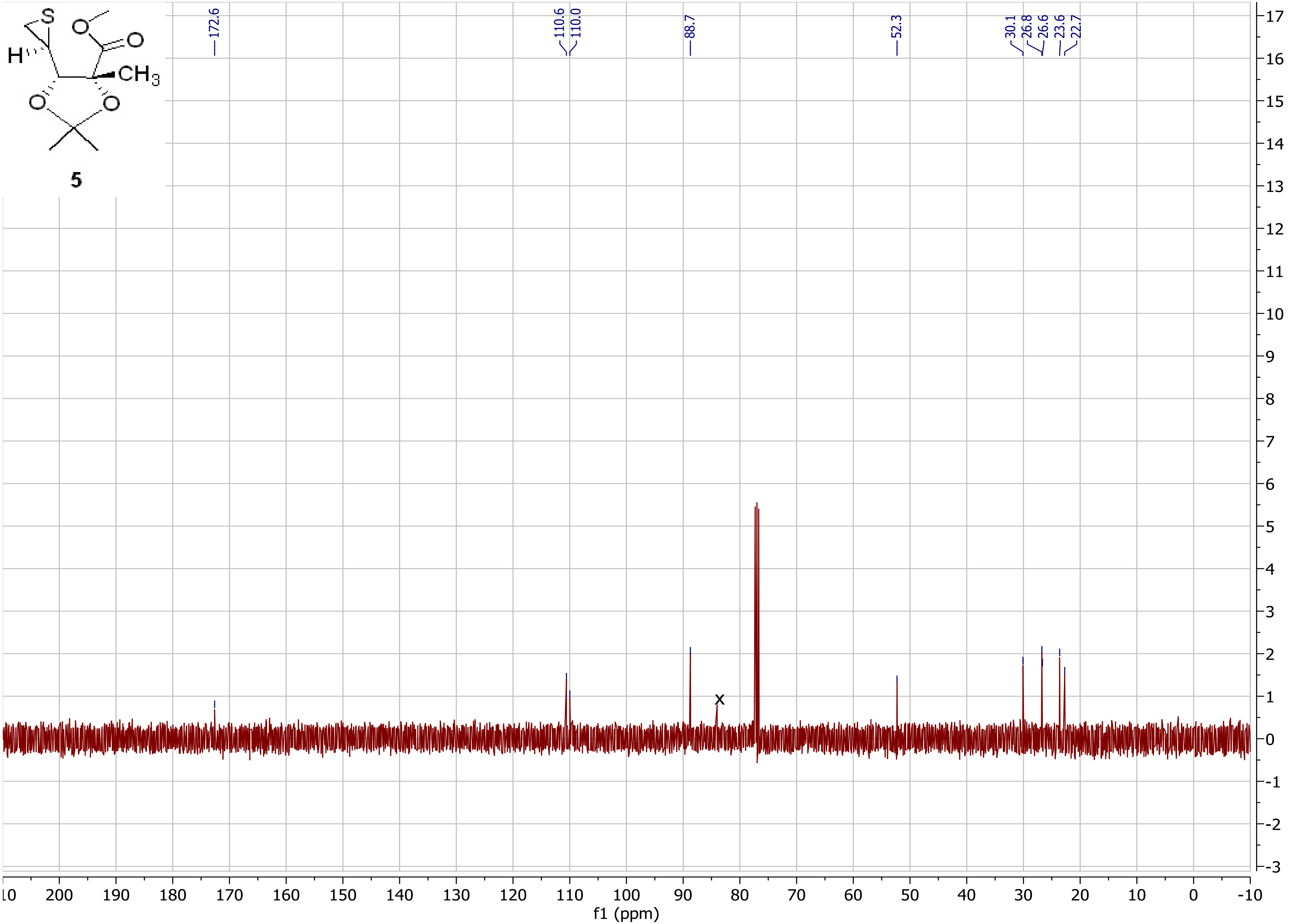

5

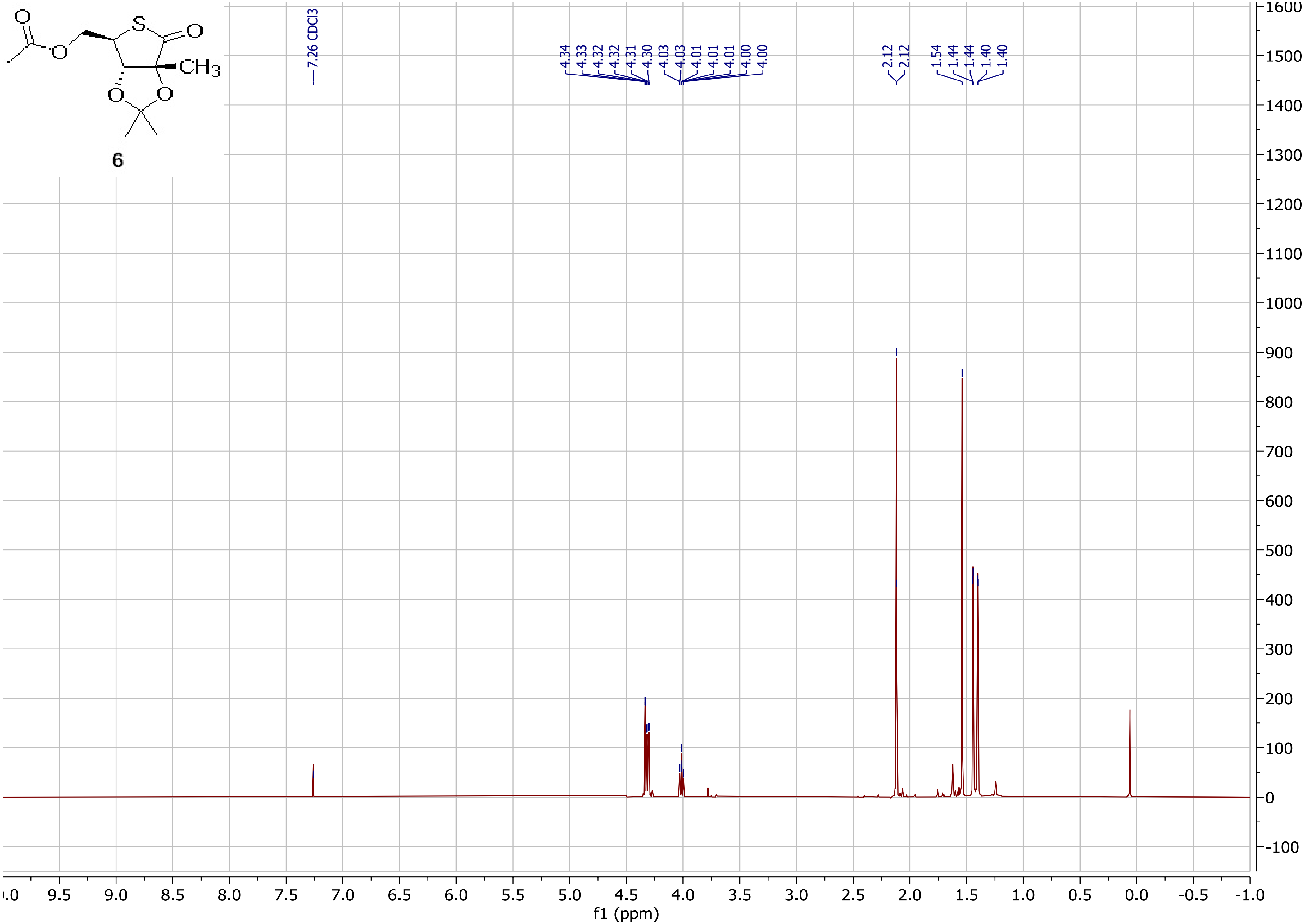

6
CH3
7.26 CDCl3
4.34
4.33
4.32
4.32
4.31
4.30
4.03
4.03
4.01
4.01
4.01
4.00
4.00
2.12
2.12
1.54
1.44
1.44
1.40
1.40
f1 (ppm)

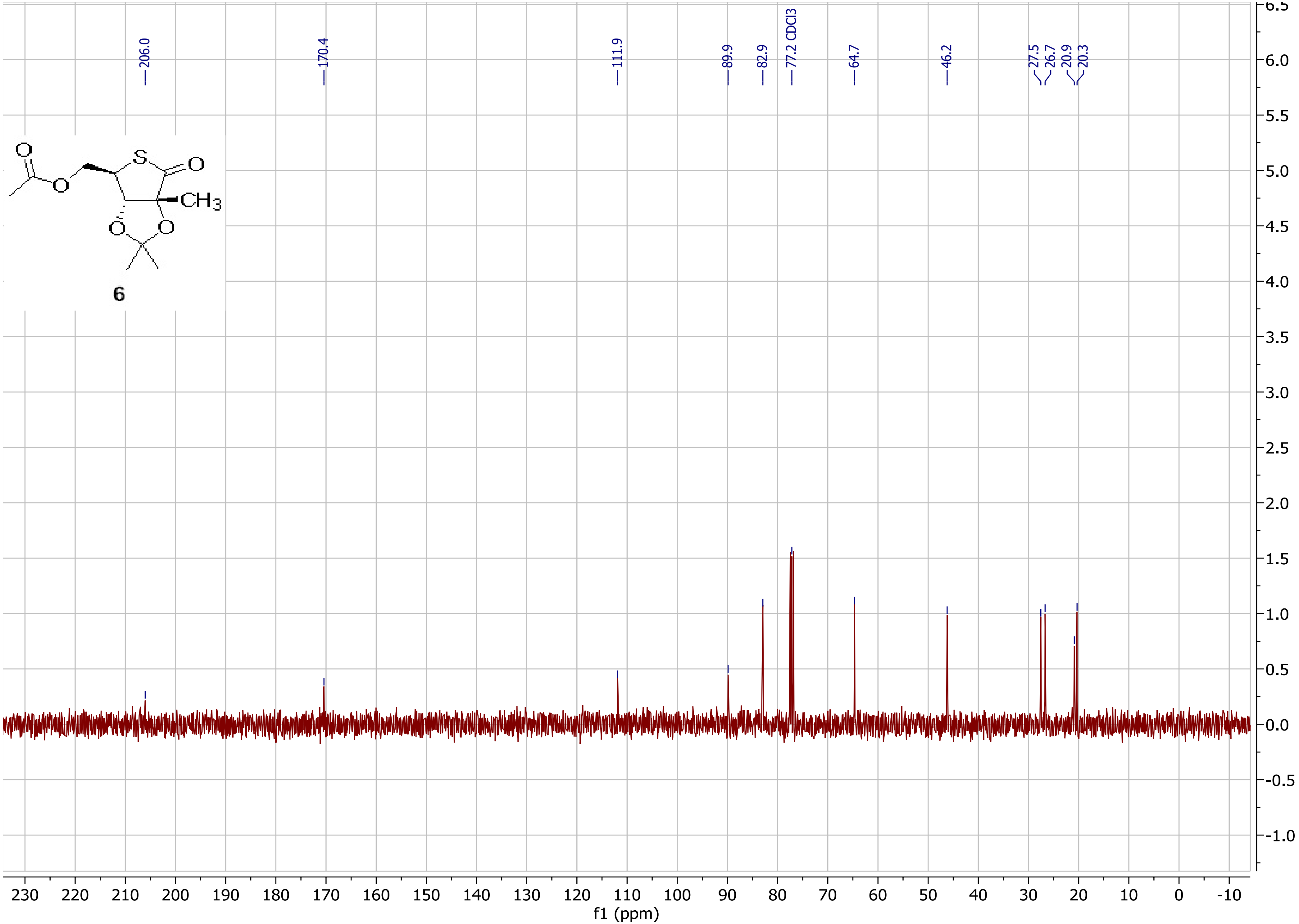
206.0
170.4
111.9
89.9
82.9
77.2 CDCl3
64.7
46.2
27.5
26.7
20.9
20.3
CH3
6
f1 (ppm)

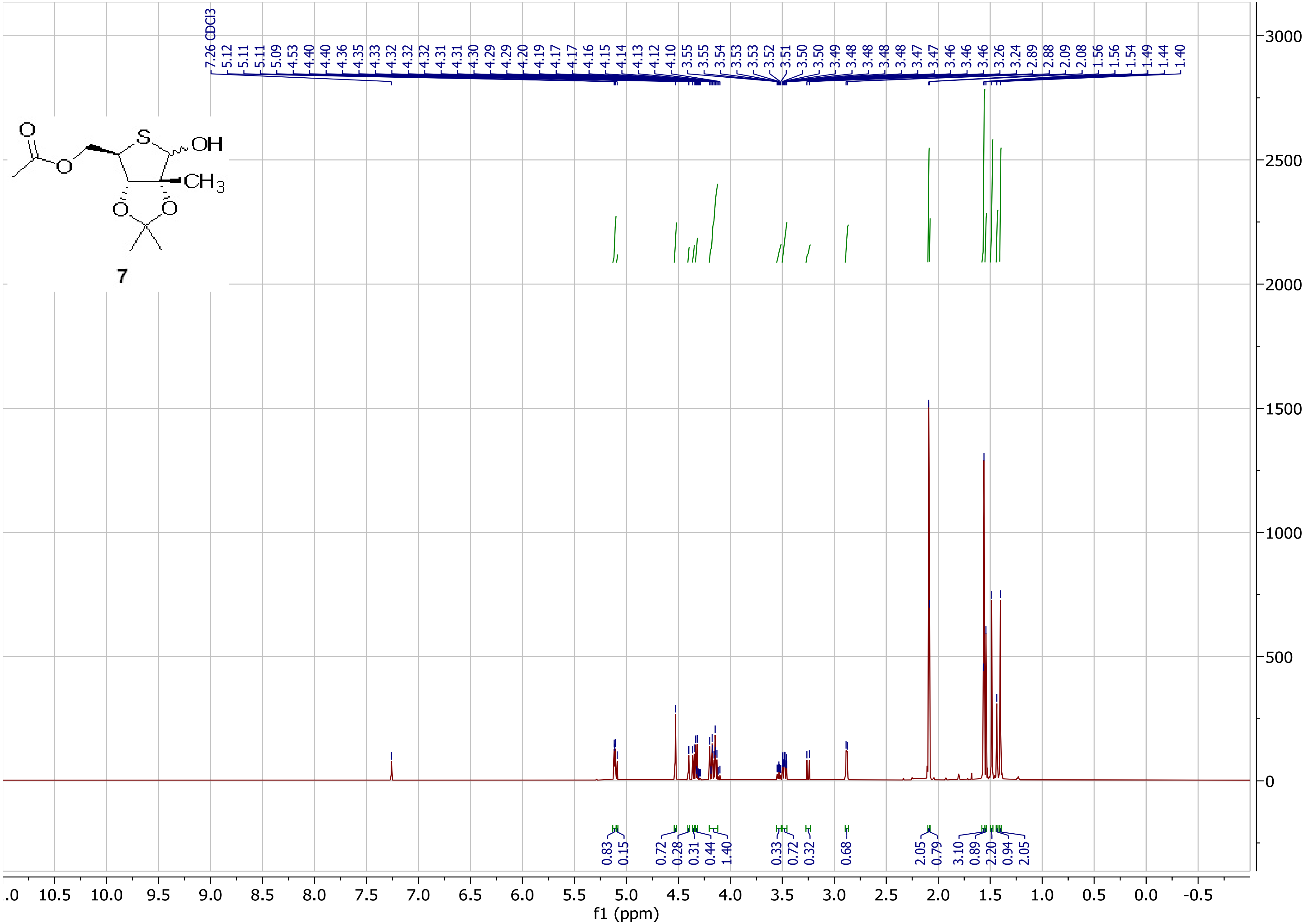
7.26 CDCl3
5.12
5.11
5.11
5.09
4.53
4.40
4.40
4.36
4.35
4.33
4.32
4.32
4.32
4.31
4.31
4.30
4.29
4.29
4.20
4.19
4.17
4.17
4.16
4.15
4.14
4.13
4.12
4.10
3.55
3.55
3.54
3.53
3.53
3.52
3.51
3.50
3.50
3.49
3.48
3.48
3.48
3.48
3.47
3.47
3.46
3.46
3.46
3.26
3.24
2.89
2.88
2.09
2.08
1.56
1.56
1.54
1.49
1.44
1.40
S
OH
CH3
7
0.83
0.15
0.72
0.28
0.31
0.44
1.40
0.33
0.72
0.32
0.68
2.05
0.79
3.10
0.89
2.20
0.94
2.05
f1 (ppm)

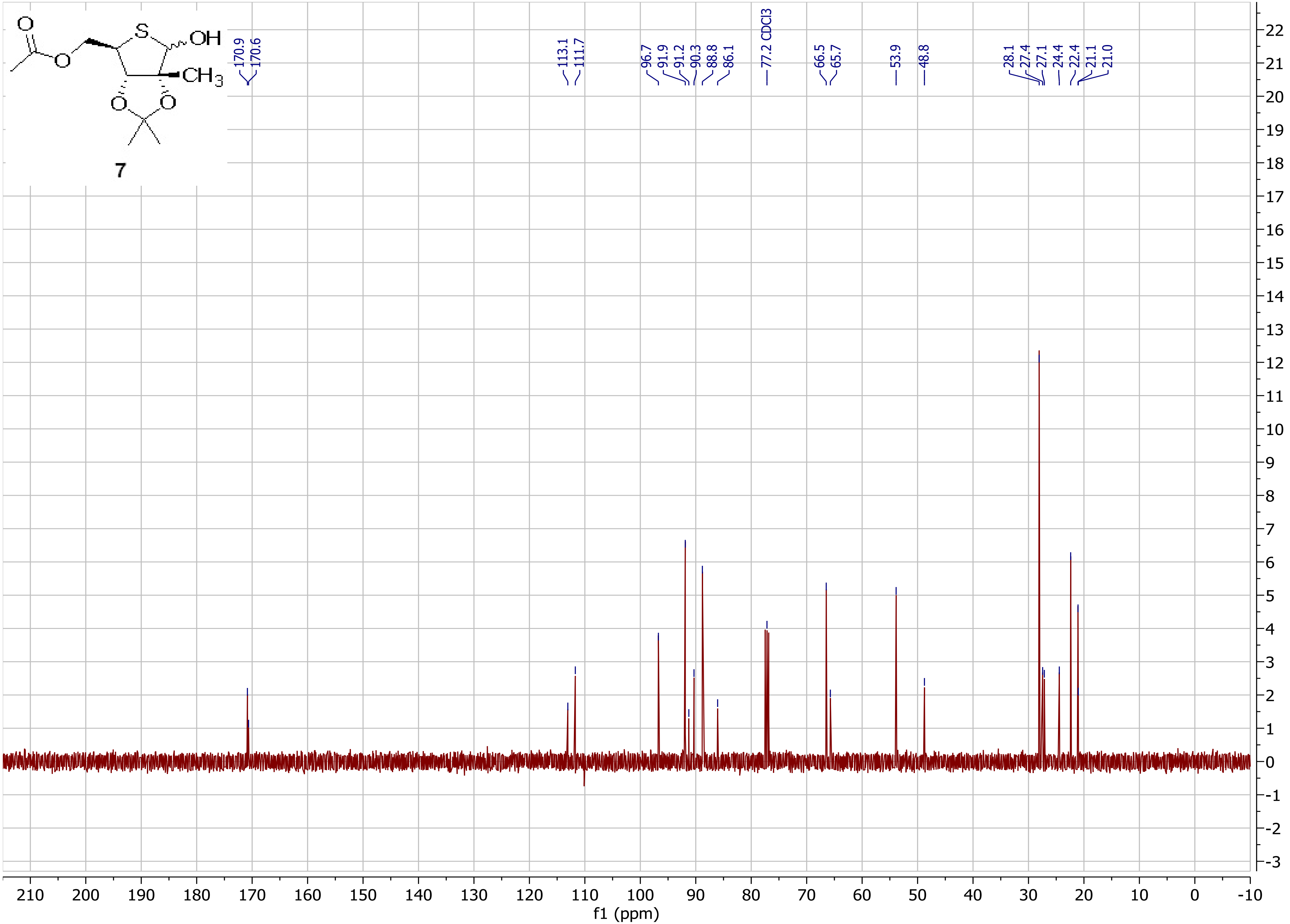
7
170.9
170.6
113.1
111.7
96.7
91.9
91.2
90.3
88.8
86.1
77.2 CDCl3
66.5
65.7
53.9
48.8
28.1
27.4
27.1
24.4
22.4
21.1
21.0
f1 (ppm)

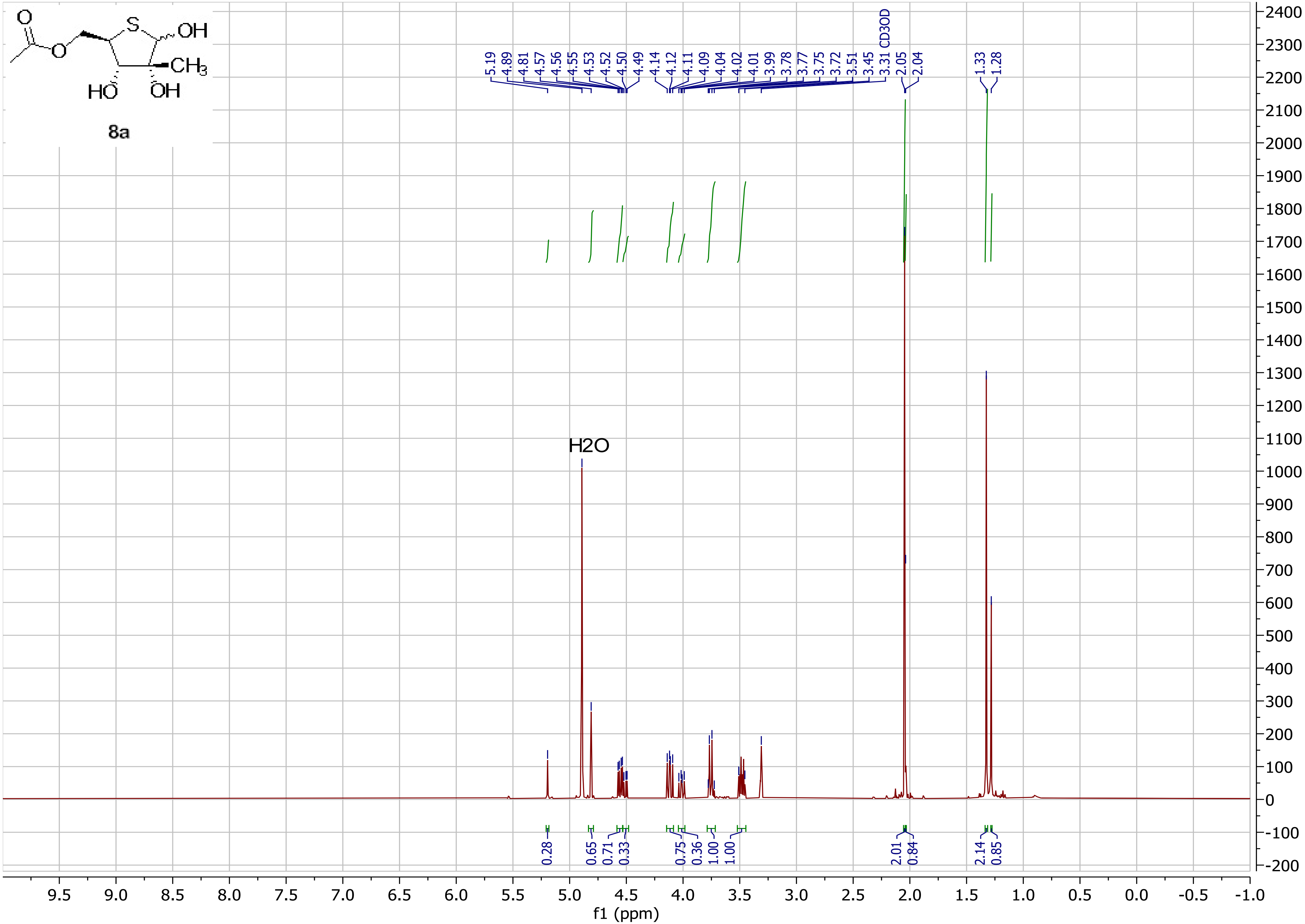

8a
H2O
CD3OD
5.19 4.89 4.81 4.57 4.56 4.55 4.53 4.52 4.50 4.49 4.14 4.12 4.11 4.09 4.04 4.02 4.01 3.99 3.78 3.77 3.75 3.72 3.51 3.45 3.31 2.05 2.04 1.33 1.28
0.28 0.65 0.71 0.33 0.75 0.36 1.00 1.00 2.01 0.84 2.14 0.85
f1 (ppm)

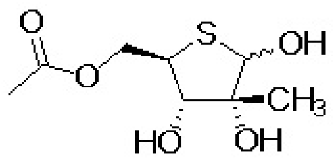

8a

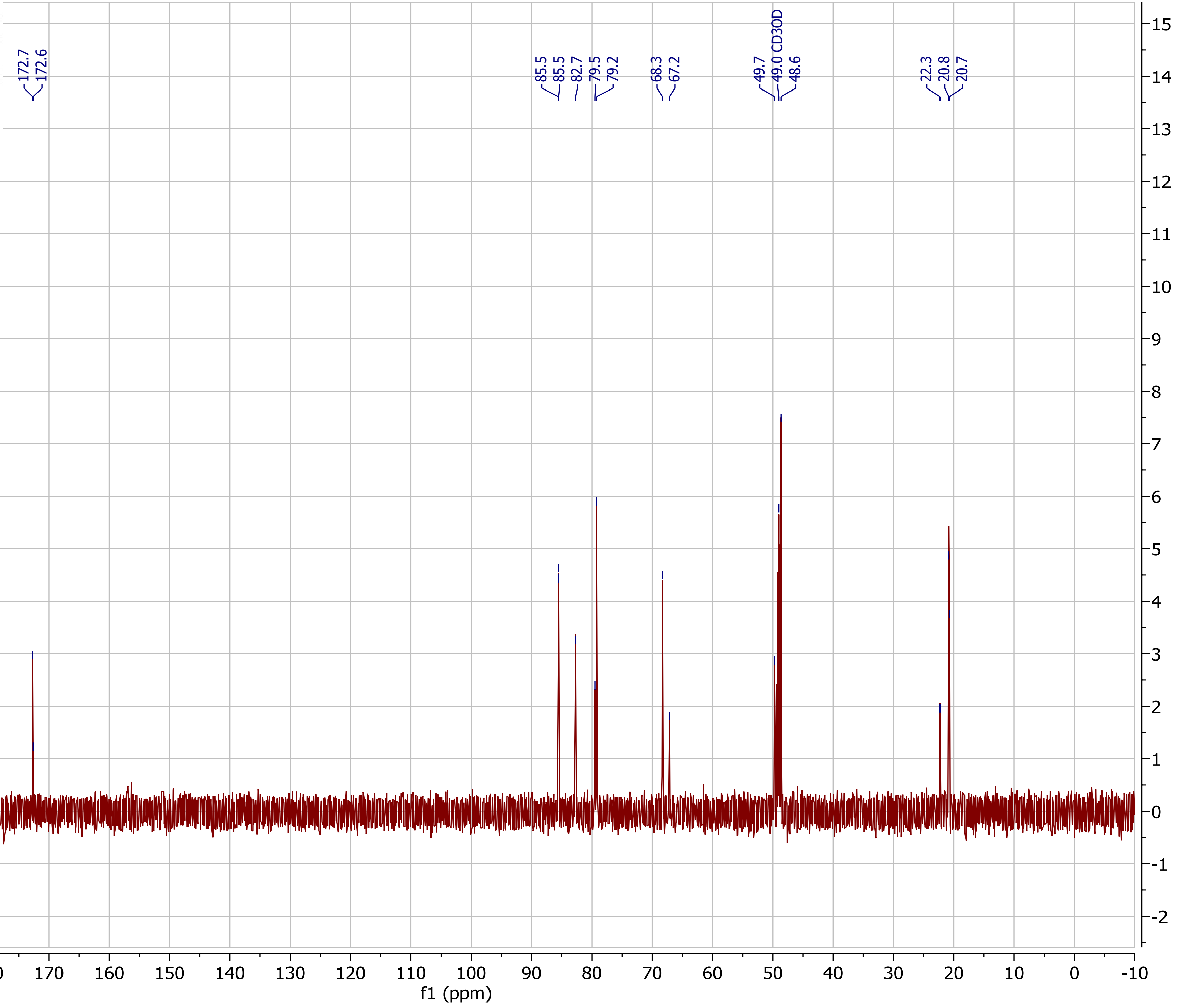

172.7
172.6
85.5
85.5
82.7
79.5
79.2
68.3
67.2
49.7
49.0 CD3OD
48.6
22.3
20.8
20.7
f1 (ppm)

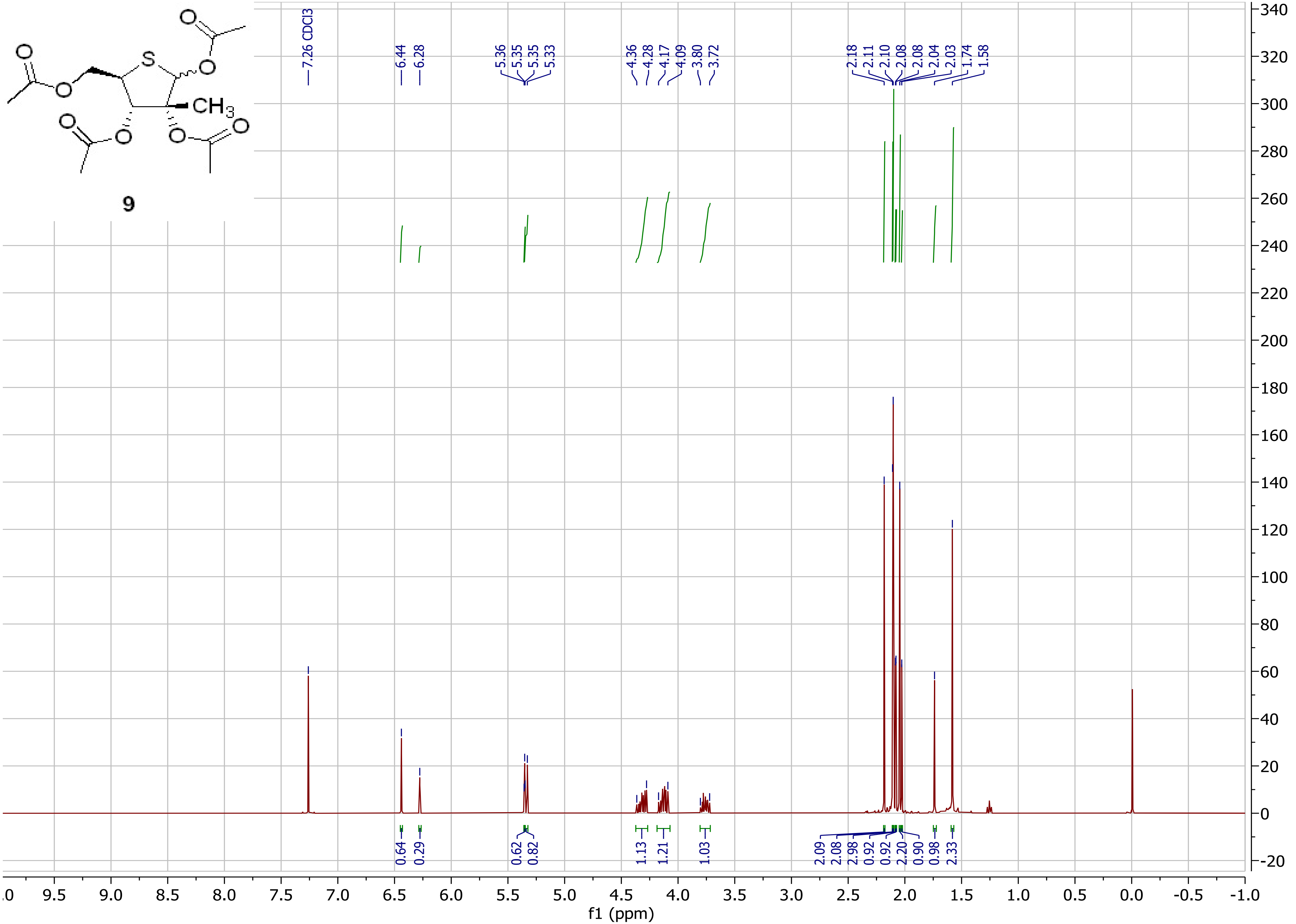

S
CH3
9
7.26 CDCl3
6.44
6.28
5.36
5.35
5.35
5.33
4.36
4.28
4.17
4.09
3.80
3.72
2.18
2.11
2.10
2.08
2.08
2.04
2.03
1.74
1.58
0.64
0.29
0.62
0.82
1.13
1.21
1.03
2.09
2.08
2.98
0.92
0.92
2.20
0.90
0.98
2.33
f1 (ppm)

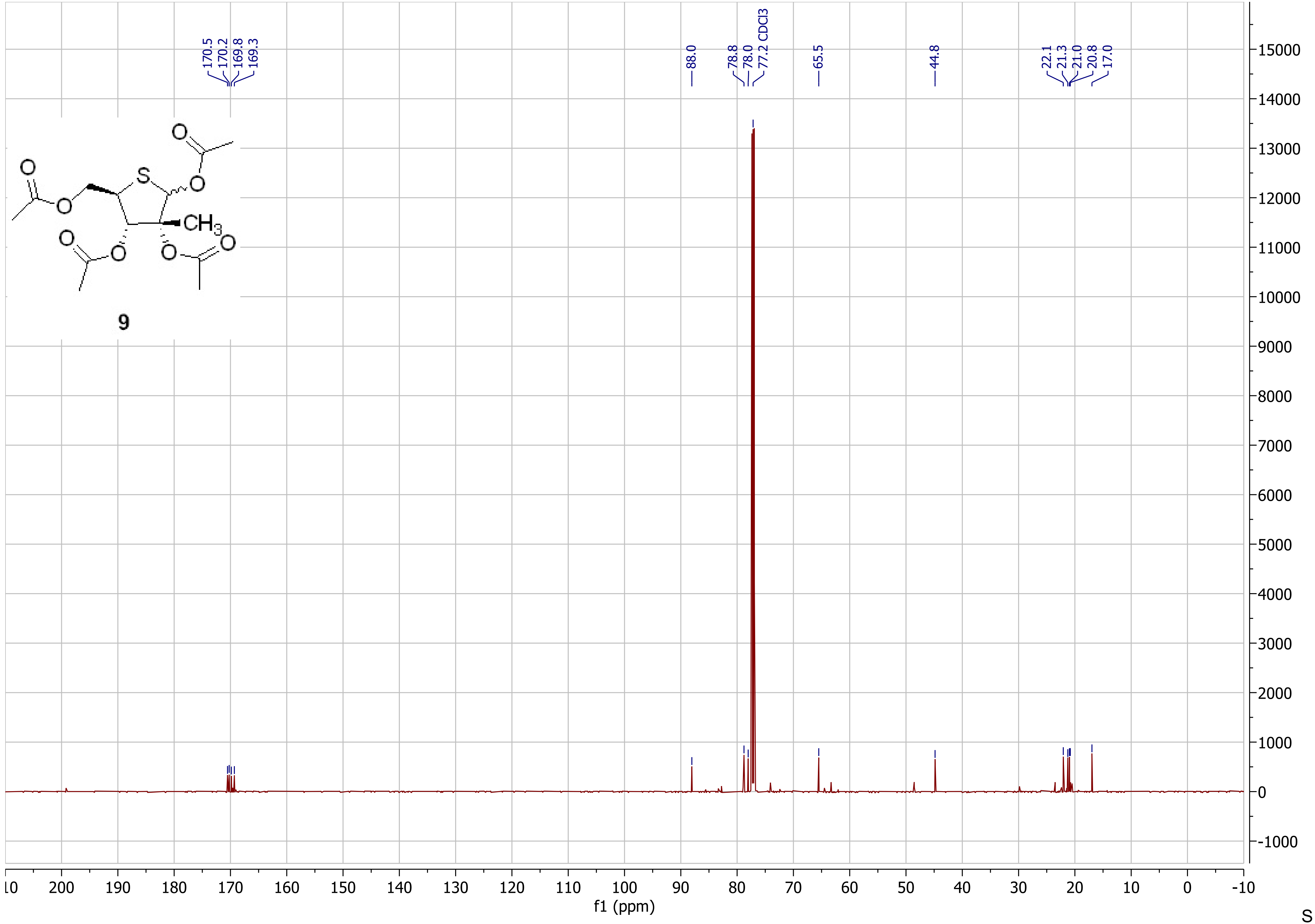
170.5
170.2
169.8
169.3
88.0
78.8
78.0
77.2 CDCl3
65.5
44.8
22.1
21.3
21.0
20.8
17.0
f1 (ppm)

9

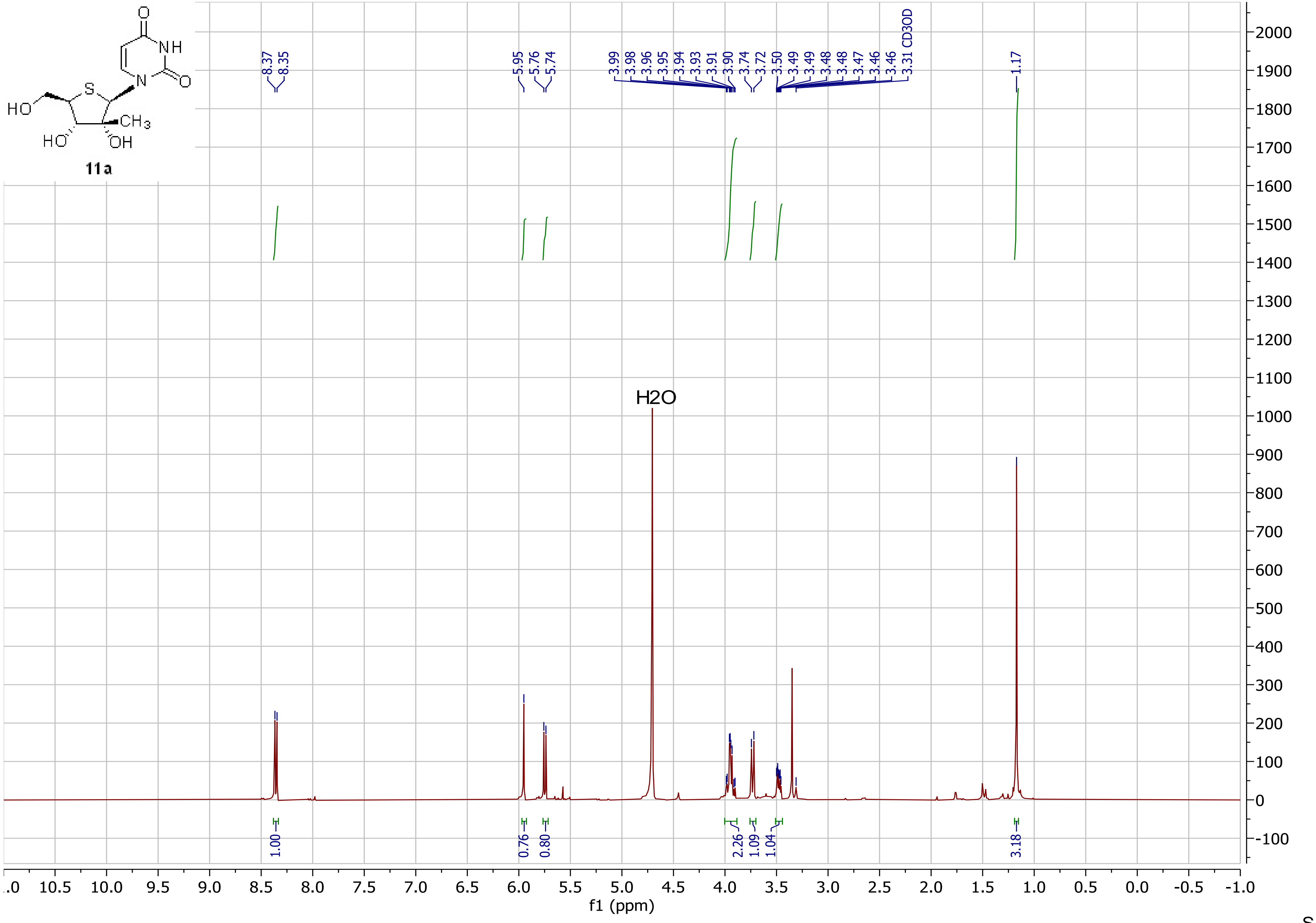
11a
8.37
8.35
5.95
5.76
5.74
3.99
3.98
3.96
3.95
3.94
3.93
3.91
3.90
3.74
3.72
3.50
3.49
3.49
3.48
3.48
3.47
3.46
3.46
3.31 CD3OD
1.17
H2O
1.00
0.76
0.80
2.26
1.09
1.04
3.18
f1 (ppm)

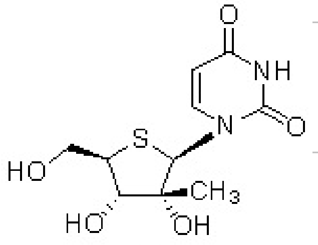

11a

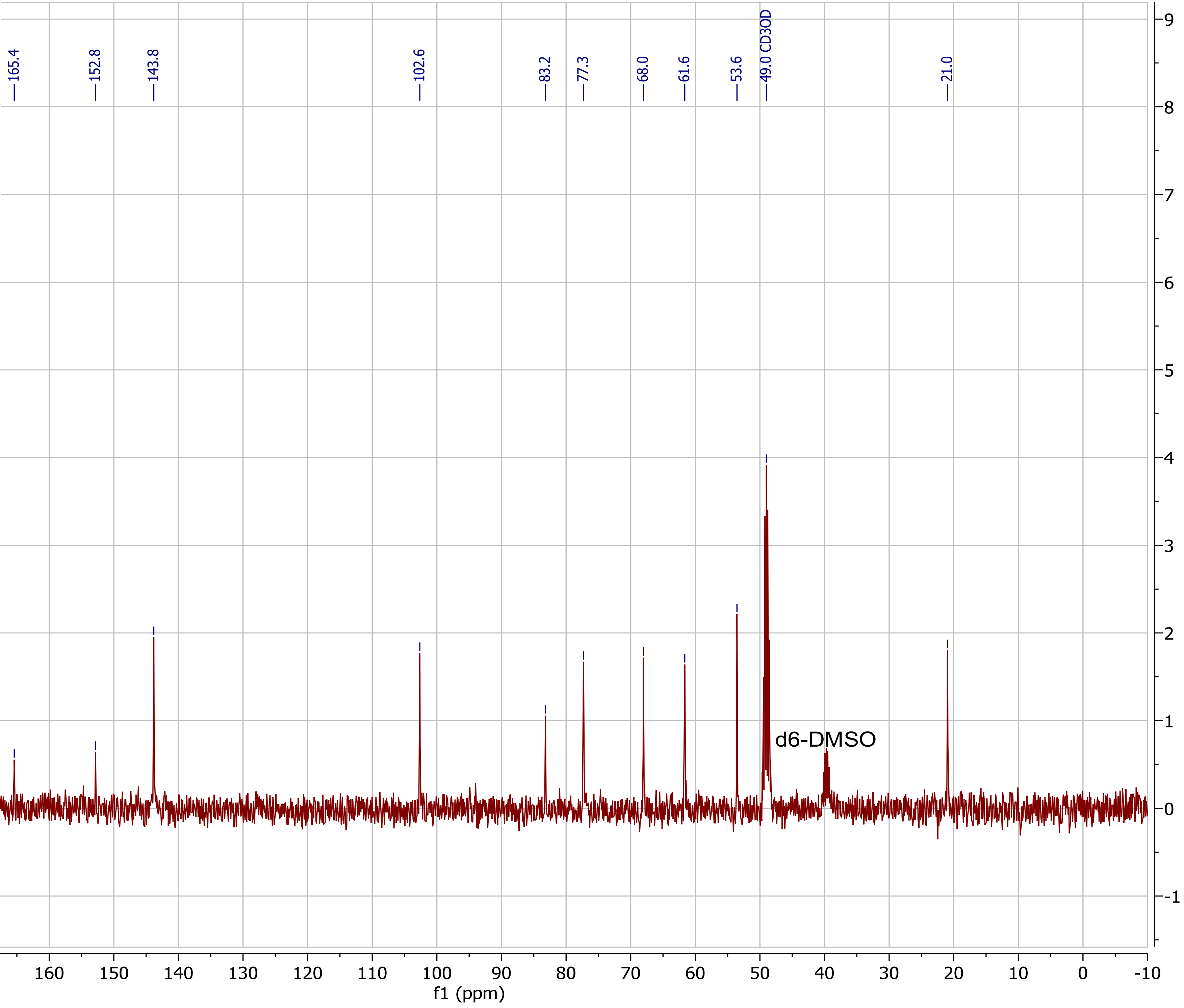

165.4
152.8
143.8
102.6
83.2
77.3
68.0
61.6
53.6
49.0 CD3OD
21.0
d6-DMSO
f1 (ppm)

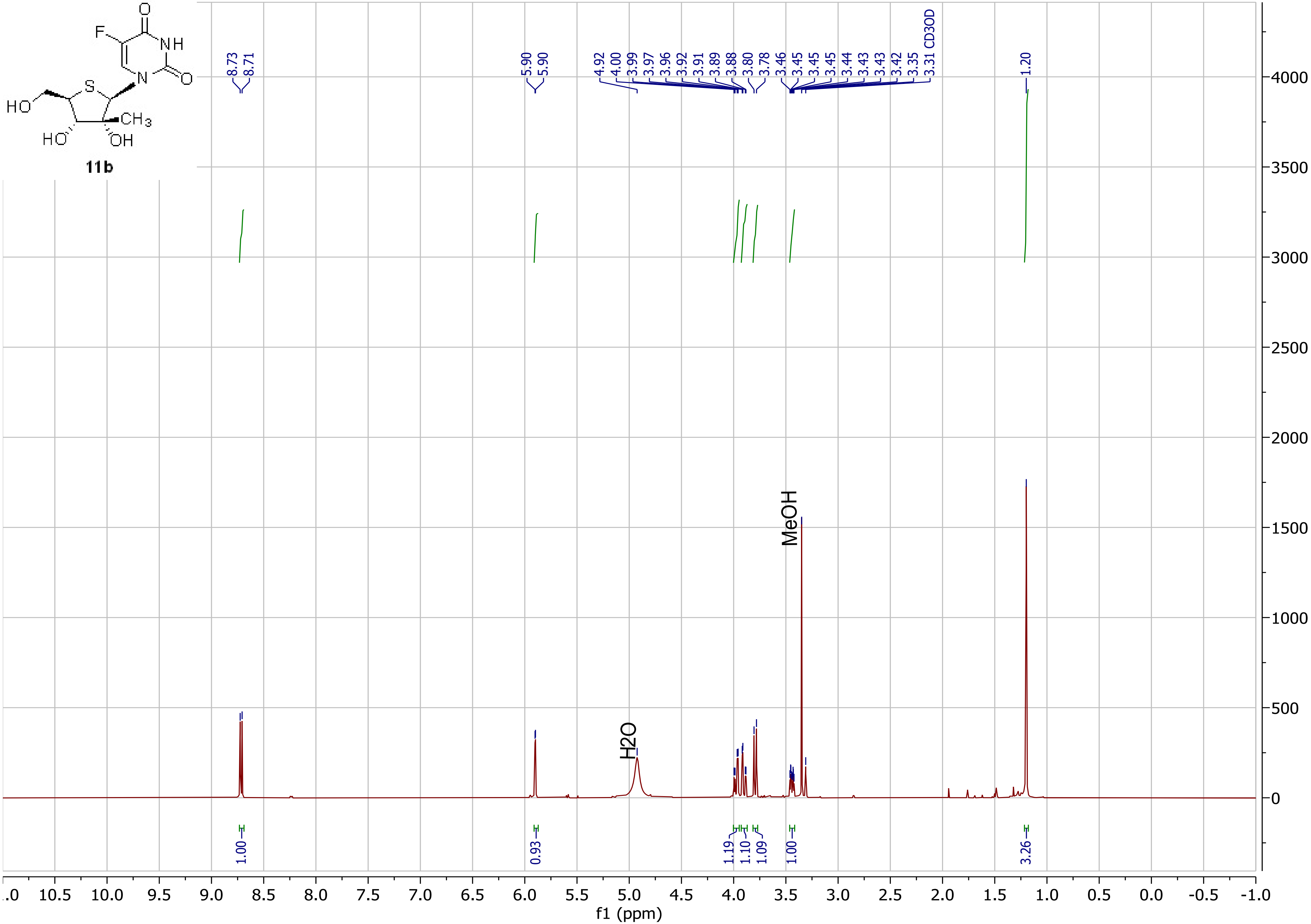
11b
8.73
8.71
5.90
5.90
4.92
4.00
3.99
3.97
3.96
3.92
3.91
3.89
3.88
3.80
3.78
3.46
3.45
3.45
3.45
3.44
3.43
3.43
3.42
3.35
3.31 CD3OD
1.20
H2O
MeOH
1.00
0.93
1.19
1.10
1.09
1.00
3.26
f1 (ppm)

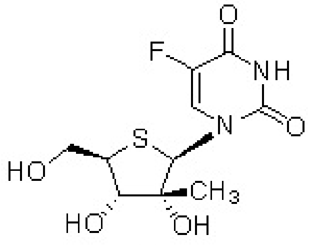
F
NH
O
N
S
HO
CH3
HO
OH

11b

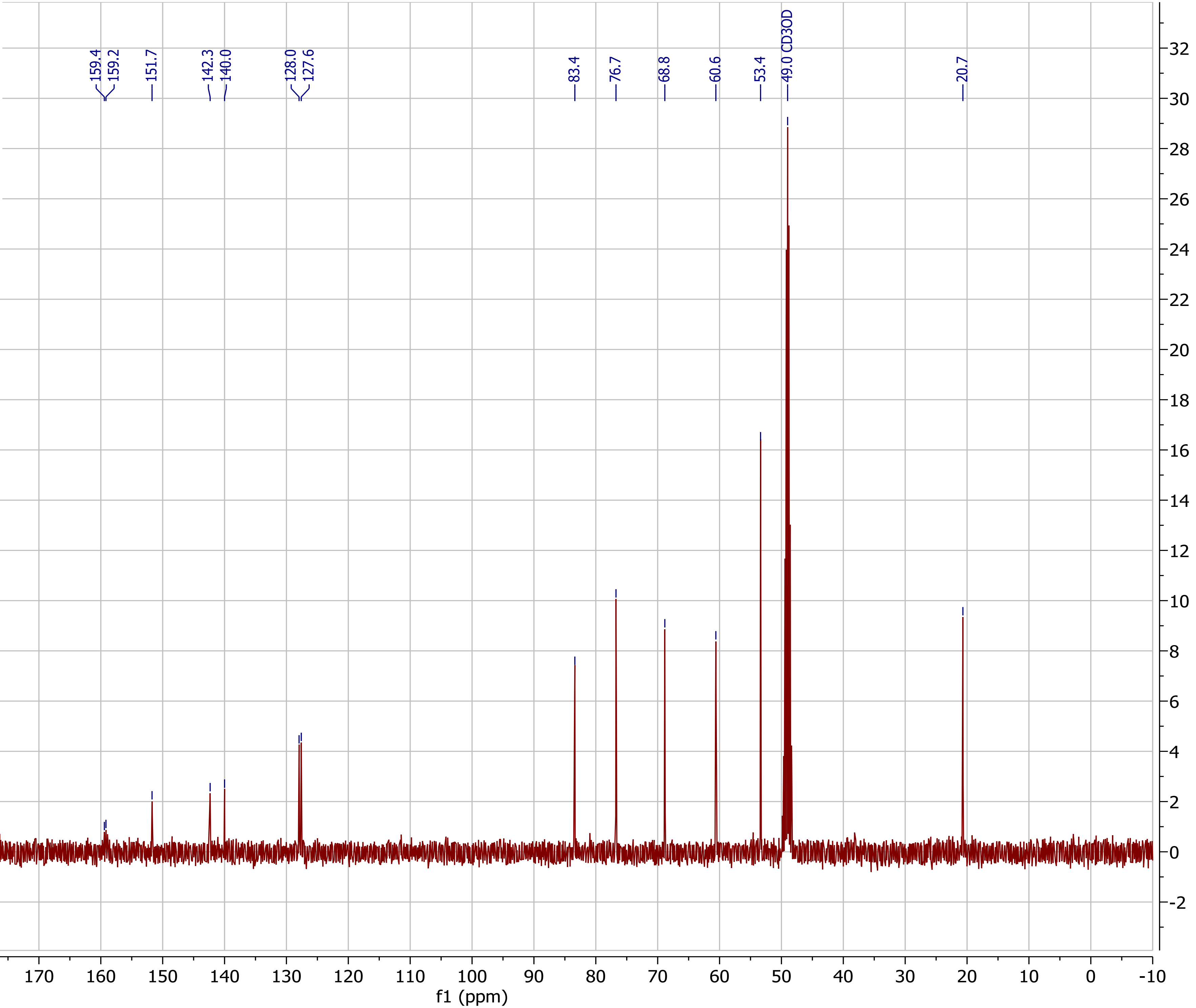
159.4
159.2
151.7
142.3
140.0
128.0
127.6
83.4
76.7
68.8
60.6
53.4
49.0 CD3OD
20.7
f1 (ppm)

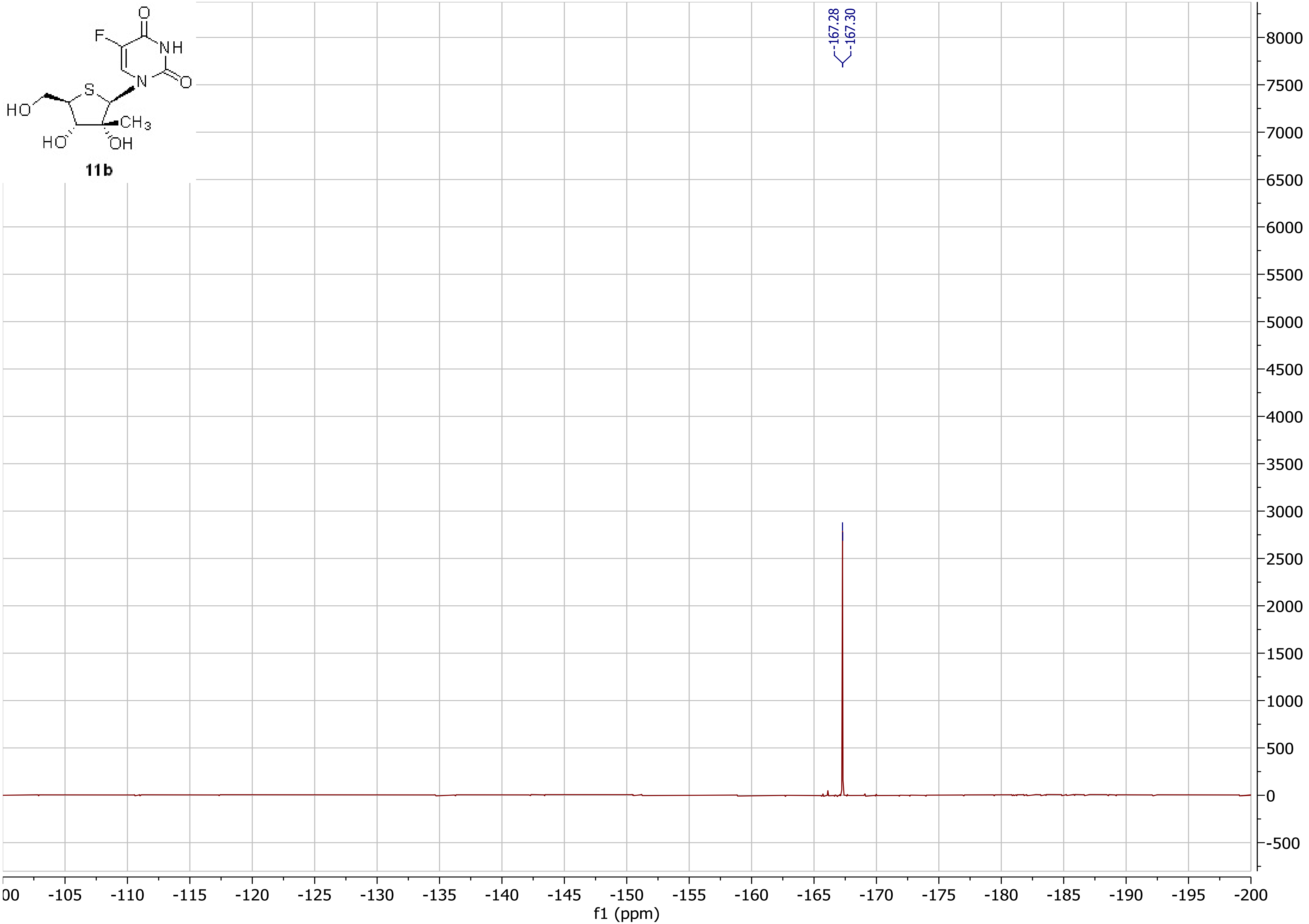

11b
-167.28
-167.30
f1 (ppm)

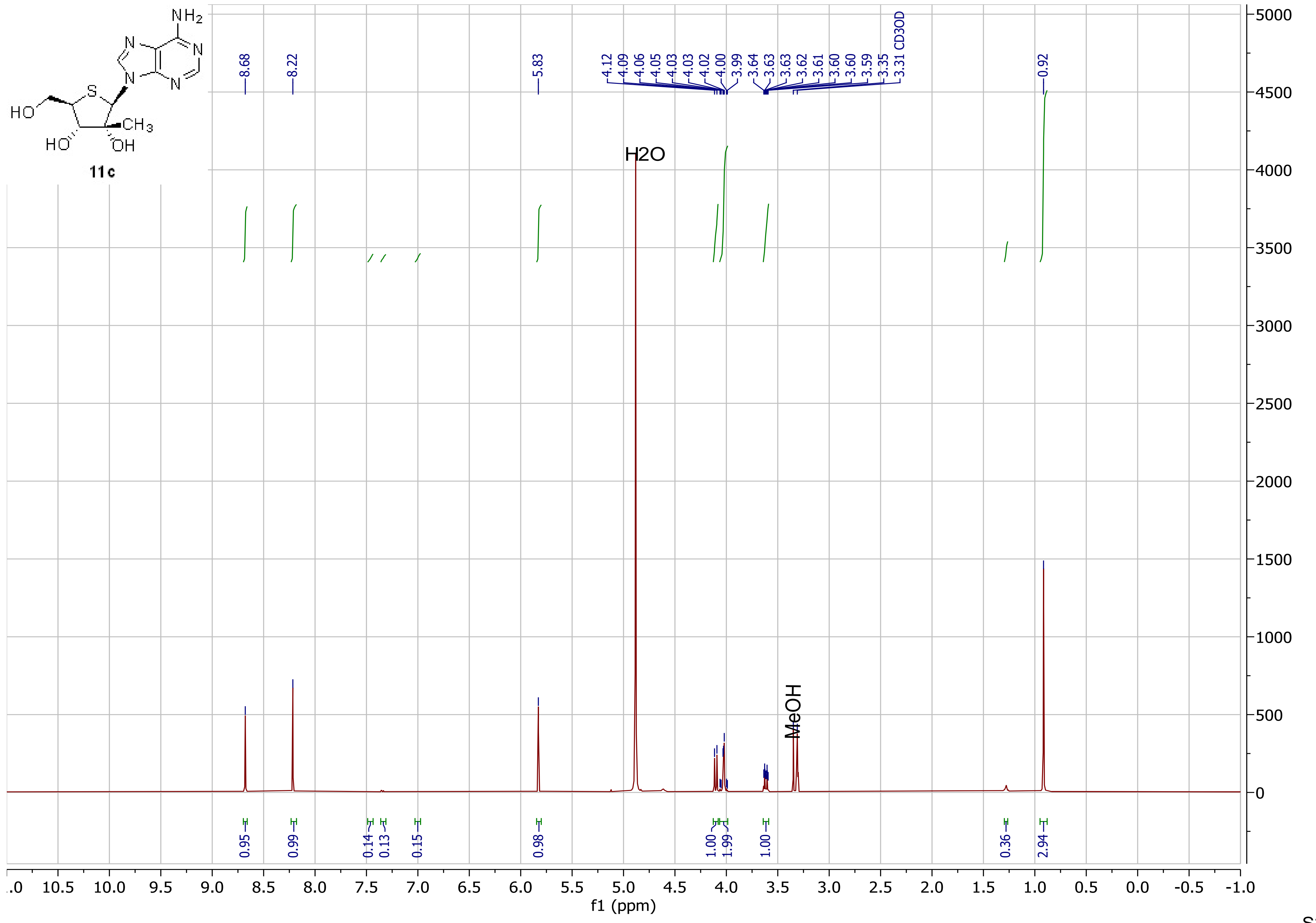
11c
H2O
MeOH
f1 (ppm)

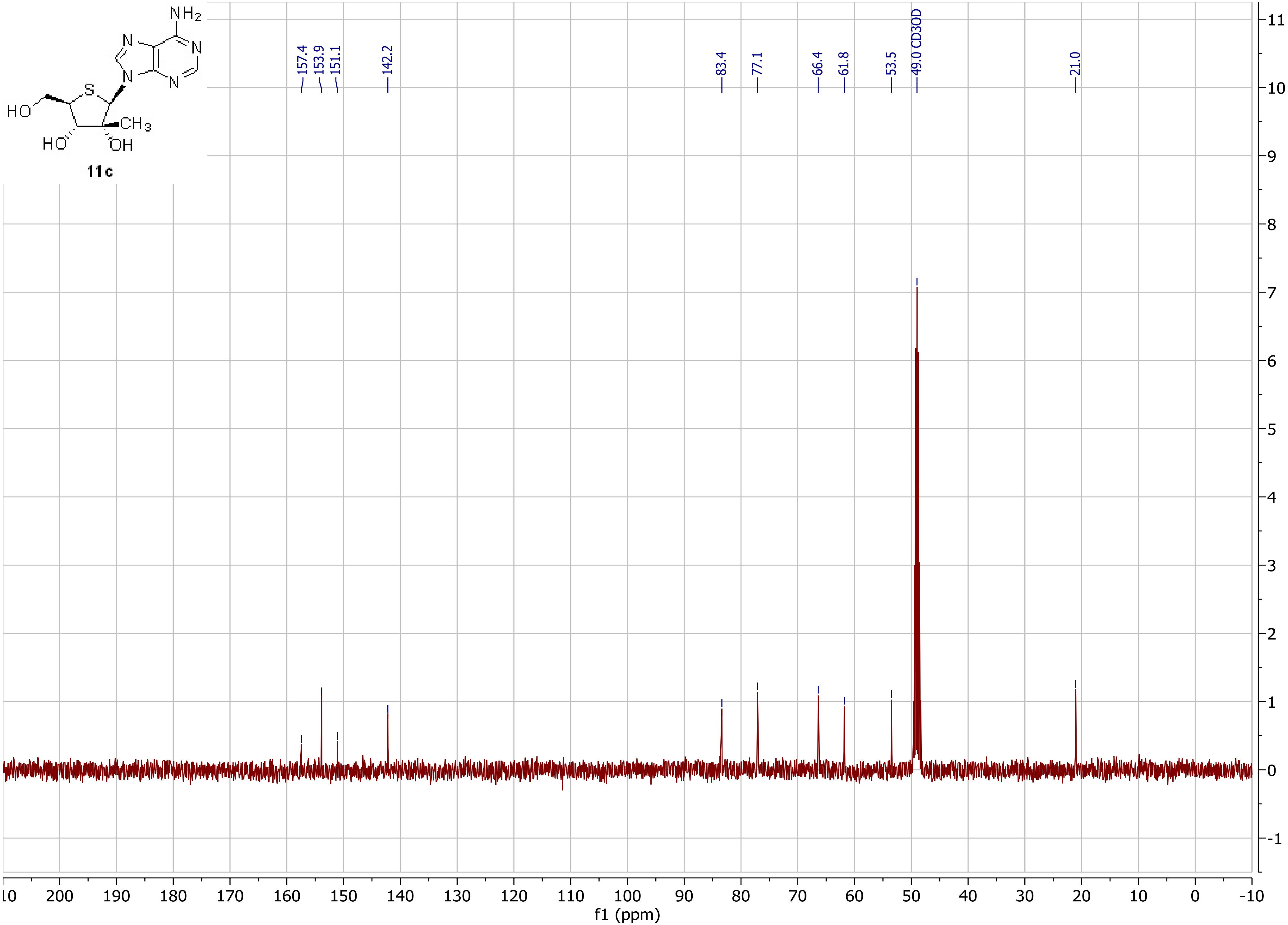
NH2
N
N
N
N
S
HO
HO
OH
CH3
11c
157.4
153.9
151.1
142.2
83.4
77.1
66.4
61.8
53.5
49.0 CD3OD
21.0
f1 (ppm)

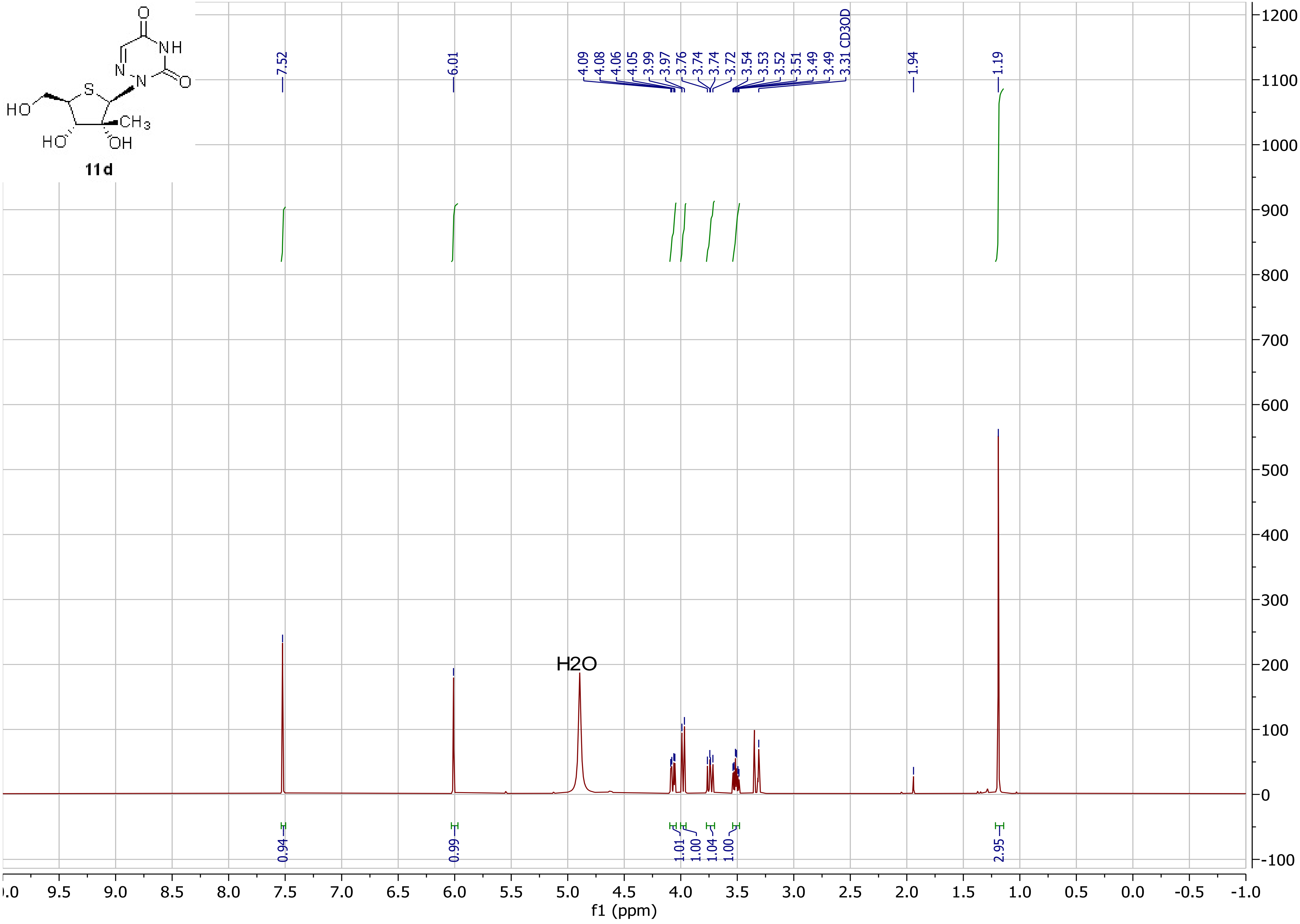

HO
HO
OH
CH3
NH
11d
7.52
6.01
4.09
4.08
4.06
4.05
3.99
3.97
3.76
3.74
3.74
3.72
3.54
3.53
3.52
3.51
3.49
3.49
3.31 CD3OD
1.94
1.19
H2O
0.94
0.99
1.01
1.00
1.04
1.00
2.95
f1 (ppm)

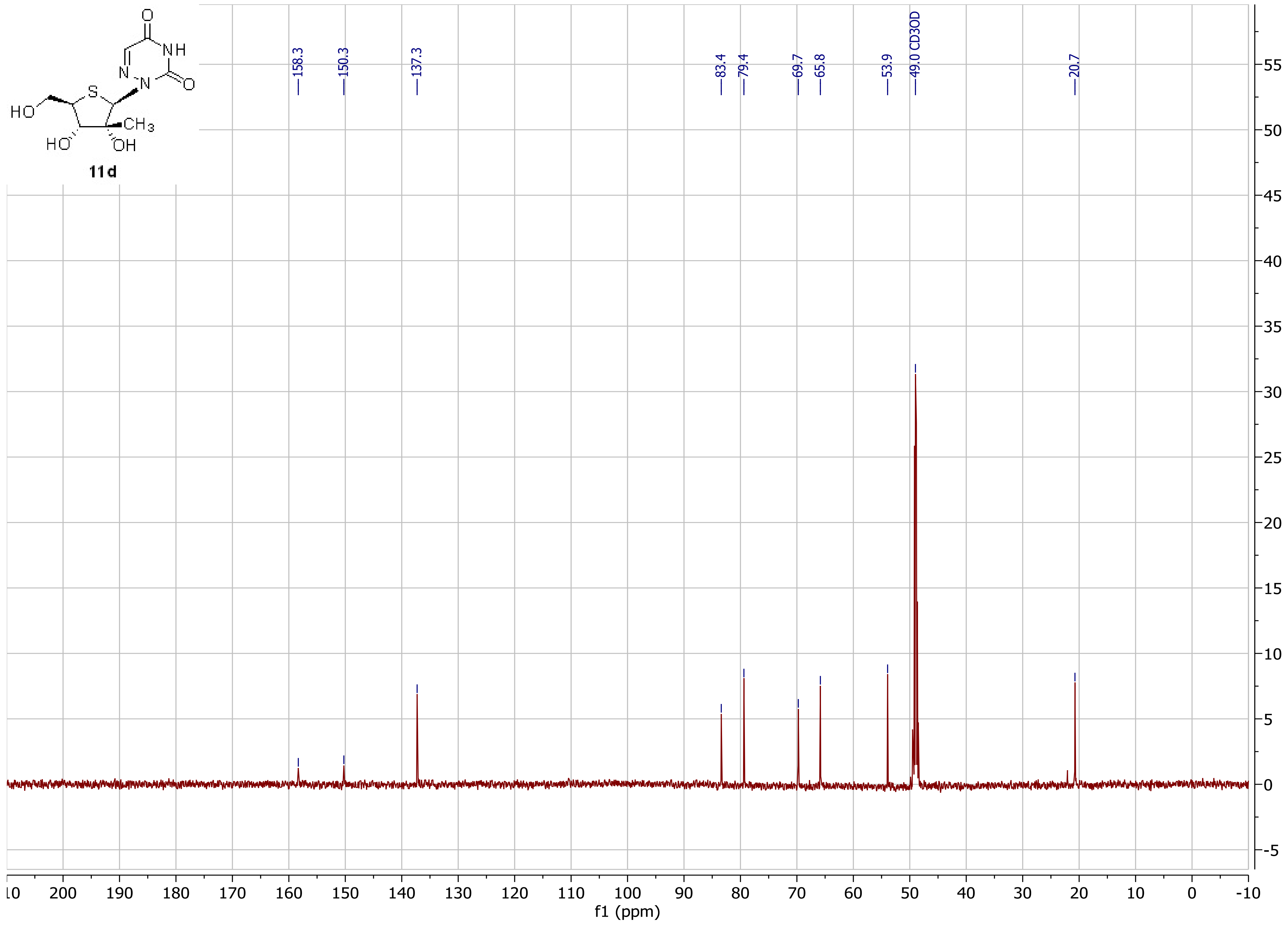
11d
158.3
150.3
137.3
83.4
79.4
69.7
65.8
53.9
49.0 CD3OD
20.7
f1 (ppm)

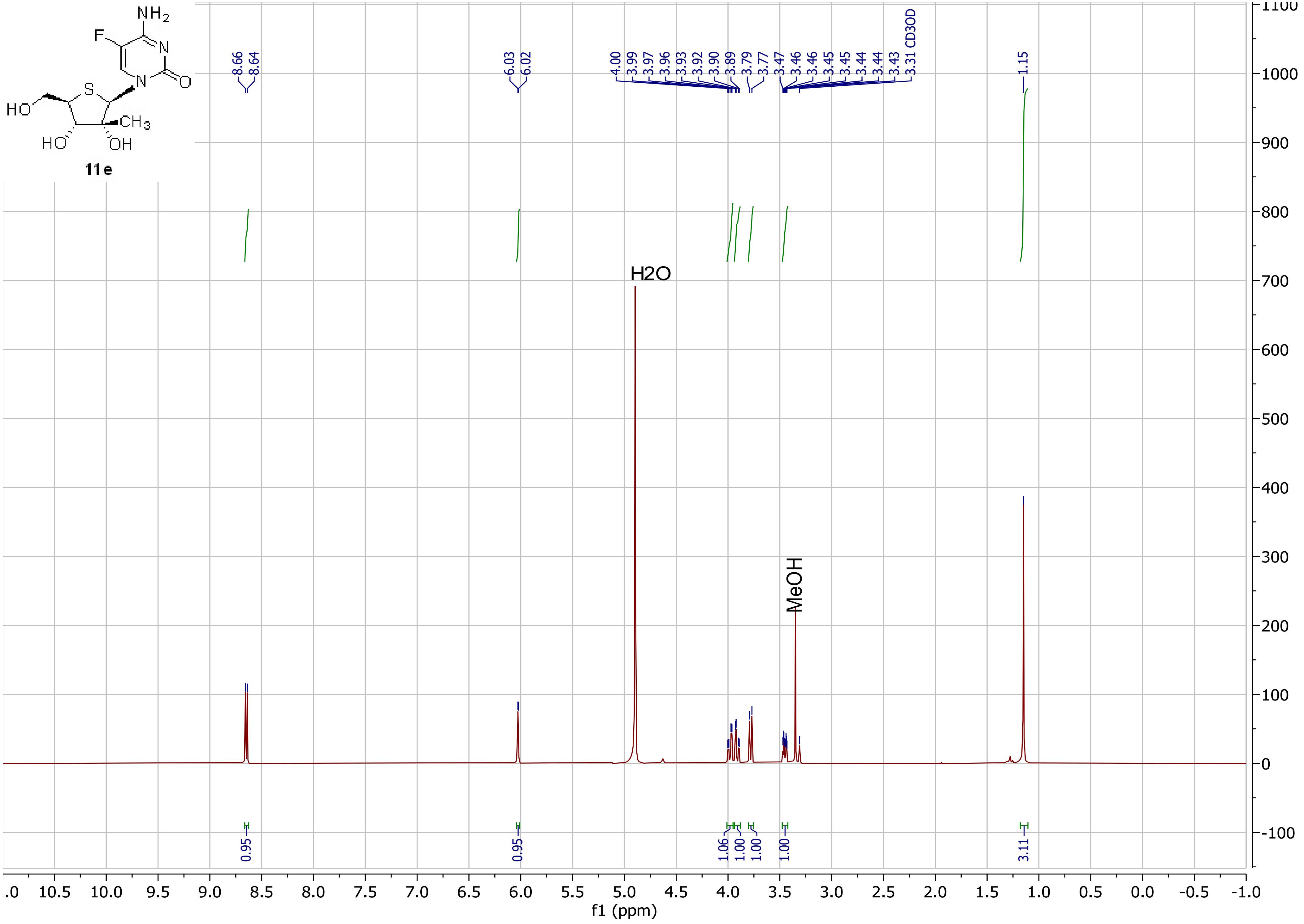
NH2
F
N
N
O
S
HO
CH3
HO
OH
11e
8.66
8.64
6.03
6.02
4.00
3.99
3.97
3.96
3.93
3.92
3.90
3.89
3.79
3.77
3.47
3.46
3.46
3.45
3.45
3.44
3.44
3.43
3.31 CD3OD
1.15
H2O
MeOH
0.95
0.95
1.06
1.00
1.00
1.00
3.11
f1 (ppm)

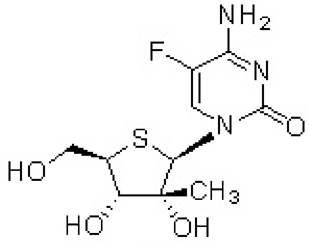

11e

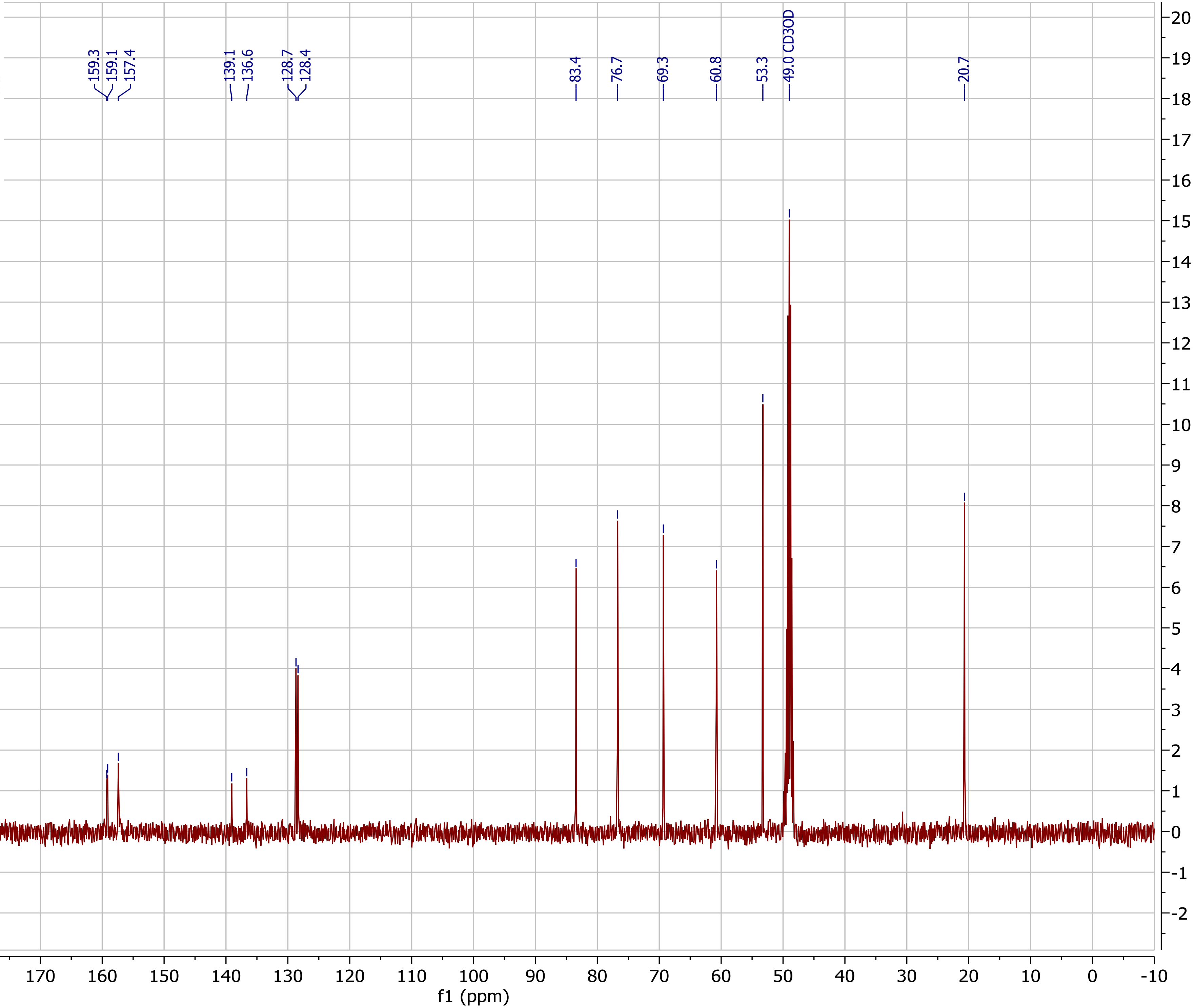
159.3
159.1
157.4
139.1
136.6
128.7
128.4
83.4
76.7
69.3
60.8
53.3
49.0 CD3OD
20.7
f1 (ppm)

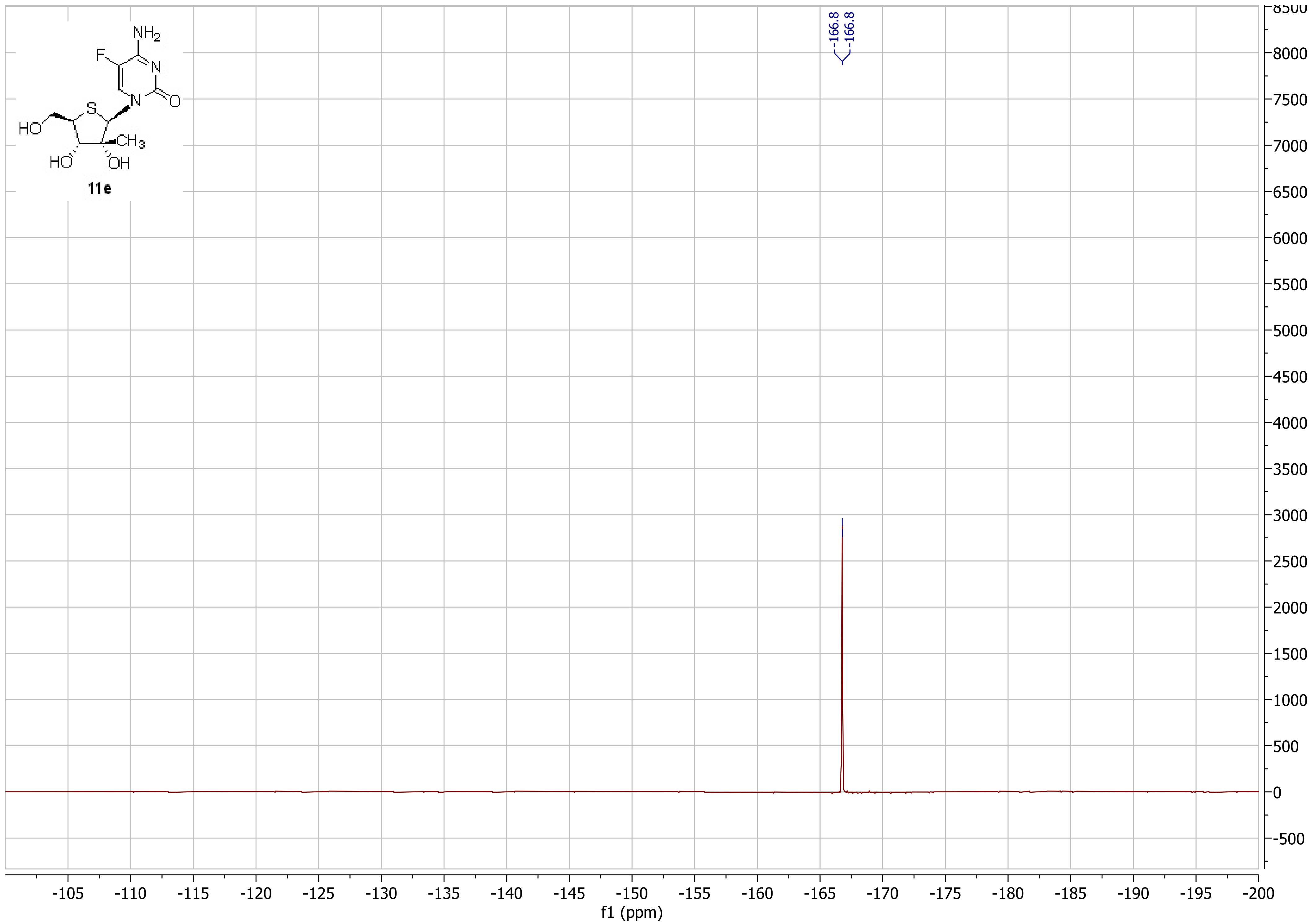

NH2
F
N
N
O
S
HO
CH3
HO
OH
11e
-166.8
-166.8
8500
8000
7500
7000
6500
6000
5500
5000
4500
4000
3500
3000
2500
2000
1500
1000
500
0
-500
-105 -110 -115 -120 -125 -130 -135 -140 -145 -150 -155 -160 -165 -170 -175 -180 -185 -190 -195 -200
f1 (ppm)

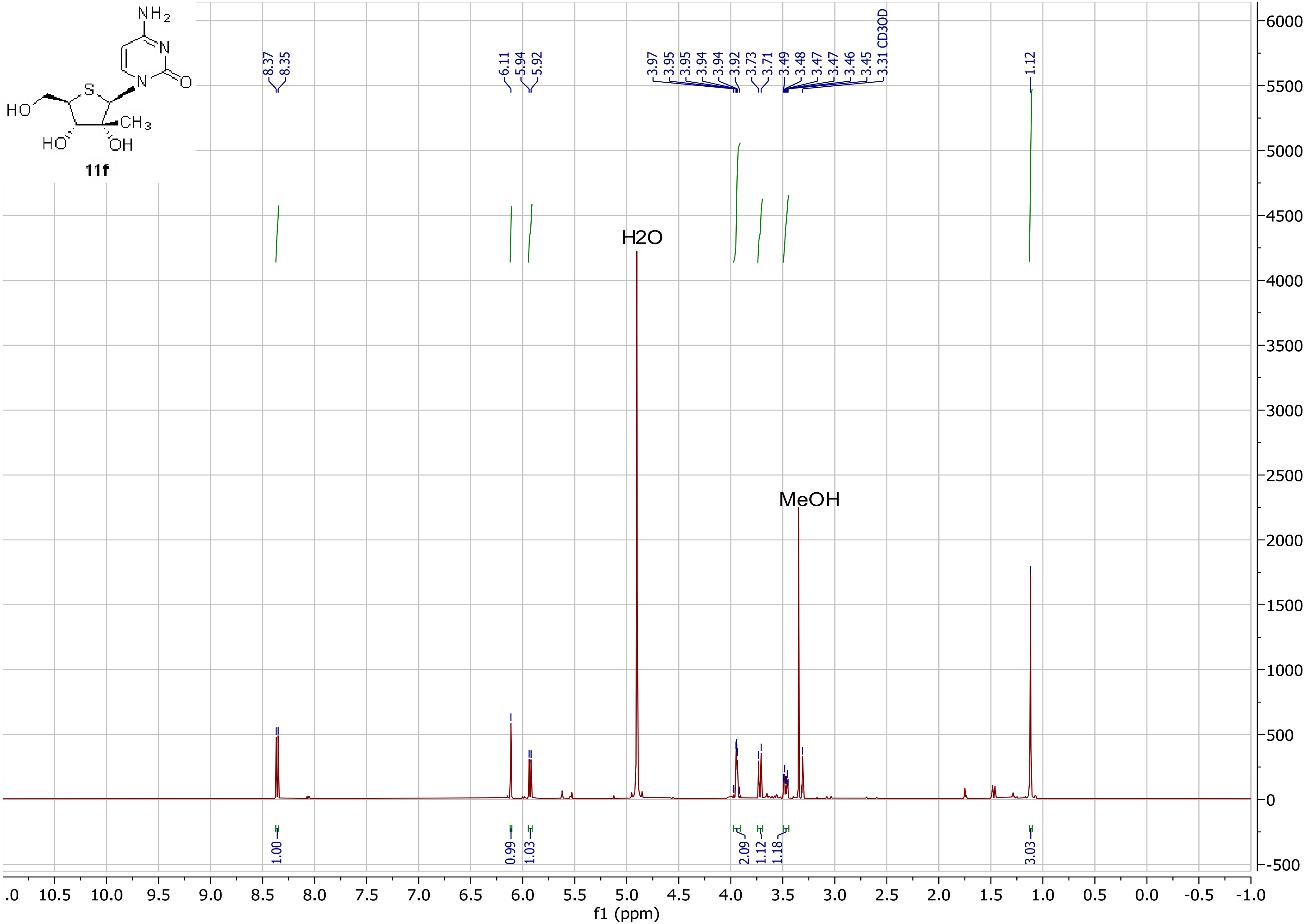

NH2
N
O
N
S
HO
CH3
HO
OH
11f
8.37
8.35
6.11
5.94
5.92
3.97
3.95
3.95
3.94
3.94
3.92
3.73
3.71
3.49
3.48
3.47
3.47
3.46
3.45
3.31 CD3OD
1.12
H2O
MeOH
1.00
0.99
1.03
2.09
1.12
1.18
3.03
f1 (ppm)

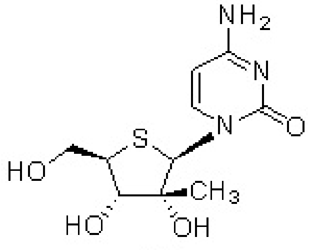

11f

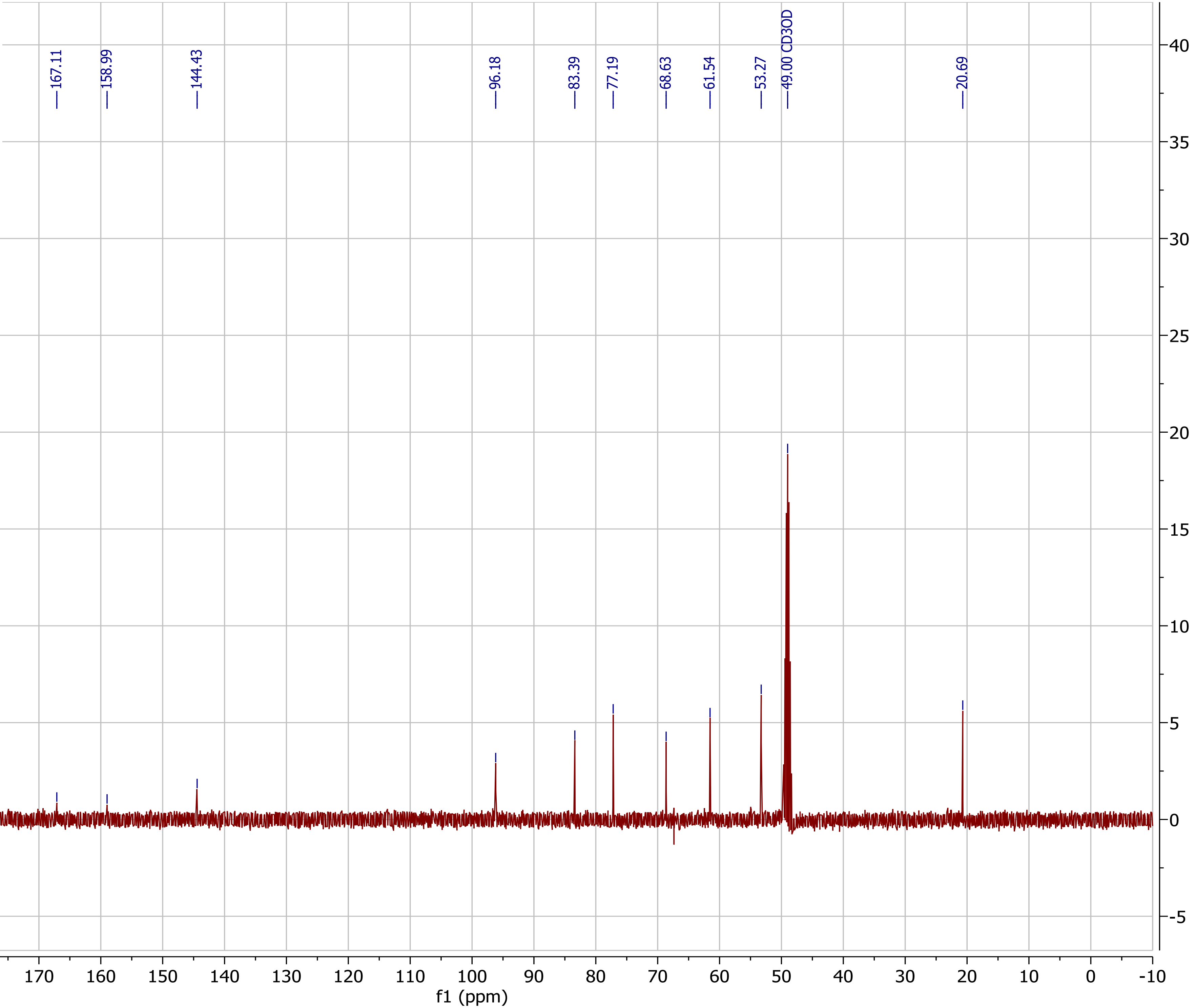

167.11
158.99
144.43
96.18
83.39
77.19
68.63
61.54
53.27
49.00 CD3OD
20.69
f1 (ppm)

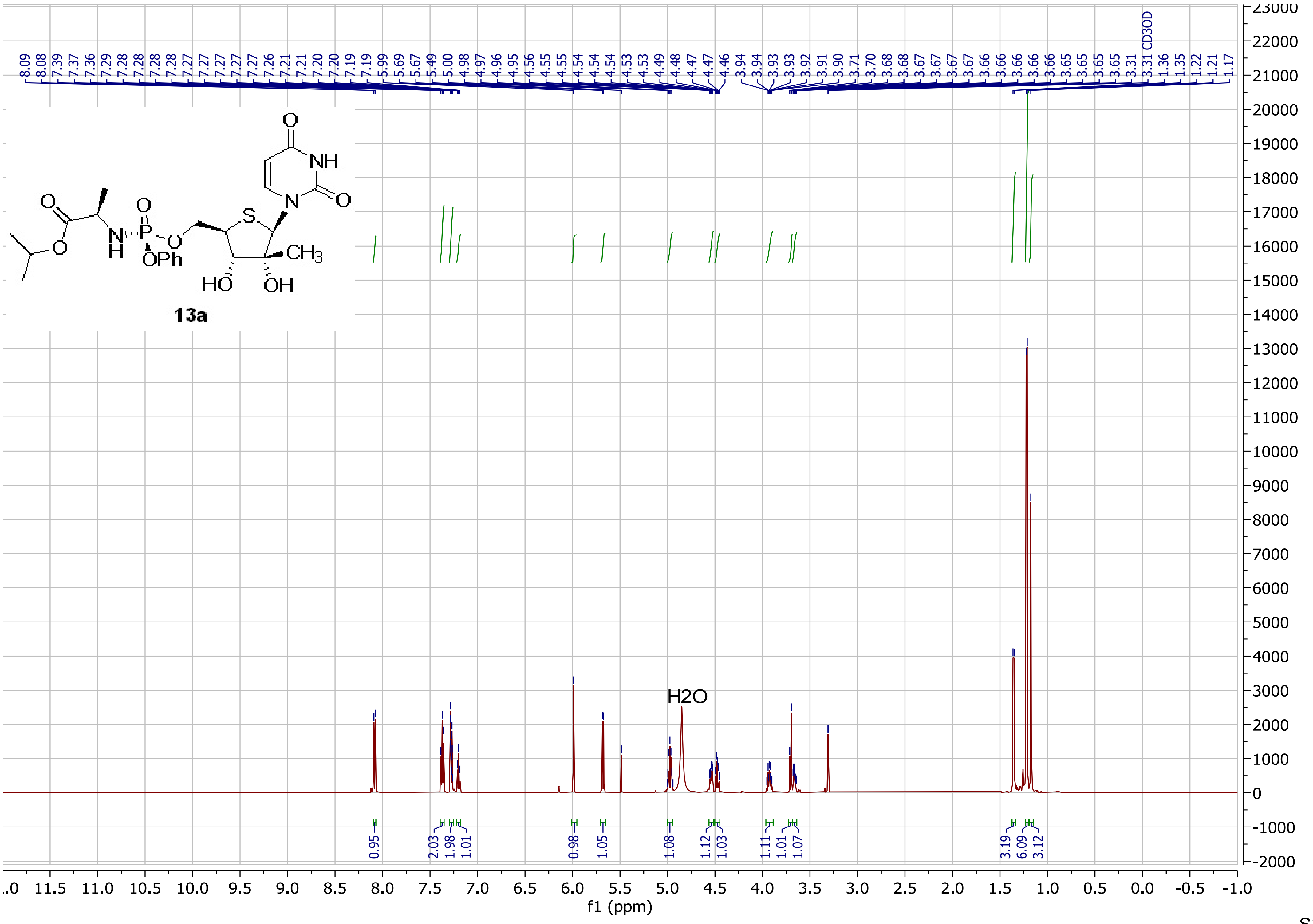

8.09 8.08 7.39 7.37 7.36 7.29 7.28 7.28 7.28 7.28 7.27 7.27 7.27 7.27 7.27 7.26 7.21 7.21 7.20 7.20 7.19 7.19 5.99 5.69 5.67 5.49 5.00 4.98 4.97 4.96 4.95 4.56 4.55 4.55 4.54 4.54 4.54 4.53 4.53 4.49 4.48 4.47 4.47 4.46 3.94 3.94 3.93 3.93 3.92 3.91 3.90 3.71 3.70 3.68 3.68 3.67 3.67 3.67 3.67 3.66 3.66 3.66 3.66 3.66 3.65 3.65 3.65 3.65 3.31 3.31 CD3OD 1.36 1.35 1.22 1.21 1.17
13a
H2O
0.95 2.03 1.98 1.01 0.98 1.05 1.08 1.12 1.03 1.11 1.01 1.07 3.19 6.09 3.12
f1 (ppm)

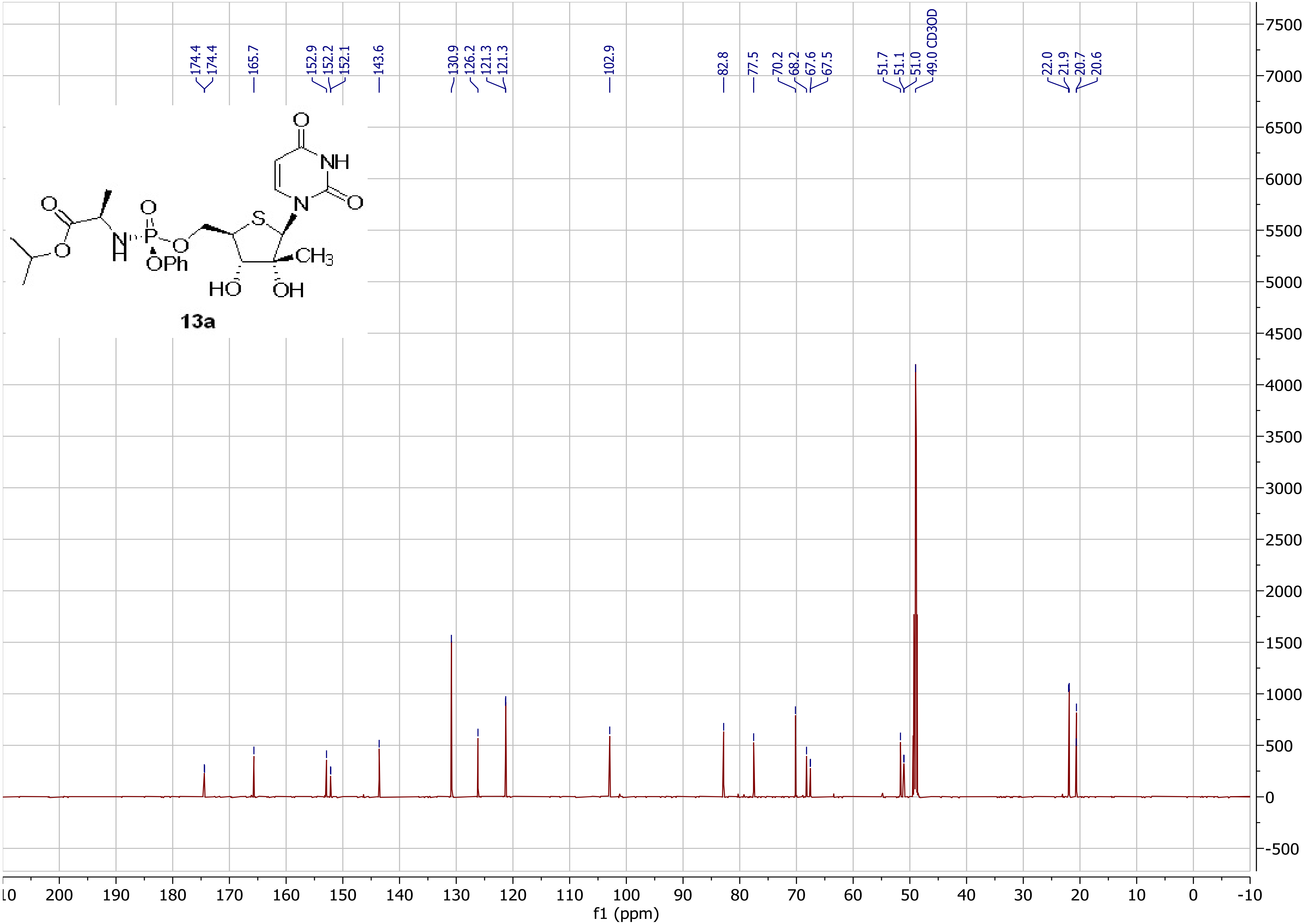

174.4
174.4
165.7
152.9
152.2
152.1
143.6
130.9
126.2
121.3
121.3
102.9
82.8
77.5
70.2
68.2
67.6
67.5
51.7
51.1
51.0
49.0 CD3OD
22.0
21.9
20.7
20.6
13a
f1 (ppm)

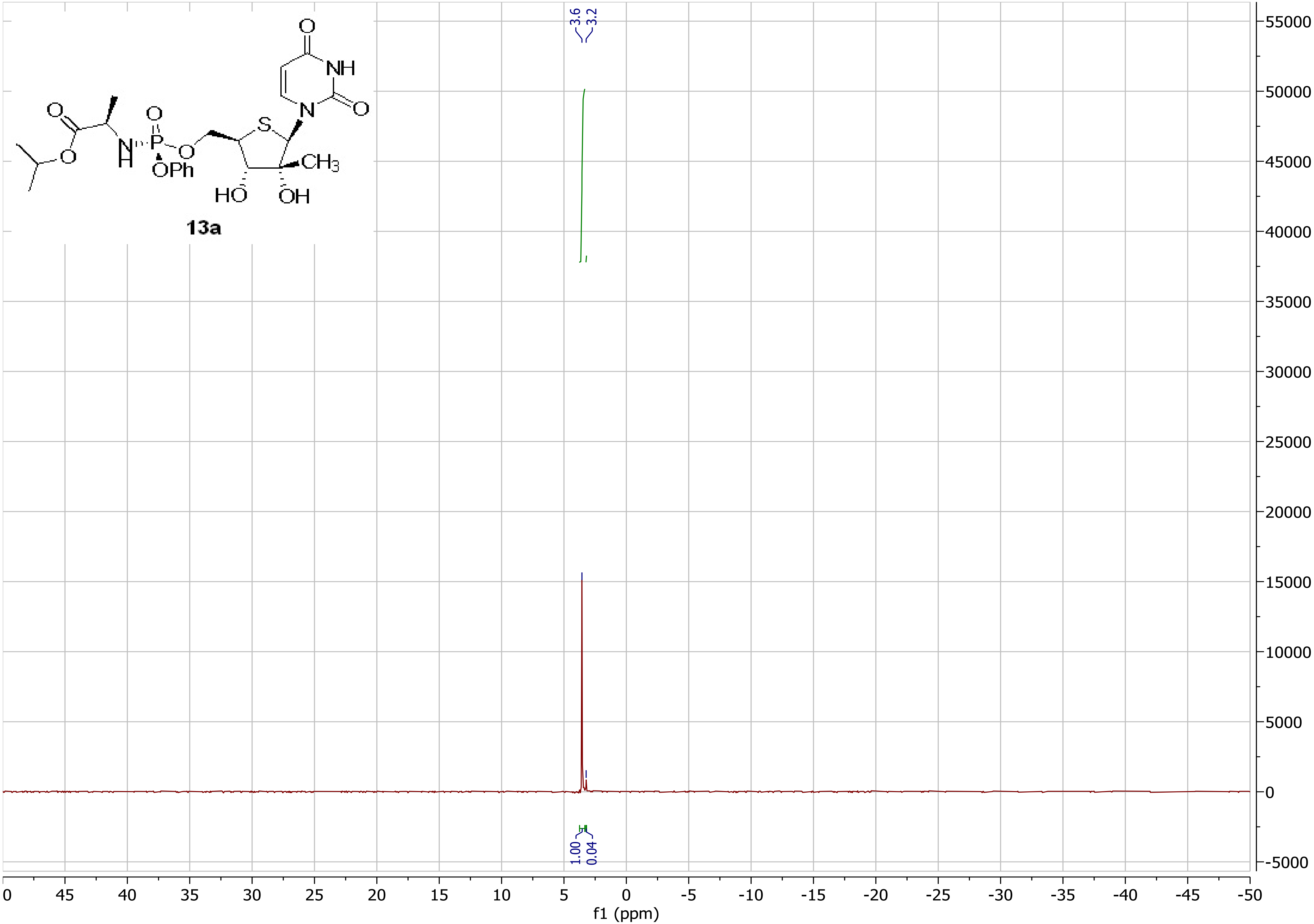
3.6
3.2
13a
NH
CH3
OPh
HO
OH
1.00
0.04
f1 (ppm)

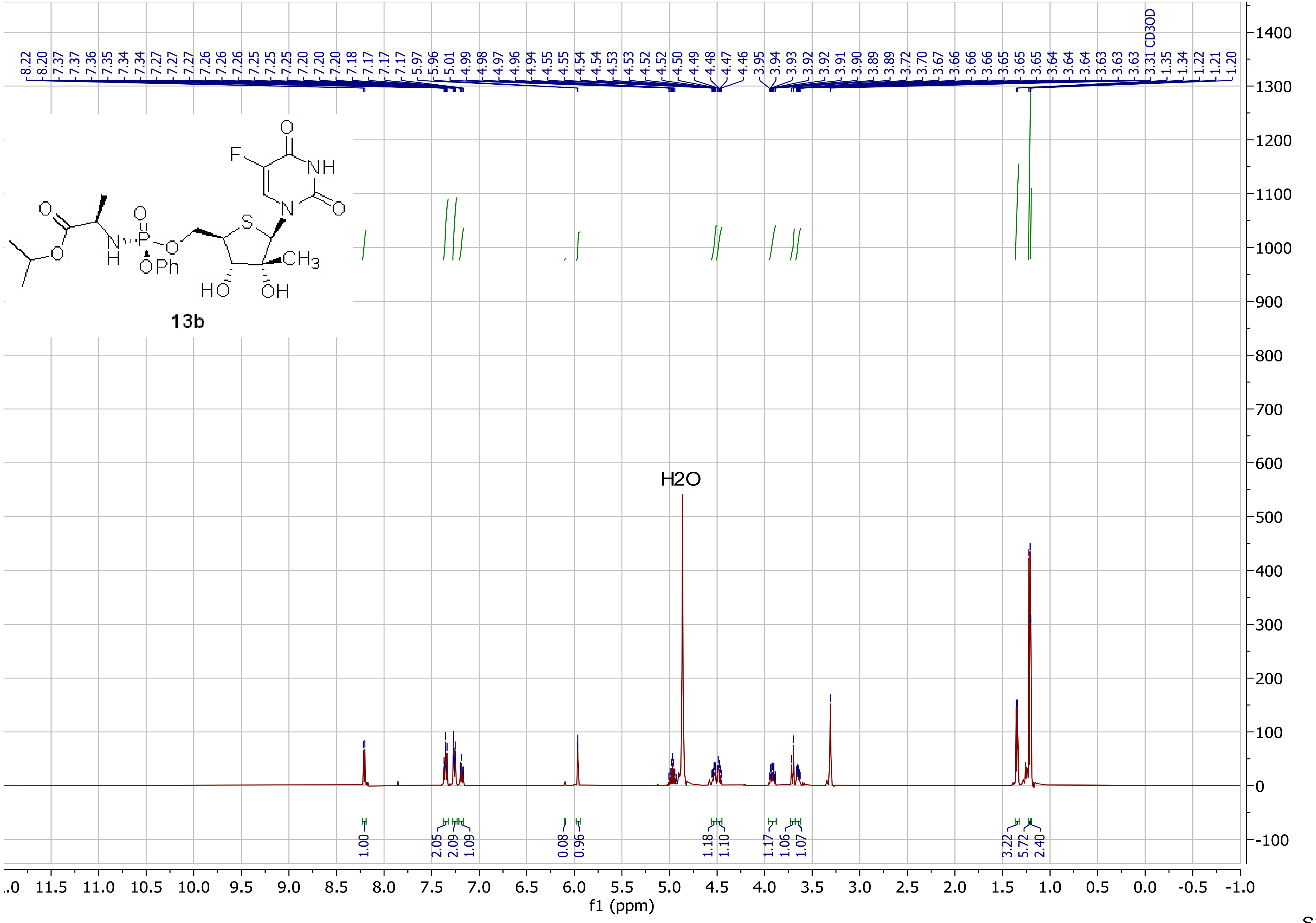
8.22 8.20 7.37 7.37 7.36 7.35 7.34 7.34 7.27 7.27 7.27 7.26 7.26 7.26 7.25 7.25 7.25 7.20 7.20 7.20 7.18 7.17 7.17 7.17 5.97 5.96 5.01 4.99 4.98 4.97 4.96 4.94 4.55 4.55 4.54 4.54 4.53 4.53 4.52 4.52 4.50 4.49 4.48 4.47 4.46 3.95 3.94 3.93 3.92 3.92 3.91 3.90 3.89 3.89 3.72 3.70 3.67 3.66 3.66 3.66 3.65 3.65 3.65 3.64 3.64 3.64 3.63 3.63 3.63 3.31 CD3OD 1.35 1.34 1.22 1.21 1.20
13b
H2O
1.00 2.05 2.09 1.09 0.08 0.96 1.18 1.10 1.17 1.06 1.07 3.22 5.72 2.40
f1 (ppm)

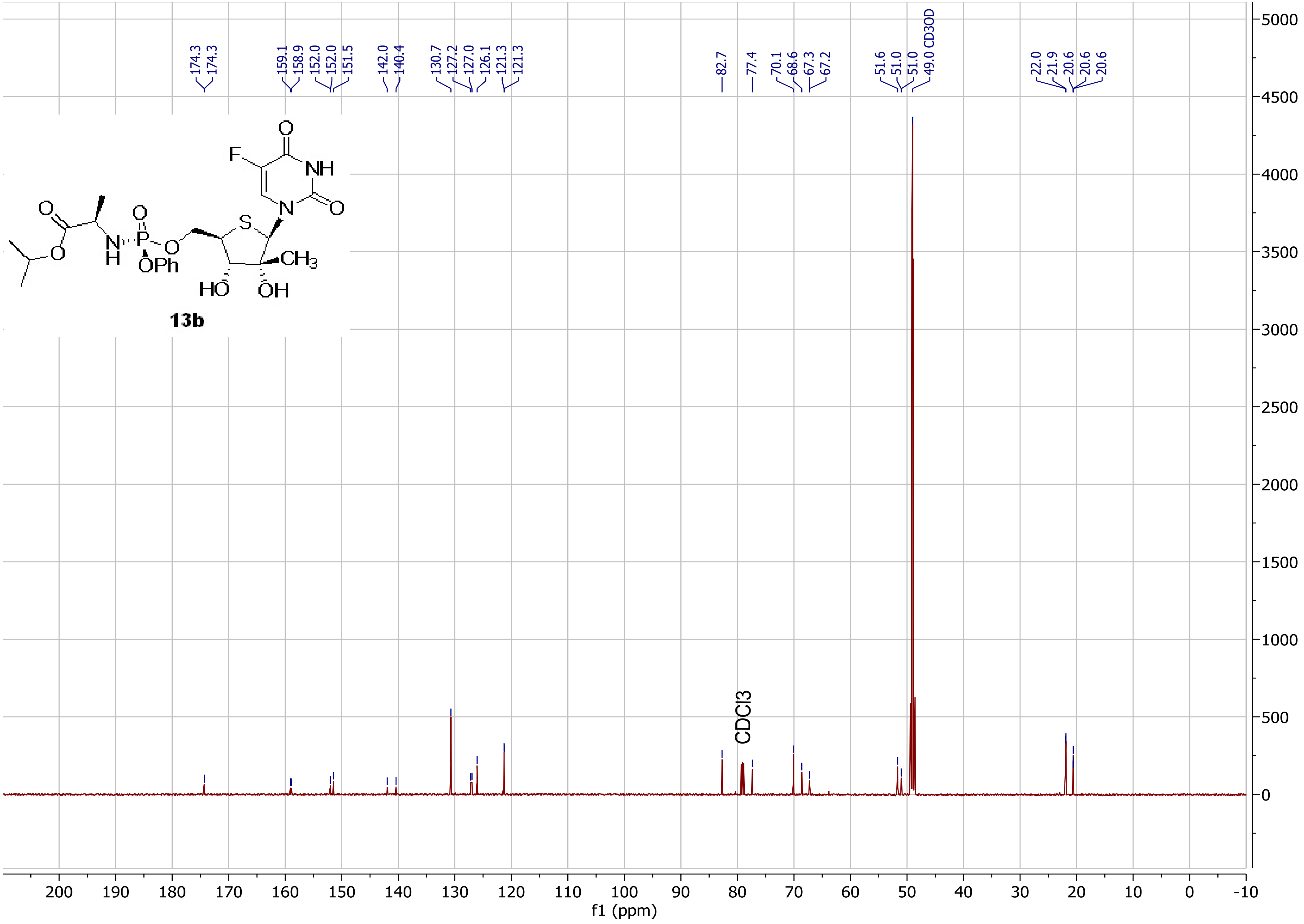

174.3
174.3
159.1
158.9
152.0
152.0
151.5
142.0
140.4
130.7
127.2
127.0
126.1
121.3
121.3
82.7
77.4
70.1
68.6
67.3
67.2
51.6
51.0
51.0
49.0 CD3OD
22.0
21.9
20.6
20.6
20.6
13b
CDCl3
f1 (ppm)

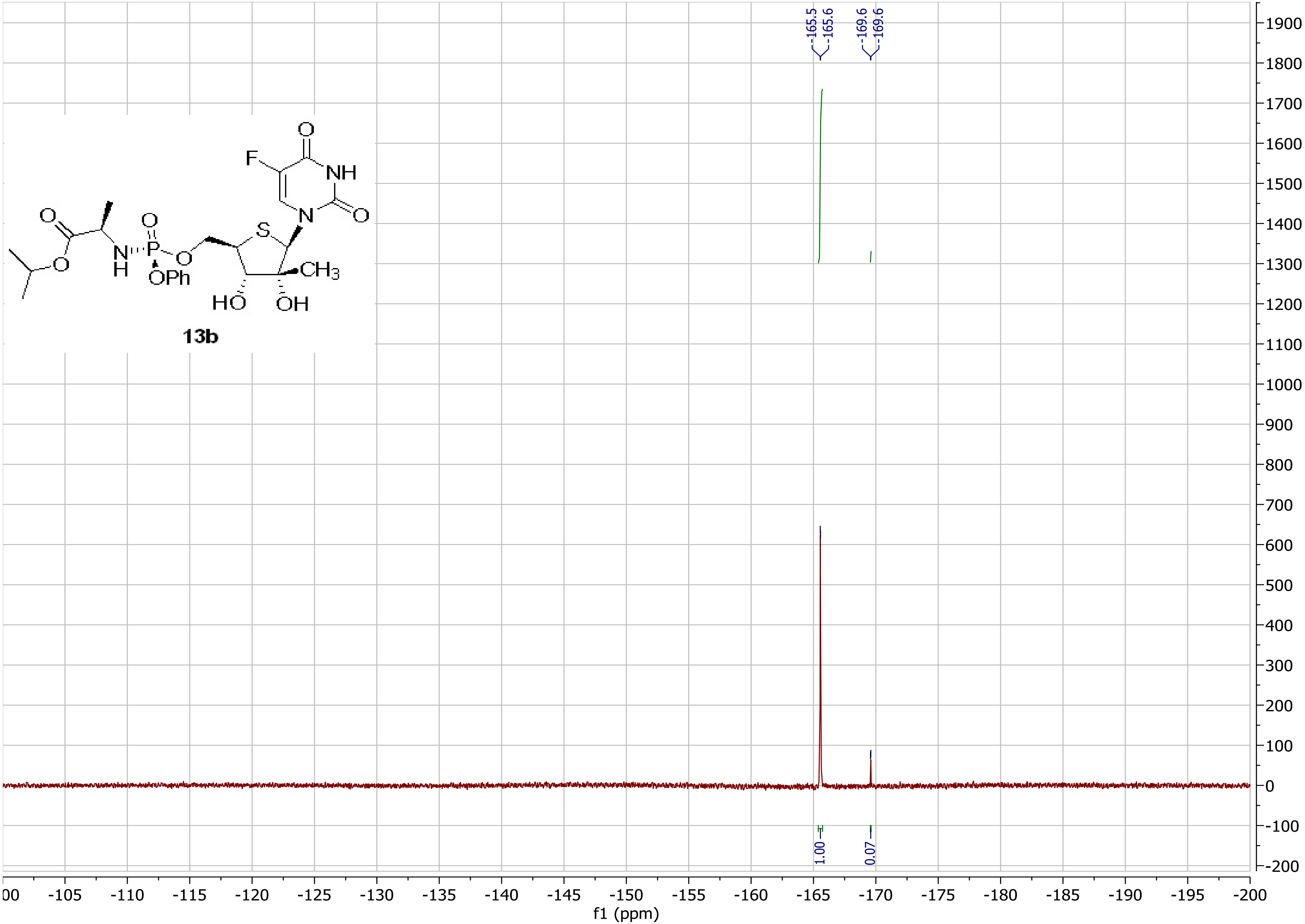

13b
-165.5
-165.6
-169.6
-169.6
1.00
0.07
f1 (ppm)

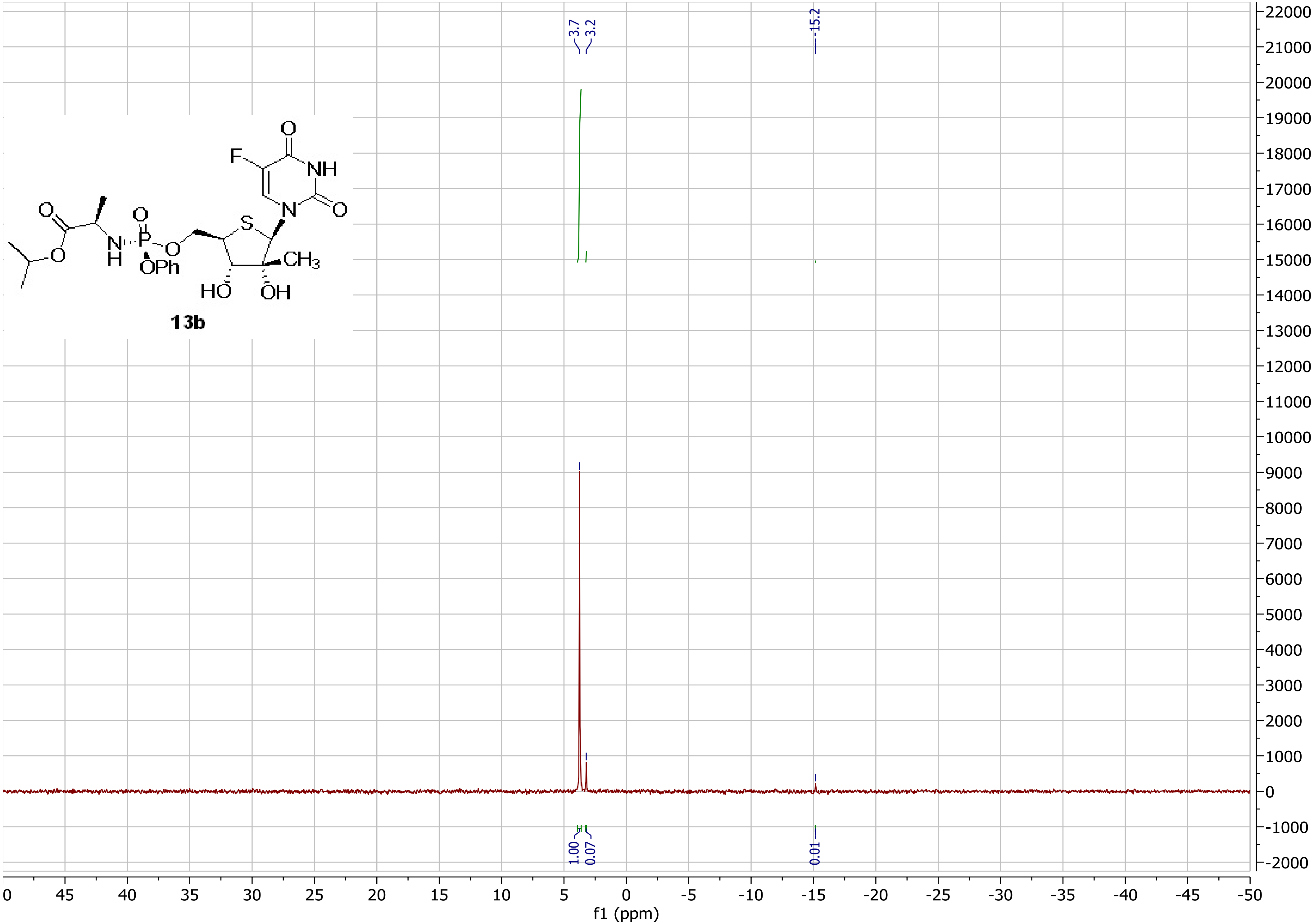

F
NH
O
O
N
S
O
O
P
N
H
OPh
O
O
CH3
HO
OH
13b
3.7
3.2
-15.2
1.00
0.07
0.01
22000
21000
20000
19000
18000
17000
16000
15000
14000
13000
12000
11000
10000
9000
8000
7000
6000
5000
4000
3000
2000
1000
0
-1000
-2000
0 45 40 35 30 25 20 15 10 5 0 -5 -10 -15 -20 -25 -30 -35 -40 -45 -50
f1 (ppm)

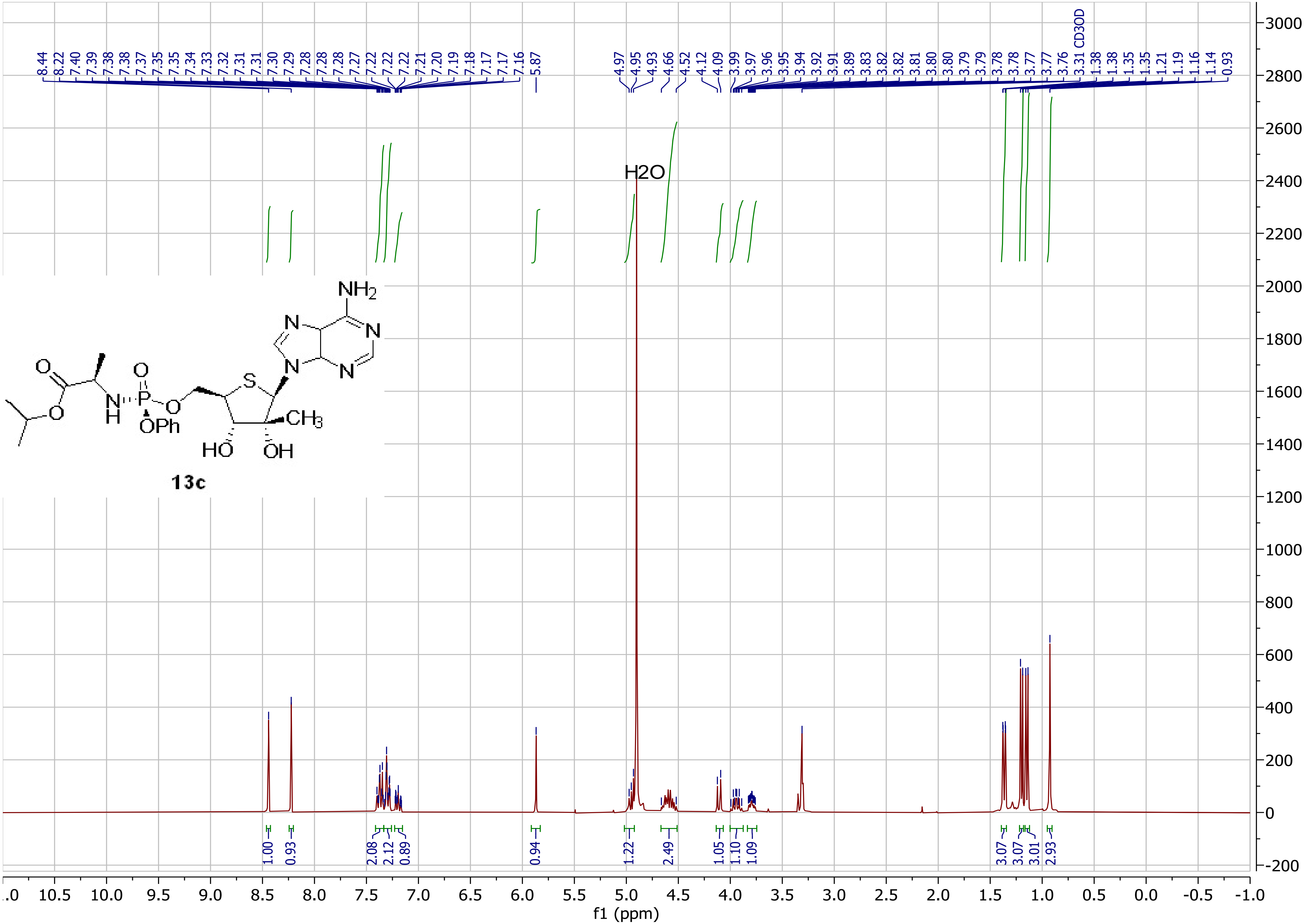

H2O
CD3OD
13c
f1 (ppm)

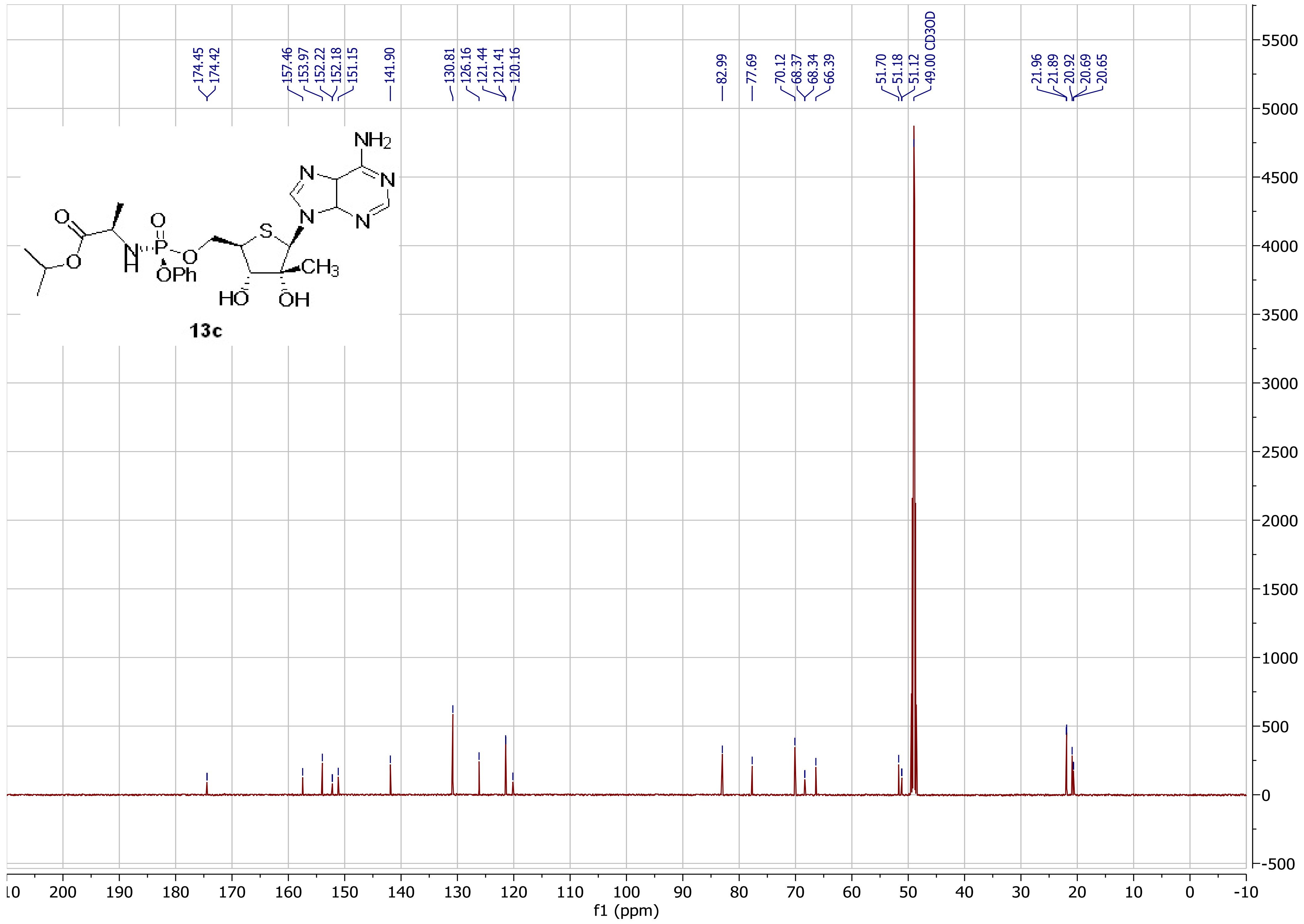
174.45
174.42
157.46
153.97
152.22
152.18
151.15
141.90
130.81
126.16
121.44
121.41
120.16
82.99
77.69
70.12
68.37
68.34
66.39
51.70
51.18
51.12
49.00 CD3OD
21.96
21.89
20.92
20.69
20.65
NH2
N
S
CH3
HO
OH
OPh
O
P
N
H
13c
f1 (ppm)

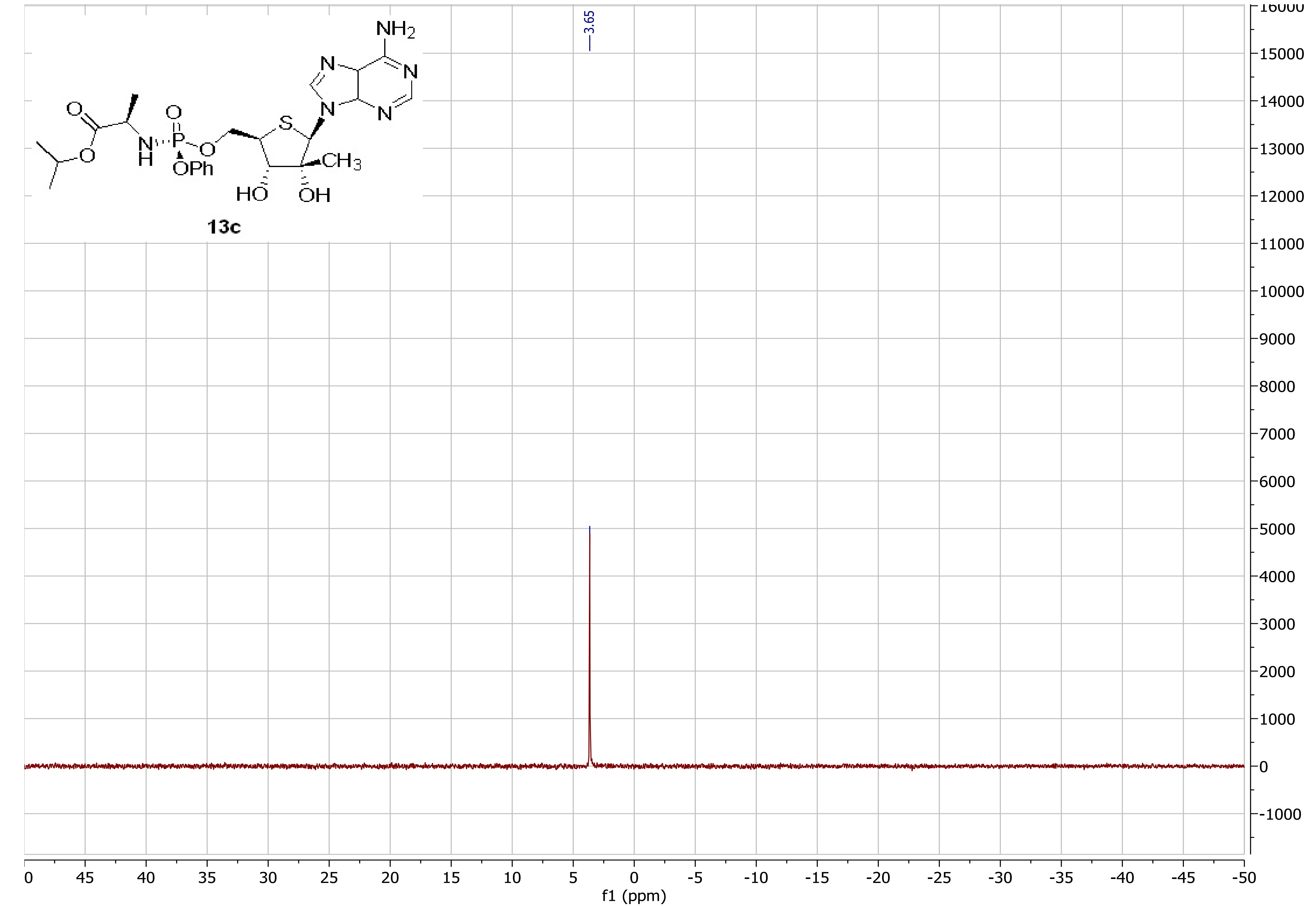

3.65
13c
f1 (ppm)

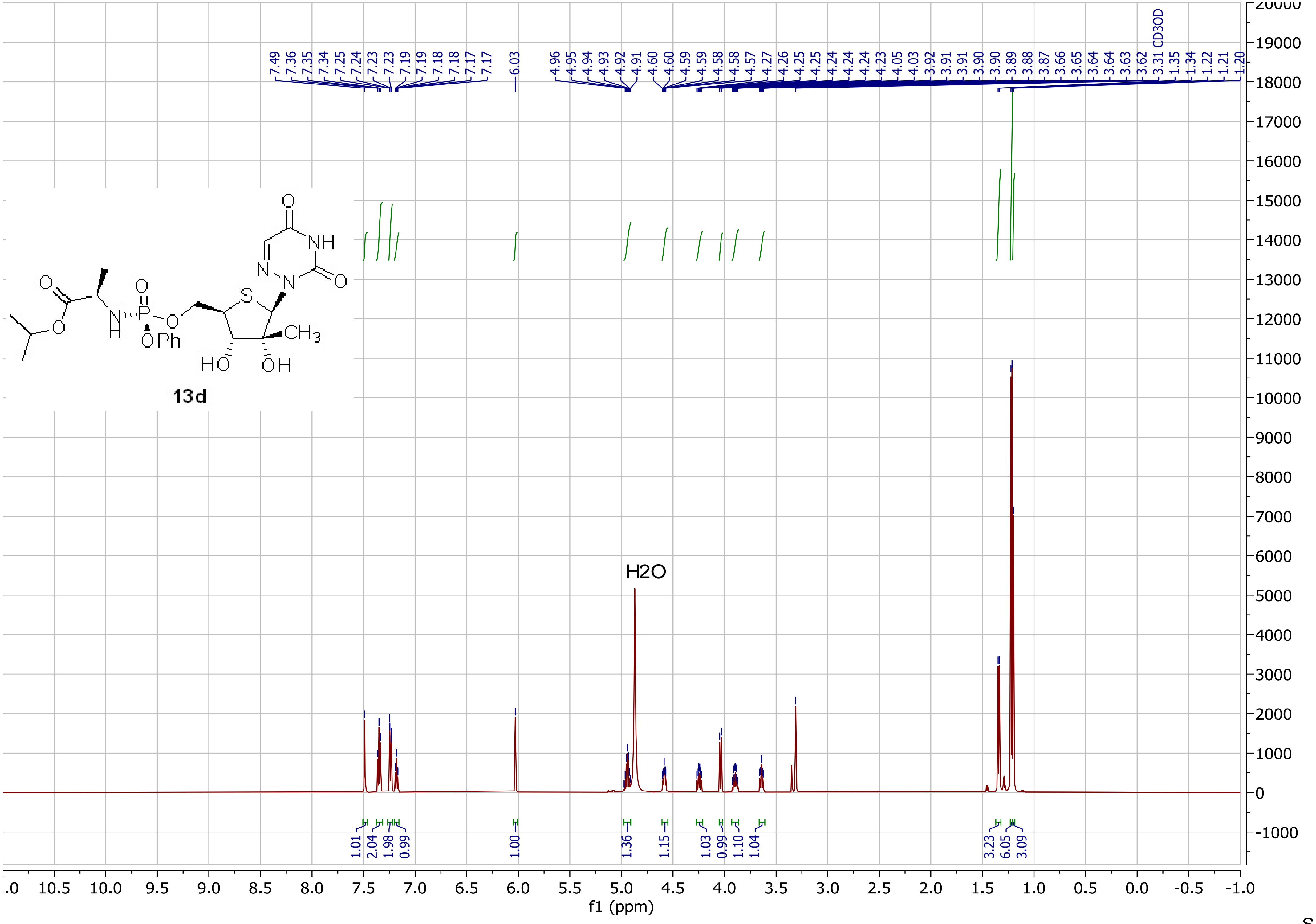

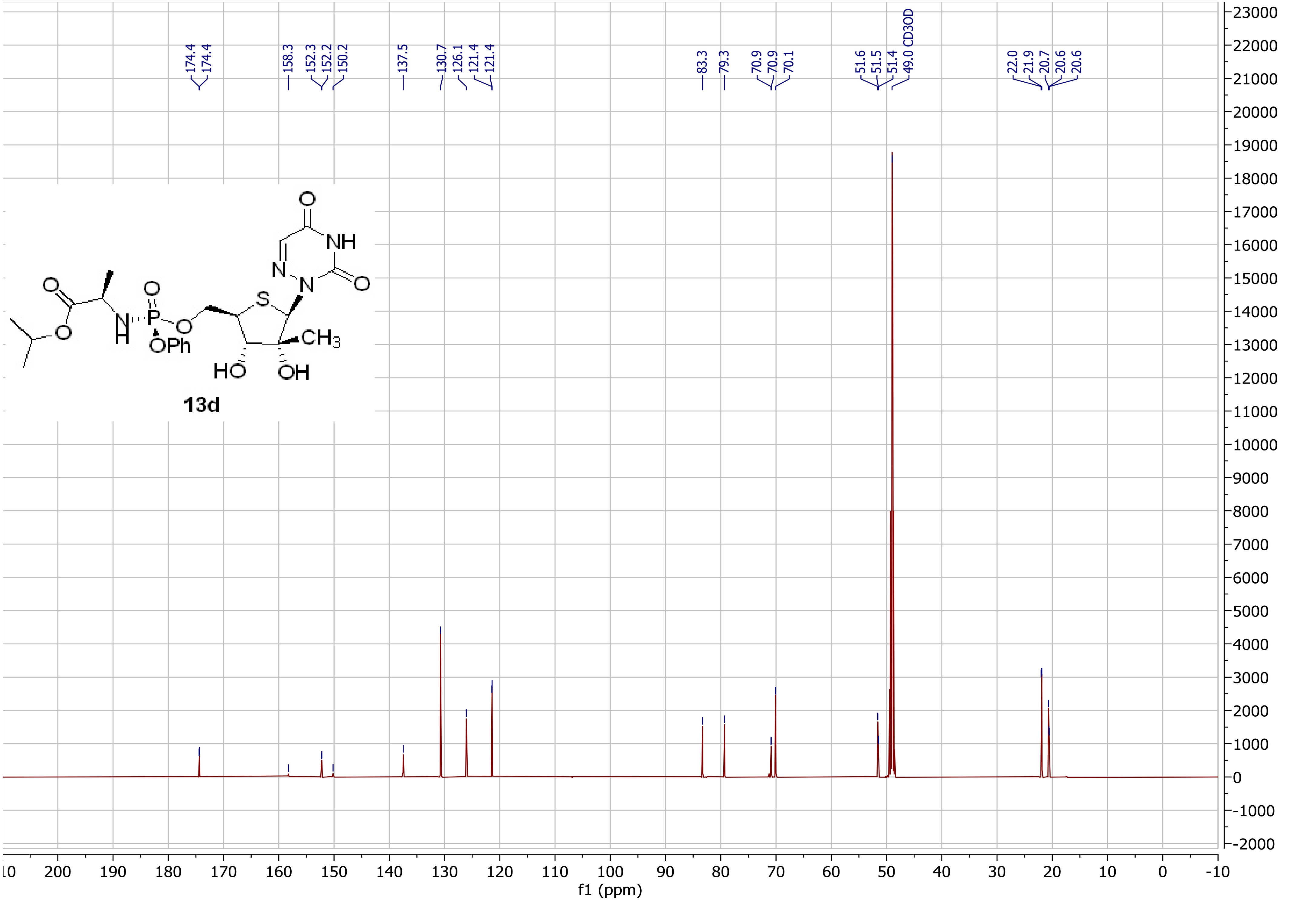
174.4
174.4
158.3
152.3
152.2
150.2
137.5
130.7
126.1
121.4
121.4
83.3
79.3
70.9
70.9
70.1
51.6
51.5
51.4
49.0 CD3OD
22.0
21.9
20.7
20.6
20.6
NH
N
S
CH3
OPh
HO
OH
f1 (ppm)

13d

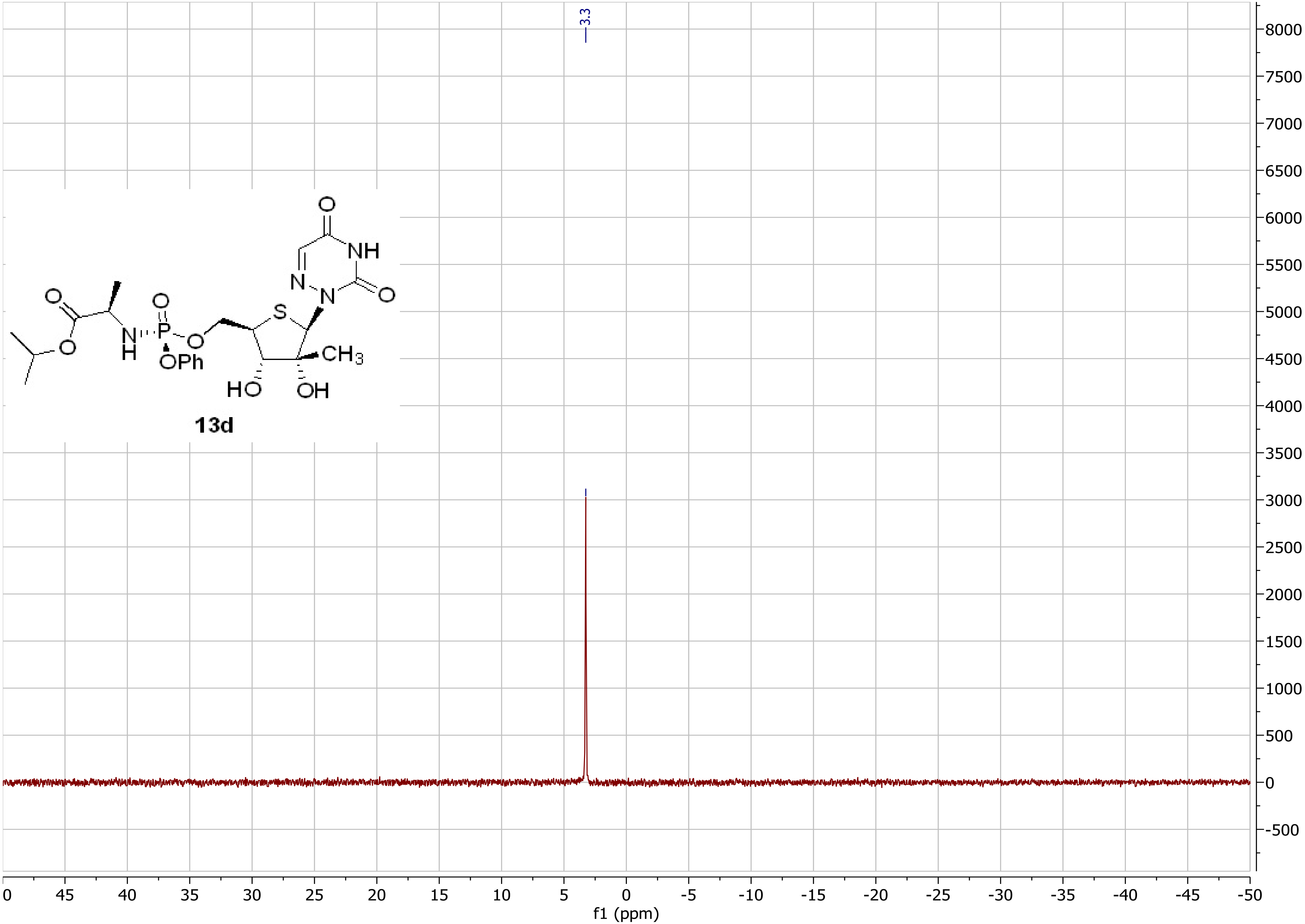
3.3
13d
f1 (ppm)

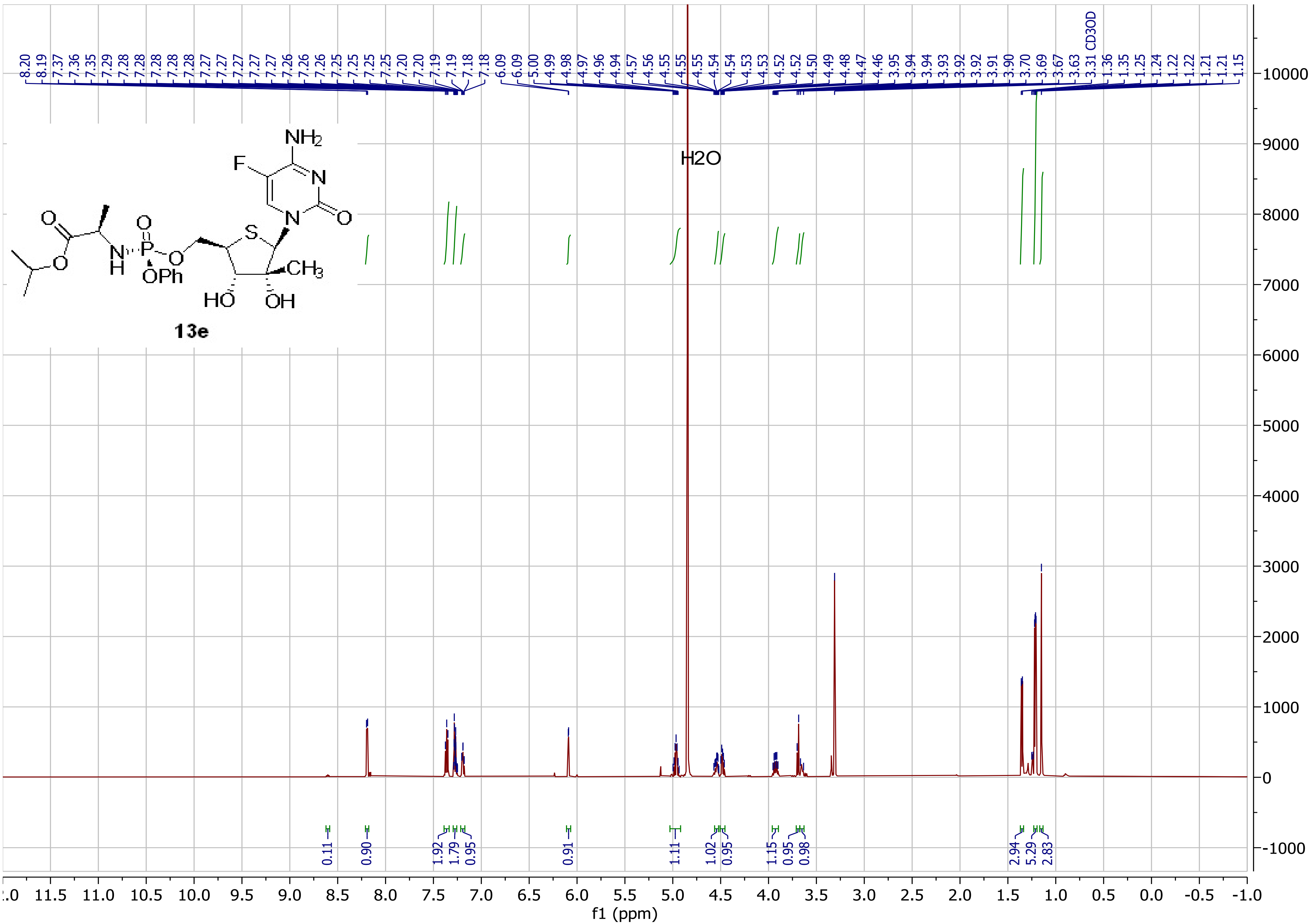

13e
H2O
CD3OD
f1 (ppm)

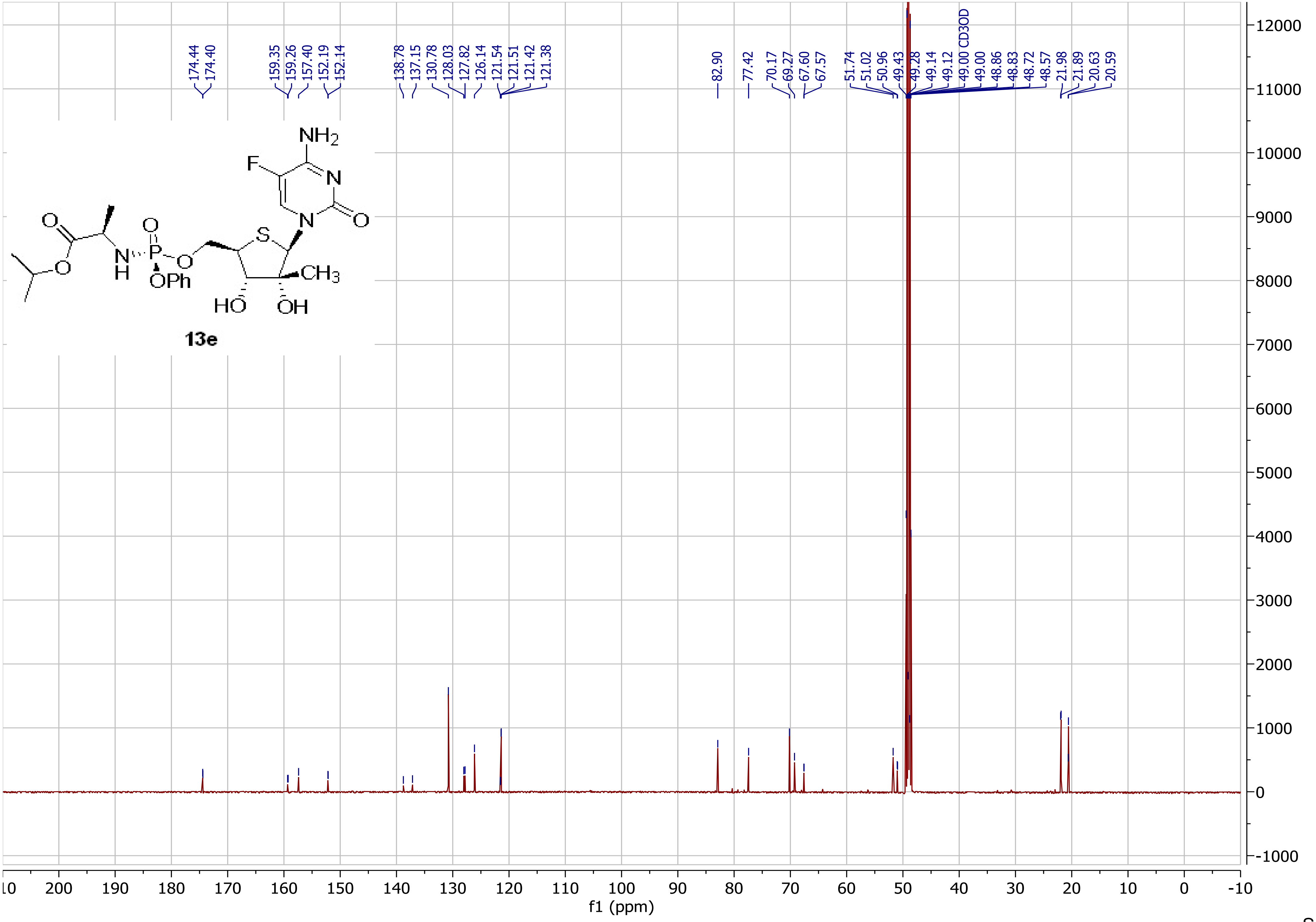

174.44
174.40
159.35
159.26
157.40
152.19
152.14
138.78
137.15
130.78
128.03
127.82
126.14
121.54
121.51
121.42
121.38
82.90
77.42
70.17
69.27
67.60
67.57
51.74
51.02
50.96
49.43
49.28
49.14
49.12
49.00 CD3OD
49.00
48.86
48.83
48.72
48.57
21.98
21.89
20.63
20.59
NH2
F
N
O
N
S
O
O
N
P
O
O
H
OPh
CH3
HO
OH
13e
f1 (ppm)

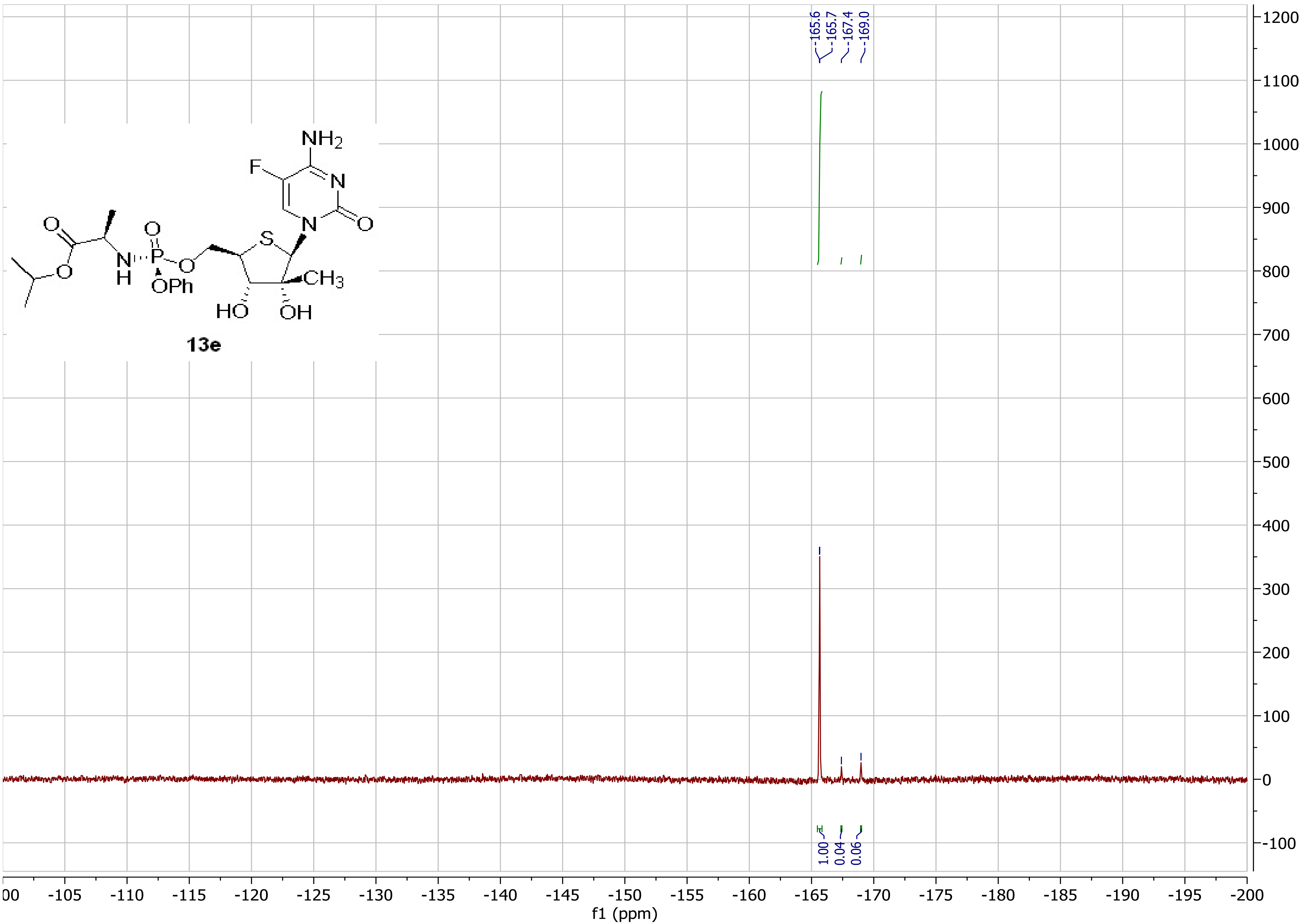

-165.6
-165.7
-167.4
-169.0
1.00
0.04
0.06
NH2
F
N
N
O
O
S
CH3
O
N
H
P
O
O
OPh
HO
OH
13e
f1 (ppm)

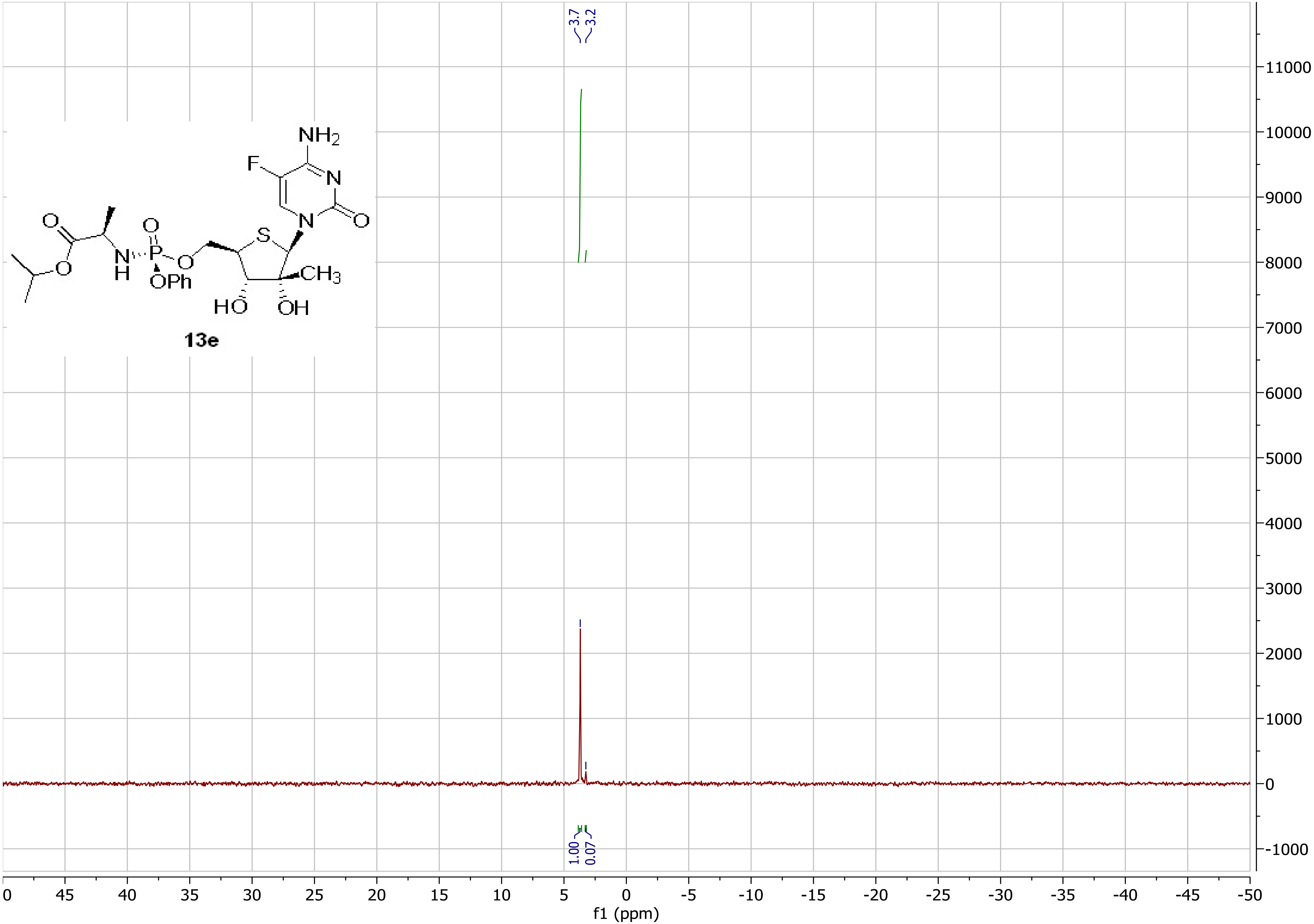

3.7
3.2
1.00
0.07
11000
10000
9000
8000
7000
6000
5000
4000
3000
2000
1000
0
-1000
0
45
40
35
30
25
20
15
10
5
0
-5
-10
-15
-20
-25
-30
-35
-40
-45
-50
f1 (ppm)

13e

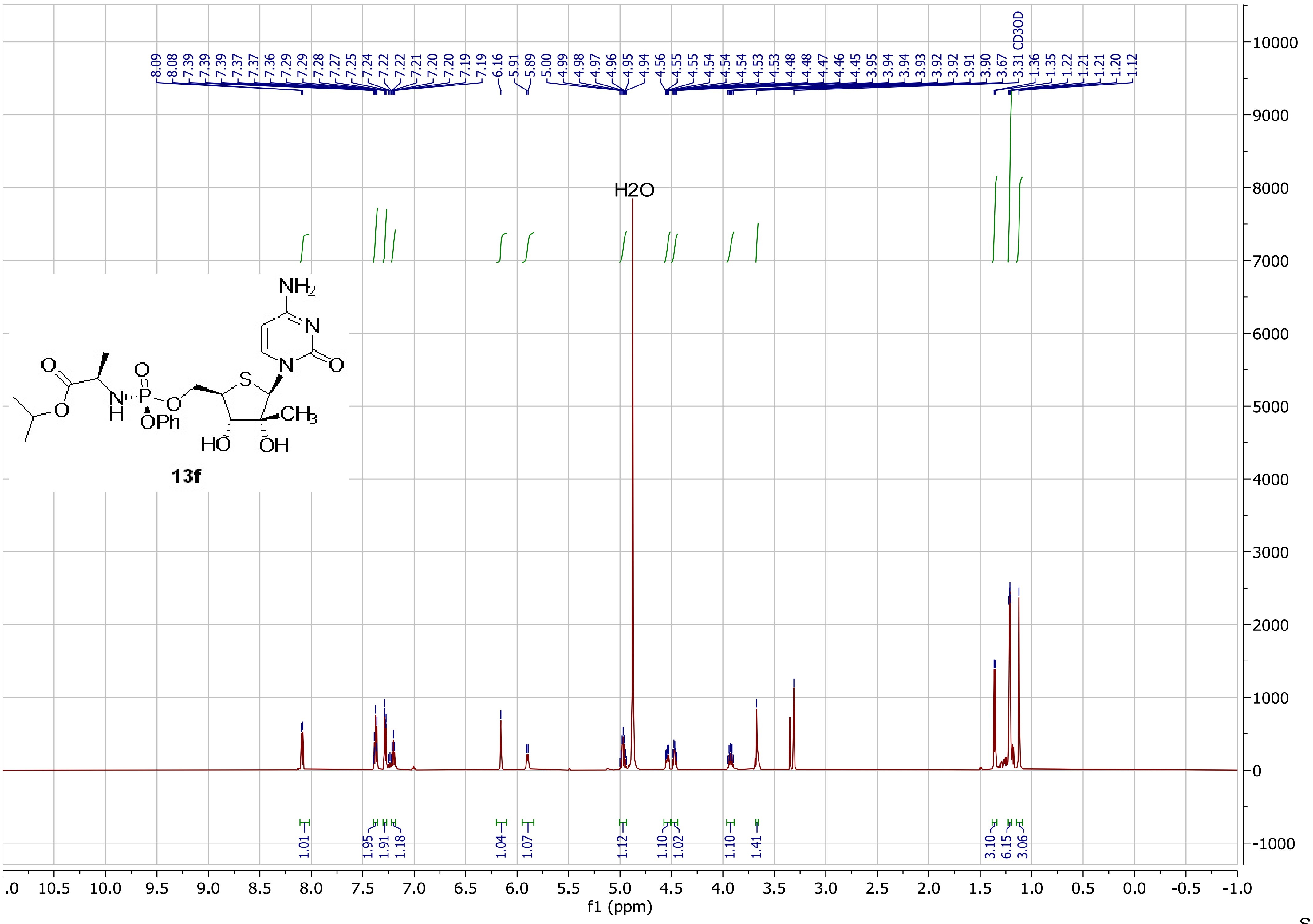

NH2
N
O
S
O
N
P
H
OPh
O
O
CH3
HO
OH
13f
H2O
CD3OD
8.09 8.08 7.39 7.39 7.39 7.37 7.37 7.36 7.29 7.29 7.28 7.27 7.25 7.24 7.22 7.22 7.21 7.20 7.20 7.19 7.19 6.16 5.91 5.89 5.00 4.99 4.98 4.97 4.96 4.95 4.94 4.56 4.55 4.55 4.54 4.54 4.54 4.53 4.53 4.48 4.48 4.47 4.46 4.45 3.95 3.94 3.94 3.93 3.92 3.92 3.91 3.90 3.67 3.31 1.36 1.35 1.22 1.21 1.21 1.20 1.12
1.01 1.95 1.91 1.18 1.04 1.07 1.12 1.10 1.02 1.10 1.41 3.10 6.15 3.06
f1 (ppm)

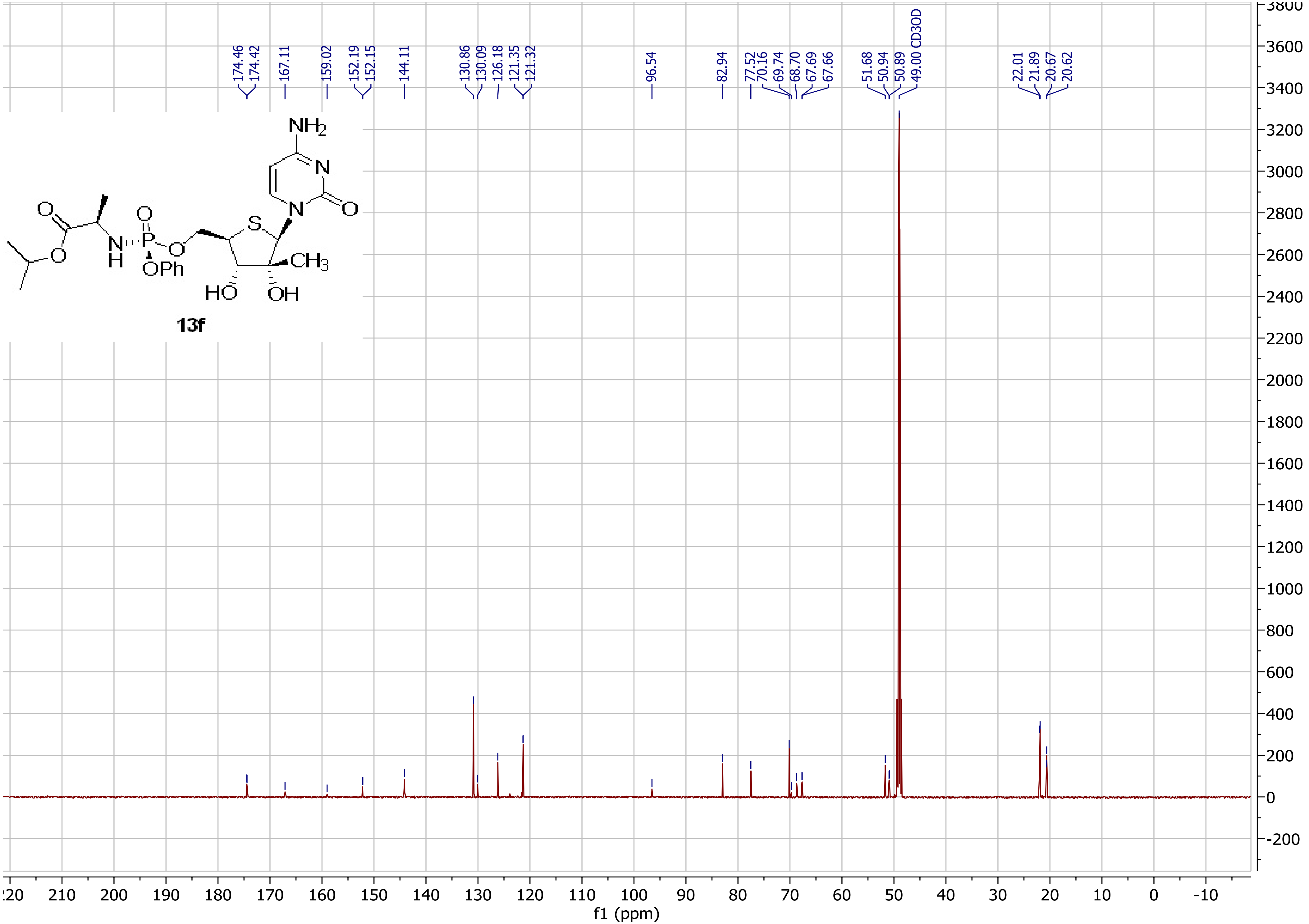
174.46
174.42
167.11
159.02
152.19
152.15
144.11
130.86
130.09
126.18
121.35
121.32
96.54
82.94
77.52
70.16
69.74
68.70
67.69
67.66
51.68
50.94
50.89
49.00 CD3OD
22.01
21.89
20.67
20.62
NH2
N
O
N
S
CH3
OH
HO
O
P
NH
OPh
13f
f1 (ppm)

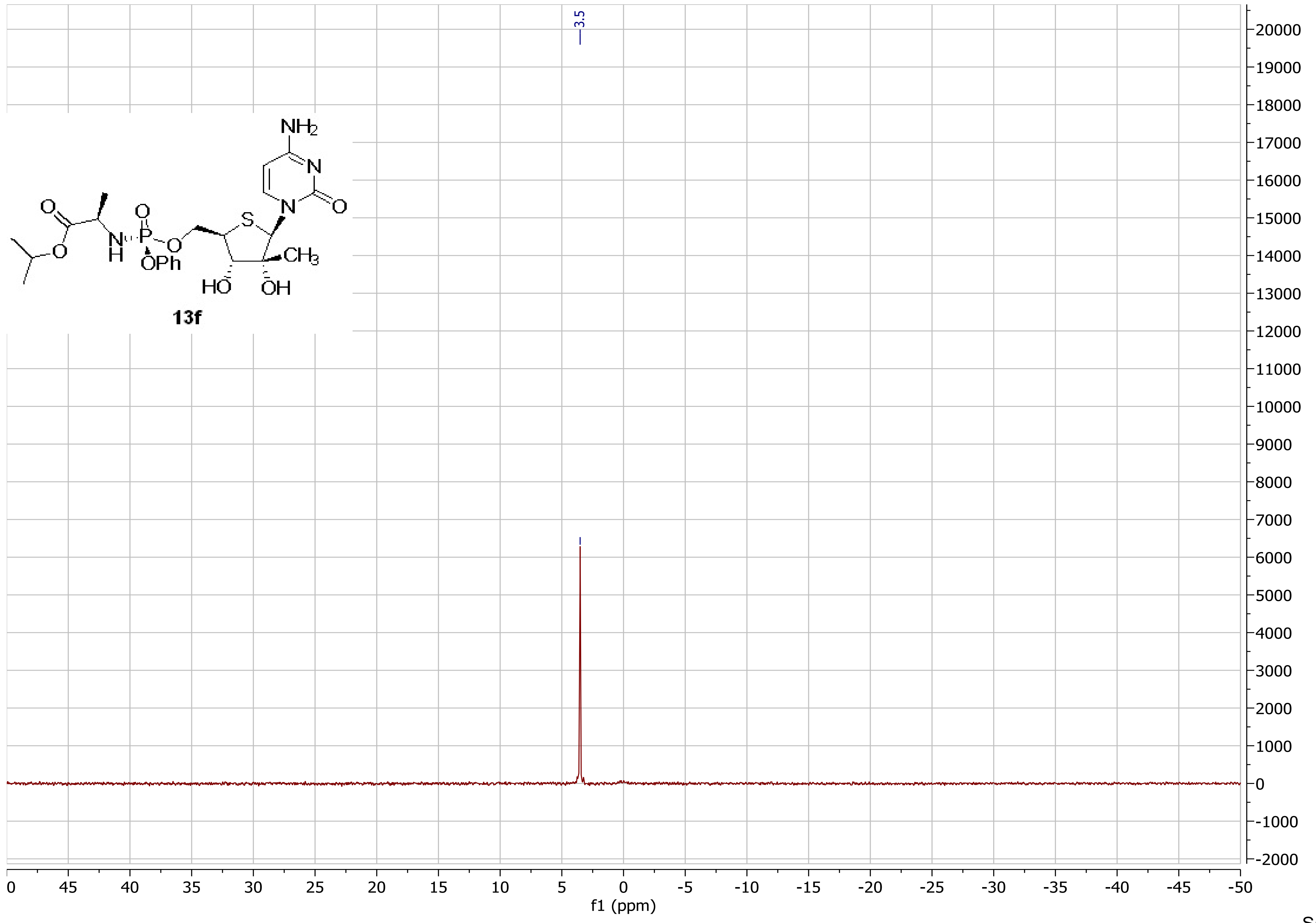
3.5
13f
f1 (ppm)
